# *In silico* serine β-lactamases analysis reveals a huge potential resistome in environmental and pathogenic species

**DOI:** 10.1038/srep43232

**Published:** 2017-02-24

**Authors:** Christian Brandt, Sascha D. Braun, Claudia Stein, Peter Slickers, Ralf Ehricht, Mathias W. Pletz, Oliwia Makarewicz

**Affiliations:** 1 Center for Infectious Diseases and Infection Control, Jena University Hospital, Jena, Germany; 2 InfectoGnostics Research Campus, Jena, Germany; 3 Alere Technologies GmbH, Jena, Germany

## Abstract

The secretion of antimicrobial compounds is an ancient mechanism with clear survival benefits for microbes competing with other microorganisms. Consequently, mechanisms that confer resistance are also ancient and may represent an underestimated reservoir in environmental bacteria. In this context, β-lactamases (BLs) are of great interest due to their long-term presence and diversification in the hospital environment, leading to the emergence of Gram-negative pathogens that are resistant to cephalosporins (extended spectrum BLs = ESBLs) and carbapenems (carbapenemases). In the current study, protein sequence databases were used to analyze BLs, and the results revealed a substantial number of unknown and functionally uncharacterized BLs in a multitude of environmental and pathogenic species. Together, these BLs represent an uncharacterized reservoir of potentially transferable resistance genes. Considering all available data, *in silico* approaches appear to more adequately reflect a given resistome than analyses of limited datasets. This approach leads to a more precise definition of BL clades and conserved motifs. Moreover, it may support the prediction of new resistance determinants and improve the tailored development of robust molecular diagnostics.

The secretion of antimicrobial compounds by microbes is an ancient and effective method to improve the survival of microbes competing for space and nutrients with other microorganisms^1^,^2^. Thus, the emergence of resistance mechanisms to antimicrobials is also an ancient natural response process^3^,^4^. The vast diversity of bacterial species coupled with short generation times and horizontal gene transfer permit the rapid accumulation of countless resistance variations at a relatively high evolutionary pace^5^. The environmental resistome has increased in diversity as microorganisms have adapted to environmental changes and has been disseminating for millions of years in response to interactions among bacteria, fungi, plants and animals^3^. Resistance in environmental bacteria^6^,^7^ typically goes unnoticed until a given species becomes of medical interest. However, the environmental resistome is also suspected to be a source of newly emerging resistance genes in the clinical environment^8^,^9^.

In the context of this alarming spread and the diversification and extension of the spectrum of β-lactamases (BLs) conferring resistance to frequently used β-lactam antibiotics that has been observed in recent decades in Gram-negative species^1^, the impact of the environmental resistome remains unclear. The BLs are a highly heterogeneous group of enzymes with more than 1,800 described variants that are classified either according to their protein sequence homology (Ambler classification)^10^ or their phenotypic profile (Bush-Jacoby-Medeiros classification)^11^,^12^. According to the Ambler classification, four weakly related classes can be distinguished: classes A, C, and D, representing the serine β-lactamases (Ser-BLs), and class B, containing the metallo-β-lactamases (MBLs). BL variants are commonly named using abbreviations to represent different origins, such as the most preferred β-lactam substrate of the first described variant of this group (e.g., CTX or OXA are derived from cefotaxime or oxacillin) or according to the names of persons and places corresponding to first detection (e.g., TEM is derived from the patient Temoneira; NDM is derived from New Deli MBL)^13^. It must be noted that neither the Ambler classification nor the BL group-label provide sufficient discriminatory power regarding phenotype^13^. Molecular classification correlates well with functional classification for the enzymes, which are divided into four groups (1 to 4) and multiple subgroups. However, because phenotypic profiles require experimental evaluation, most of the less clinically relevant variants are uncharacterized. BLs can be localized to chromosomes but are often associated with transposable elements and conjugative plasmids^14^. Horizontal interspecies transfer, additional masking mechanisms such as efflux or porin loss, and the common co-expression of various BL variants per cell represent major hurdles for routine culture-based phenotypic, diagnostic and molecular approaches^15^,^16^,^17^. The main limitation associated with molecular diagnostics is that clinically relevant variants are randomly distributed within phylogenetically unrelated BL classes and weakly conserved groups, thus hampering a molecular ‘one-fits-all’ assay.

The β-lactam antibiotics recommended for the treatment of Gram-negative infections are broad-spectrum penicillins, penicillins combined with β-lactamase inhibitors, cephalosporins, monobactams or carbapenems. Penicillins and cephalosporins were responsible for 55% of global antibiotic consumption in 2010^18^, leading to high selective pressure and the emergence of new BL variants. However, the massive increase in identified BL variants can only partially be explained by antibiotic use in the clinical or livestock farming sectors^19^ because many ESBL and carbapenemase variants have been detected in environmental strains^20^. Advanced sequencing technologies, such as next-generation sequencing (NGS), which enables rapid automated sequence analysis of even unculturable bacteria, have also contributed to the discovery of new variants. The steadily increasing quantity of sequence information that is stored in open-source databases is often not fully analyzed; therefore, it may contain unknown BL sequences that can potentially provide deeper insights regarding phylogeny and origin.

This work aimed to exploit the exponentially growing protein sequence databases that are currently available in the context of unknown Ser-BLs and their close relatives. All available protein sequences exhibiting motifs indicative of BL-like folding and the BL active site were collected in a non-redundant manner from various databases using the NCBI interface, which links millions of sequencing data. Phylogenetic analysis was performed considering the annotated and classified sequences of 1,886 BL variants by multiple and pairwise comparisons and an amino acid-based sequence similarity network to provide an update.

## Results and Discussion

### Molecular update of known β-lactamases

To obtain a revised overview of the of 1,886 currently known BL-variants, amino acid sequences were analyzed based on i) an amino acid-based sequence similarity network (SSN), ii) phylogenetic relationships, and iii) the extraction of highly conserved motifs found on Ser-BLs. The MBLs show relatively low sequence similarities but contain a conserved αββα sandwich structure with the active site being located at the external edge of the ββ region^21^. The active site possesses two potential Zn^2+^-binding sites with individual conserved amino acid residues that coordinate the binding of one (B2) (histidine site: His116, His118, His196) or two (B1, B3) (cysteine site: Asp120, Cys221, His236) Zn^2+^ moieties^21^; therefore, motif-based analysis of the MBLs remained unsuitable.

**Figure 1:**
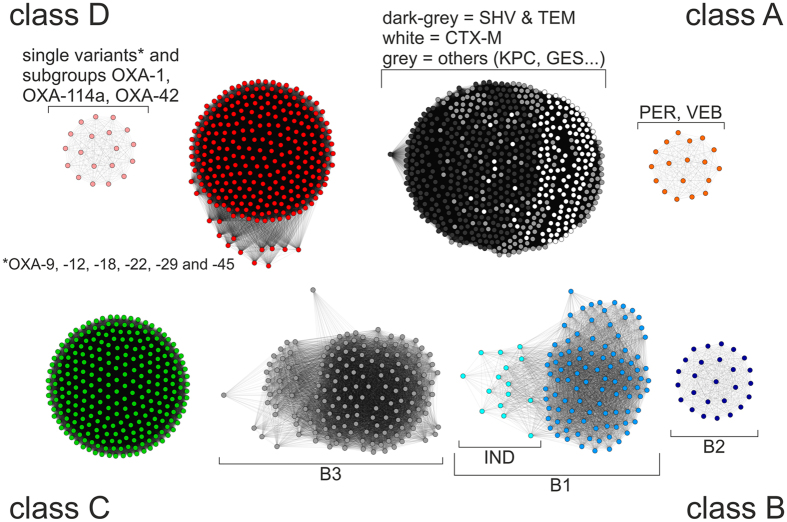
Amino acid-based SSN of 1,494 β-lactamase variants. Each dot represents a unique protein sequence. Connections between proteins represent minimal similarity based on ‘blast-all-versus-all’ e-values.

Using the SSN, the relationships of the proteins were visualized with respect to the molecular BL classes (Fig. 1). The class A BLs clearly formed two network clusters in which the PER and VEB BL groups formed their own cluster, which was distinctly separated from the other groups, indicating a large phylogenetic distance. The class B BLs formed three clusters that were consistent with the currently classified groups: B1, B2 and B3. However, larger intra-group variations were observed within groups B1 and B2, underscoring the high phylogenetic heterogeneity of the MBLs. Within the B1 cluster, mainly IND variants dissociated from the cluster, displaying a low level of similarity to other representatives of the B1 group. The molecular class C BLs revealed an equidistant network assembly of variants, indicating that the representatives of class C are closely related. Within the class D BLs, some variants –mainly narrow and ESBL types, including the OXA-1 group –were separated from the other OXA variants. Certain OXA variants showed a distinctively lower similarity but were still connected to the major OXA cluster.

**Figure 2:**
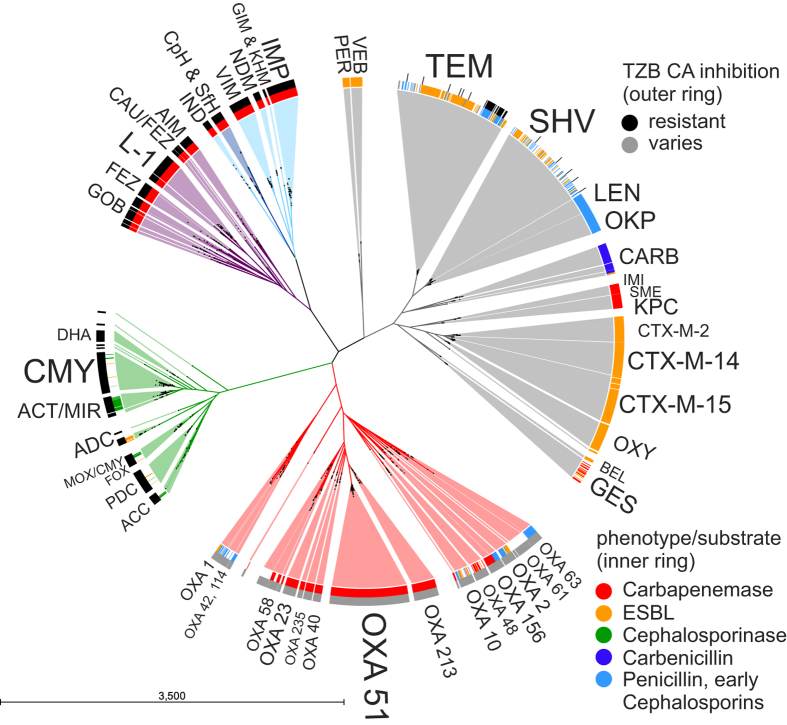
Radial phylogenetic circular tree including the 1,886 known β-lactamases. Classes are highlighted according to Ambler: A, grey; B1, light blue; B2, dark blue; B3, purple; C, green; D, red. The two rings around the tree indicate known β-lactam substrate profiles and resistance against inhibitors. The dots within the phylogenetic tree represent the positions of the variants within the tree. The dots may overlap, indicating very high similarity. Very small groups and single variants are not indicated, but detailed information is provided in Supplementary Tables 1 to 4. TZB = tazobactam, CA = clavulanic acid

The amino acid-based phylogenetic tree reflected the evolutionary relationship of the known variants and revealed the clades (Fig. 2). Functional classification based on hydrolytic profiles and inhibitor susceptibility (tazobactam or clavulanic acid), if known, was implemented in Fig. 2 for illustrative purposes, but it remained less descriptive for the classification of BL variants, groups, and even most classes (except for class B, which can be differentiated from the Ser-BLs due to the inhibition by EDTA; this phenotype was not included in Fig. 2).

The large phylogenetic distances between the classes clearly demonstrated their divergent polyphyletic origin and consequent convergent evolution. Within the Ser-BL classes, major clades with different ancestors could be defined, which in most cases fit the Ambler nomenclature. Considering the sequence similarities between the variants and groups, some refinements appeared to be useful, particularly for classes A and D.

In total, 638 different amino acid sequences could be designated to Ambler class A, revealing distinct, highly conserved groups with >80% sequence identity (for a detailed overview, see Supplementary Table 1) but lower conservation between groups. As previously observed in the SSN, the ESBLs of the PER and VEB groups formed a separate clade that displayed a large phylogenetic distance compared with the other class A groups, supporting its convergent evolution. This clade was recently named class A2 to differentiate it from the other groups that were combined to form class A1^22^. The KPC/IMI/SME and GES/BEL groups bear the only known carbapenemases within class A, forming two separate and non-related clades and indicating that these carbapenemases emerged independently. Interestingly, BEL BLs were characterized as ESBLs despite their close phylogenetic proximity to the GES group containing many carbapenemases (at least 50% sequence identity). The CARB variants (carbenicillinases) formed a clade with three different CARB subgroups (CARB-1, -5 and -17 groups). The major ESBL clade combining the CTX-M group and OXY group exhibits only 70% amino acid identity within their variants and thus can be further subdivided into the more conserved subgroups CTX-M-2, -8, -9, -15 and OXY (>88% identity). The OKP-A/OKP-B and LEN variants are clearly related to each other, sharing at least 78% amino acid identity and similar narrow-spectrum hydrolytic activity; they can be merged into one group that shares an ancestor with SHV (82%). The TEM group formed its own clade, which is more closely related (60–63% identity) to the SHV/LEN/OKP clade.

Because of low sequence similarity between the MBL subgroups and their relatively equal sequence similarity to other Zn^2+^-dependent enzymes, the nomenclature used for MBLs (class B) is based solely on structural rather than sequence similarity^23^. Based on amino acid identity, class B can be grouped into two distinct phylogenetic clades (B3 and B1/B2), but the SSN illustrating their strong molecular diversification supports a subdivision into three clades and classes (for a detailed overview, see Supplementary Table 2).

Class C BLs form six major AmpC groups. The largest clade was named CMY because it contains CMY variants; however, this clade also contains many variants that were erroneously assigned as MOX variants. The clade named MOX/CMY in Fig. 2 contains variants that are distinct from the CMY clade. Therefore, it is useful to rename these confusing CMY and MOX variants to avoid misinterpretations. The variants known as ACT and MIR exhibit a strong relationship (at least 85% sequence similarity), forming one clade. Other smaller clades are the DHA, FOX, ACC and ADC/PDC clades. There were also some very small clades with only a few variants. These clades are not explicitly referenced in Fig. 2, but detailed information is provided in Supplementary Table 3. Class C bears many intrinsic BLs, but the phylogenetic tree and SSN were mainly constructed with plasmid-localized variants, which are primarily found in clinically relevant pathogens. Thus, the phylogeny based on the known class C BLs does not reflect the diversity of the whole class C.

Within the class D BLs, the overall similarity was less than 40%. Therefore, a revised OXA group nomenclature that better reflects the phylogeny of this class has recently been proposed by Evens and Amyes^24^. The present phylogenetic analysis supports this idea but suggests two corrections: i) the OXA-143 subgroup displayed 87% identity with the OXA-40 group and ii) the OXA-134a subgroup showed 84% identity with the OXA-235 group; thus, these subgroups should be merged accordingly. Detailed information is provided in Supplementary Table 4.

**Figure 3:**
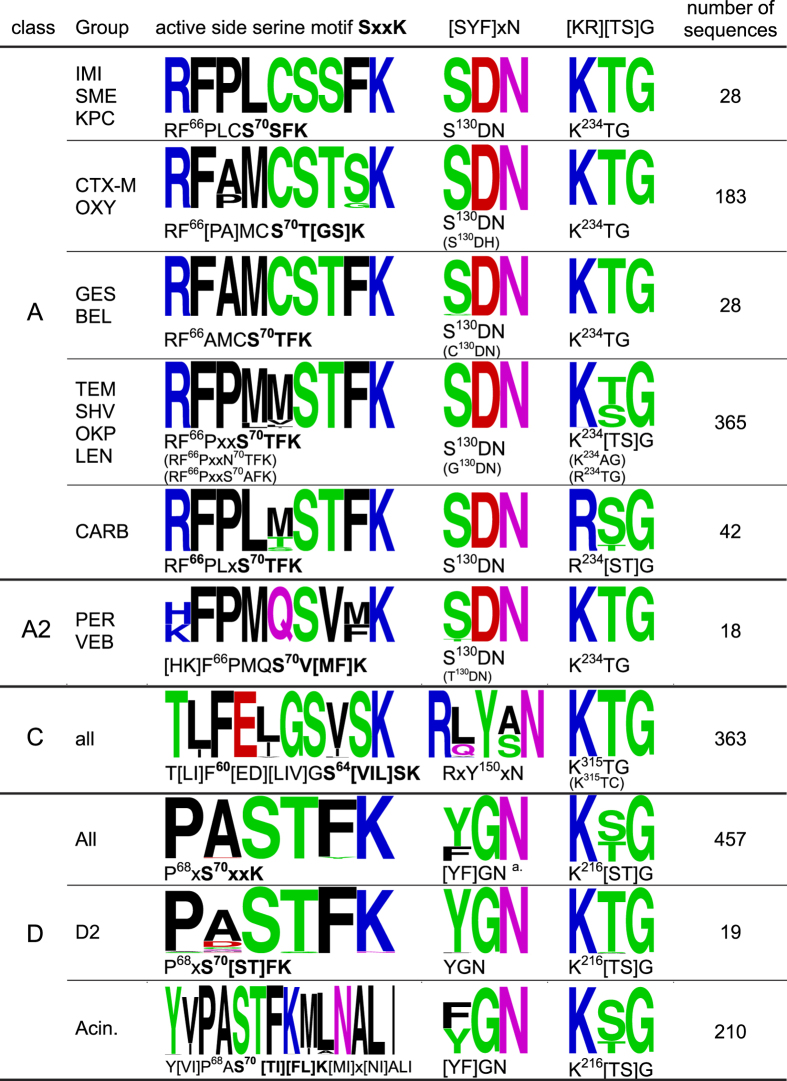
Overview of conserved serine β-lactamase motifs responsible for catalytic activity or substrate binding. Amino acids are colored according to their chemical properties: polar = green, basic = blue, acidic = red, and hydrophobic = black. ( ) = rare variants; [ ] = variations; x = more than 3 variations; a. many single variations can be observed (e.g., YGE, YGD, YRG, YKN, and YGS). Acin = *Acinetobacter* related.

Based on the proposed refined clades, the three characteristic conserved motifs for all Ser-BL sequences, namely, the active Serine-site SxxK motif, the [SYF]xN motif and the [KR][TS]G motif, were reanalyzed^10^. The variable amino acid residues in the active-site SxxK motif exhibited preferences depending on class or clade, and the conserved region could be expanded upstream by 5 amino acid residues for class A and A2, by 6 amino acid residues for class C, and by 2 amino acid residues for class D (see Fig. 3). The conserved active site of the class A BLs can be generalized as R^65^FxxxS^70^xxK. In class A2, the arginine at position 65 is replaced with histidine or lysine. In class D BLs, the conserved active site is shorter for P^68^xS^70^xxK. The second motif, S^130^DN, is also conserved in class A and A2 BLs but is altered in certain variants, as indicated in Fig. 3 (in brackets). In class D, this motif and its position exhibit larger differences but may be described as [YG]GN for most OXA variants. In class C, the second motif is RxY^150^xN, which shows a greater resemblance to the [YG]GN of class D. The third motif, often described as K[TS]G, is conserved for all Ser-BLs excluding the CARB clade, in which it is altered to R[TS]G.

### The potential β-lactamase reservoir

To identify ‘hidden’ BLs or their close relatives, all sequences stored in databases categorized in the ‘Ser-β-lactamase-like superfamily’ by the PFAM^25^ clan were analyzed. The PFAM identifies homologous proteins via a hidden Markov model (HMM) that compares them against a curator-defined dataset of representative protein families. In this manner, 112,727 unique sequences of the ‘Ser-β-lactamase-like superfamily’ were retrieved from the NCBI database and further annotated using a PERL script (see Supplementary Dataset File) and BLAST. In total, 9,681 sequences were found to be strongly related to the currently known BLs, among which 4,917 (including 1,082 subsequences) could be assigned to the clade of Ambler class A, 2,383 (including 537 subsequences) to the clade of class C and 2,381 (including 538 subsequences) to the clade of class D. In addition, 433 BlaR receptor proteins were assigned into the last clade; among which the extra-cellular domain is structurally similar to the OXA BLs, indicating a common ancestor. An additional 30,335 penicillin-binding proteins (e.g., PBP2a and PBP3), 15,454 D-Ala-D-Ala carboxylases, 10,380 serine hydrolases (mainly the EstA family) and 16,077 hypothetical proteins were assigned to the ‘Ser-β-lactamase-like superfamily’ but were not phylogenetically related to the known Ser-BLs and were excluded from further analyses. Other non-Ser-BLs-related enzyme groups found in this PFAM superfamily were 6-aminohexanoate-dimer hydrolases, esterase variants, peptidoglycan synthases (FtsI-like) and glutaminase variants, which were also excluded from the analyses. In the following chapters, the three Ser-BL clades, including the newly identified relatives, are analyzed in detail.

### Class A

**Figure 4:**
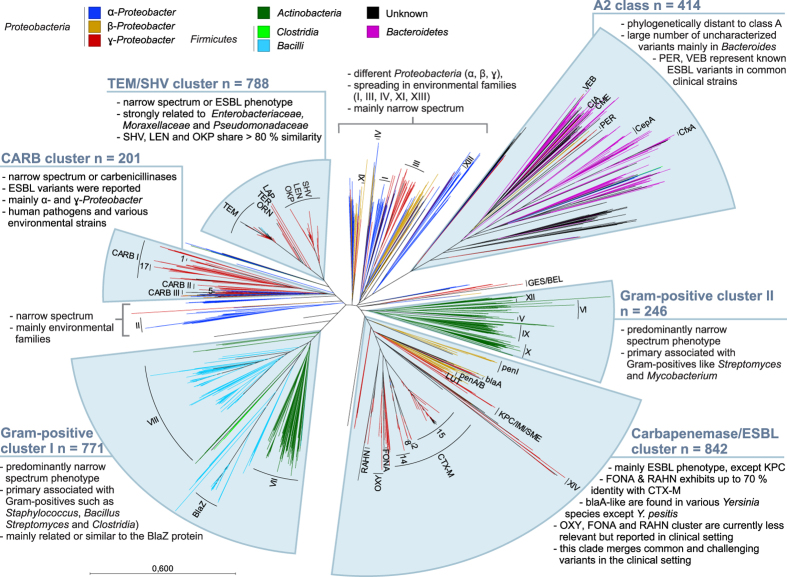
Radial phylogenetic tree of 3,835 class A-related β-lactamase variants. The tree is clustered for illustrative purposes. Only major clades were named according to known nomenclature or with Roman numbers if unclassified. Branches are colored according to the phyla. More details are provided in Supplementary Table 5.

In addition to the clinically relevant Ser-BLs of the class A-like proteins, further clades could be identified as related to four phyla of *Proteobacteria, Firmicutes, Actinobacteria* and *Bacteroidetes* (Fig. 4). Those clades were numbered consecutively in roman numerals (without any specific context) when containing at least 10 variants; otherwise, they are not indicated in Fig. 4, but details are provided in Supplementary Table 5. Considering their distribution in the phyla and the phylogenetic nodes, class A-related proteins could be subdivided into 6 major clusters (the TEM/SHV, CARB, carbapenemase/ESBL, two Gram-positive clusters and the A2 class) that serve to simplify the highly complex phylogenetic overview to achieve a reliable illustration of the phylogenetic relationships.

The SHV/TEM cluster contains the TEM and SHV/LEN/OKP clades and several groups like LAP, TER, ORN and PLA, which are closely related to ɣ-*Proteobacteria* such as *Enterobacteriaceae, Moraxellaceae* and *Pseudomonadaceae*; thus, the TEM variants appear to have a broader host spectrum (see Supplementary Dataset File). The TEM and SHV/LEN/OKP variants represent the oldest described BLs that differentiated into ESBLs in clinically relevant pathogens^26^, and their subsequent relatives exhibit less homology, bearing only small groups with a rather narrow (e.g., variants of LAP were found in clinical *Klebsiella pneumoniae* strains^27^ and in various *Enterobacter* strains) or unknown substrate spectrum. The small groups PLA, ORN and TER contain BLs that appear to be primarily associated with various *Raoultella* species: PLA in *R. planticola*, ORN in *R. ornithinolytica*, and TER in *R. terrigena.* Carbapenem resistance in *Raoultella* has been reported but is related to the plasmid-localized class D BL OXA-48 and not to TER, PLA or ORN^28^. However, ESBL resistance associated with PLA has been reported^29^. Driven by point mutations, additional new ESBL variants may evolve, most likely in the clinically relevant TEM and SHV clades.

The CARB cluster contains 201 sequences (divided into 3 main lineages CARB I to III), including the groups CARB-1, -17, and -15 as well as further described carbenicillinases such as BRO, HER and AER and many other narrow-spectrum penicillinases. These sequences were found in both α- and ɣ-*Proteobacteria*, as well as within these classes in various families, including in human pathogens (e.g., *Enterobacteriaceae* and *Pseudomonadaceae*) and environmental isolates (e.g., *Rhodospirillaceae* and *Psychromonadaceae*), indicating early evolutionary branching, family transmission and diversification. Because this cluster contains mainly narrow-spectrum and carbenicillinase variants and only few ESBLs, the emergence of new carbapenemases within this cluster is highly unlikely.

The carbapenemase/ESBL cluster contains clades of CTX-M, OXY, FONA, RAHN, penI, penA/B, blaA and clade XIV and KPC/IMI/SME, which are mainly found in ɣ-*Proteobacteria*, although some are associated with β-*Proteobacteria*. The CTX-Ms are widespread in common Gram-negative human pathogens such as *Enterobacteriaceae, Moraxellaceae* and *Pseudomonadaceae*, whereas variants of FONA, RAHN and OXY are primarily spread in *Enterobacteriaceae*. Groups of penA/B and penI were primarily found in the intrinsically resistant and difficult to threat genus *Burkholderia.* The FONA and RAHN ESBLs have mainly been isolated from *Serratia fonticola* (usually isolated from the rhizosphere of pea roots) and *Rahnella aquatilis* (usually found in fresh water), respectively. Some variants of FONA and RAHN display up to 70% sequence identity to the CTX-Ms. These ESBLs are rarely isolated in clinical specimens; however, wound infections caused by *R. aquatilis* have been observed^30^. OXY-ESBLs were found in *Klebsiella oxytoca,* which may cause antibiotic-associated hemorrhagic colitis^31^. ESBL variants of clade blaA were found in *Yersinia* (especially in *Y. enterocolita,* which can cause yersiniosis) and *Erwinia* species (a plant pathogen). Plasmid-mediated antibiotic resistance in *Y. pestis* – the causative agent of plague – has been observed^32^; however, a clade blaA-like resistance gene for this pathogen could not be found in the NCBI databases. Interestingly, the CTX-Ms have three main lineages (CTX-M-8 (includes -2), -14 and -15) that branched earlier and began to diversify, probably due to antibiotic-selective pressure. In contrast, the lineages of clade blaA, penA/B, penI FONA, RAHN and clade XIV bearing many variants that seem to branch and to diversify earlier suggested a natural antimicrobial resistance phenotype that was unlikely to be driven by cephalosporin use in healthcare. Due to their presence in related species and shared habitats, the transmission to human or animal pathogens of variants of FONA/RAHN or clade X remains possible but has not yet been reported. It is also unclear if new carbapenemases are present in these clades because despite the very low carbapenemase activity of FONA in *Serratia fonticola*, it is still categorized as susceptible according to current clinical breakpoints^33^.

The carbapenemases in clade KPC/IMI/SME were found in different genera of ɣ-*Proteobacteria* (primary IMI in *Enterobacter*, KPC in *Klebsiella* and SME in *Serratia*) but show nearest phylogenetic distance to the groups of LUT ESBLs (50% sequence identity to KPC), SFC carbapenemases (63% sequence identity to KPC) and BIC carbapenemases (40–57% sequence identity to KPC). The KPC lineages branch relatively early from the IMI and SME lineages, and narrow-spectrum variants have not yet been described in any of the lineages, suggesting that the carbapenemase phenotype of these variants emerged independently of carbapenemase use and was introduced to pathogens from environmental sources.

The variants of group GES/BEL were found in common pathogen classes of ɣ-*Proteobacteria* such as *Enterobacteriaceae, Moraxellaceae* and *Pseudomonadaceae*, suggesting divergent evolution. It is unclear if the carbapenemase phenotype of the GES variants developed in response to selective pressure or naturally.

The A2 class includes the clinically relevant PER and VEB variants, which formed a phylogenetic outgroup showing almost no relationship to the remaining class A BLs. Class A2 harbors a large number of uncharacterized variants and is predominantly associated with the phylum *Bacteroidetes*, which contains many anaerobic families that colonize the gut. Thereby, 103 of 414 different protein sequences were solely identified in *Bacteroides* genus. However, the PER and VEB variants, which share nearly 60% sequence similarity, were only found in ɣ-*Proteobacteria*, including common pathogens (*Enterobacteriaceae, Moraxellaceae* and *Pseudomonadaceae*), supporting the occurrence of early phylum transmission likely triggered by the shared gut habitat. Despite transmission to opportunistic species of *Bacteroides sp.*, ESBLs were not described. Thus, class A2 might represent a potential pool of ESBLs.

The last two cluster, a pool of 1,017 different proteins (clades of V, VI, VII, VIII, IX, X, and XII and BlaZ) with narrow-spectrum activity, is primarily associated with Gram-positive species (*Firmicutes* and *Actinobacteria*). The Gram-positive clade bears pathogenic families, such as *Staphylococci* and *Clostridia* (within *Firmicutes*) or *Mycobacteriaceae* and *Nocardiaceae* (within *Actinobacteria*). The presence of class A-related proteins illustrates that in addition to the altered penicillin-binding proteins (PBPs) (encoded by, e.g., *mecA, mecC)*, penicillinases are widespread within *Firmicutes and Actinobacteria* and may contribute to their overall β-lactam resistance.

The variants of clades I, II, III, IV, XI and XIII were found in different *Proteobacteria*. They show no relationship with other BLs and are mainly associated with soil-living non-pathogenic bacteria belonging to the orders *Rhizobiales, Xanthomonadales, Sphingomonadales* and *Burkholderiales*. Those BLs might translocate to clinically relevant species; however, this remains unlikely, particularly because there is no selective pressure for those narrow-spectrum variants.

### Class C

Within the class C BLs, often called AmpC, many variants confer an ESBL or narrow-spectrum resistance phenotype restricted mainly to early cephalosporins (cephalosporinase), but weak hydrolytic activity against carbapenems has already been described for some variants (CMY-2, CMY-10, ATC-1 and ADC-68)^34^,^35^. Moreover, increased carbapenem resistance associated with ESBL variants can be observed if secondary resistance mechanisms like porin loss are present^36^.

**Figure 5:**
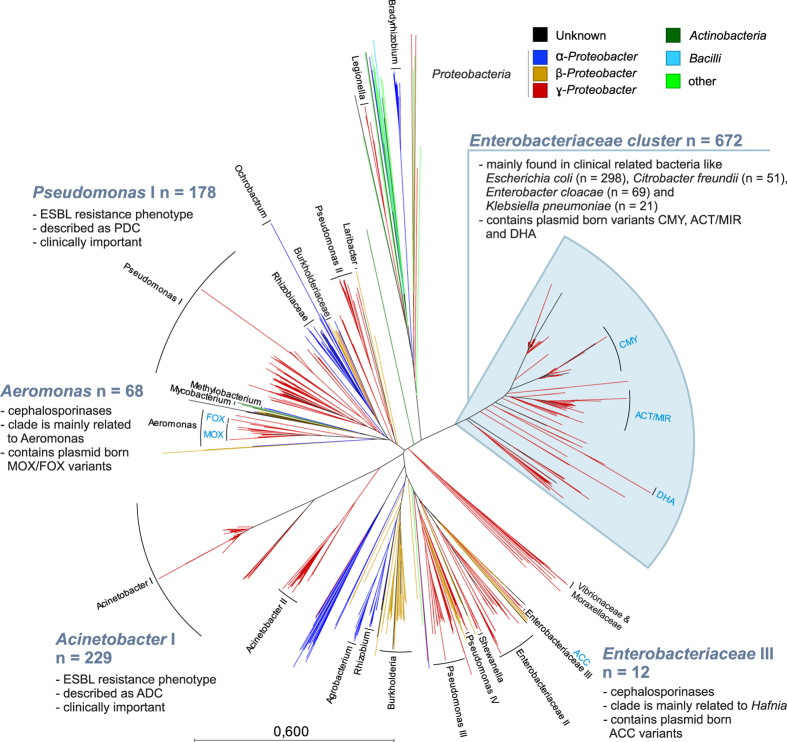
Visualization of 1,851 class C β-lactamase sequences as a radial tree. Plasmid-born variants are labeled in blue (CMY, ACT/MIR, DHA, ACC, FOX and MOX); branches are colored according to the organism (class). The tree is clustered for illustrative purposes. Major clades are labeled according to their lowest common taxonomy. Details are provided in Supplementary Table 6.

AmpC BLs are usually genera-specific and chromosomally located^37^; thus, the classification of AmpCs based on the corresponding bacterial genus or family remains reasonable. Contrary to the SSN and phylogenetic analyses based on the known variants, the whole class C including the relatives (n = 1,846) is rather highly diverse, and the differentiation of clades has been more difficult due to the low sequence similarities. This phylogenetic analysis of class C showed that they are widespread in a multitude of Gram-negative bacteria, mainly in α-, β- and ɣ-*Proteobacteria* (Fig. 5), in an environmental context (for details see Supplementary Table 6 and Supplementary Dataset File). Only one larger clade containing AmpC with highly diverse origins, including the small clades of ‘*Legionella*’ and ‘*Bradyrhizobium*’, demonstrated the occasional isolation of AmpCs from Gram-positive *Bacilli* and *Actinobacteria (Mycobacterium*). However, this clade bears 68 different variants that exhibit low sequence identity (only 10 to 20%), indicating rather early branching and divergent evolution.

Two clades of AmpCs associated with *Acinetobacter* (281 sequence variants, 20 different *Acinetobacter* species) were differentiated based on their sequence identity: one larger clade with 229 variants (‘*Acinetobacter* I’), which was mainly identified in *A. baumannii* and *A. pittii* known as ADC (*Acinetobacter*-derived cephalosporinases), and a smaller clade with 52 variants (‘*Acinetobacter* II’), which was mainly identified in *A. haemolyticus* and *A. junii* (both pathogens are of lower clinical impact).

Four clades related to *Pseudomonas* can be identified with preferences to different species. Clade ‘*Pseudomonas* I’ includes clinically relevant species (*P. aeruginosa* and *P. fluorescens*) and contains 178 different ESBL variants known as PDCs (*Pseudomonas*-derived cephalosporinases). The smaller clades do not seem to be related to *P. aeruginosa* and lack clinical relevance: ‘*Pseudomonas* II’ has 39 variants, mainly *P. putida* (nonpathogenic soil bacterium); ‘*Pseudomonas* III’ has 61 variants, mainly *P. syringae* (a plant pathogen); and ‘*Pseudomonas* IV’ has 7 variants with no predominant species.

Plasmid-born AmpC clades (names labeled in blue in Fig. 5) were identified only in *Enterobacteriaceae* and *Aeromonas*. In the largest *Enterobacteriaceae* cluster with 672 variants, the three AmpC groups DHA, ACT/MIR and CMY are known to spread on plasmids. Different ACT/MIR variants were mainly isolated from *K. pneumoniae, E. coli, Enterobacter cloacae* and *Enterobacter asburiae*. Their origins remain unclear. The CMY variants were isolated from clinical *C. freundii* and *E. coli* strains but may have originated from *Citrobacter braakii, Klebsiella oxytoca* or *Proteus mirabilis*. The plasmid-born FOX and MOX AmpCs were most often isolated from *Enterobacteriaceae*, but they appear to belong to a clade associated with *Aeromonas*, indicating genus-level transmission of mobile elements. Based on alignment length and percentage identity, it can be assumed that *A. caviae* is the most plausible origin of FOX and MOX (100% identity throughout 342 of 382 amino acids). It is noteworthy that some variants were falsely assigned to the CMY group in the literature, but they were correctly assigned to MOX in the ‘*Aeromonas*’ clade in the present phylogenetic analysis (see Supplementary Table 3 for details). DHA variants were most often isolated from *K. pneumoniae, E. coli* and *Morganella morganii*, the latter of which is considered an origin of DHA. Among ACC variants, the species *Hafnia alvei* may be the most plausible origin (100% identity throughout 345 of 377 amino acids). Obviously, some plasmid-localized variants are spreading among genera and species. Moreover, AmpC variants translocated to plasmids tend to diversify rapidly, as observed in the phylogenetic tree. Most of the few known class C carbapenemases belong to the plasmid-born DHA, ACT and CMY groups. Similarly to those plasmid-born carbapenemases, the activity of the chromosomally encoded ADC-68 is related to an alteration of two loops (R2 and C loops) that usually impedes the entry and binding of the carbapenems^35^, indicating that many AmpCs bear potential hydrolytic activity against carbapenems and that their diversification to carbapenemases might be accelerated by increased selective pressure due to enhanced use of carbapenems.

### Class D

**Figure 6:**
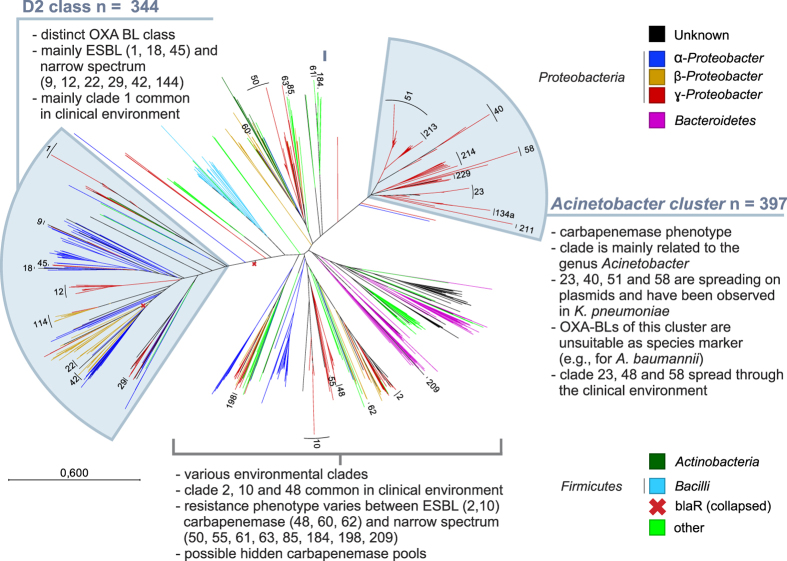
Radial tree representation of 1,843 class D-related variants. A total of 433 blaR-like receptor sequences are indicated but collapsed (red cross); branches are colored according to the organism (class). The tree is clustered for illustrative purposes. Clades are labeled according to their described OXA group. Details are provided in Supplementary Table 7 and in the OXA group assignment.

Phylogenetic analysis of 1,843 class D (OXA BLs)-related proteins demonstrated the insufficient discriminatory power of the current classification, which is based solely on numbering nomenclature that does not reflect the actual diversity of class D. OXA variants encoding carbapenemases and ESBLs are not restricted to *Acinetobacter baumannii*, and a huge number of uncharacterized variants and related proteins were found to be widespread in different phyla, indicating an underestimated pool of class D BLs. In Fig. 6, the OXA clades were named according to the proposed nomenclature (see above). Further details for all 1,843 sequences can be found in Supplementary Table 7.

The radial tree topology revealed a distinct *Acinetobacter*-associated cluster consisting of clades with described carbapenemase variants. Many of these clades are mainly chromosomally localized and are related to the clinically important pathogen *A. baumannii*, particularly OXA clades 23, 40, 51, and 58 and partially 134a (also *A. lwoffii*). These clades most likely resulted from early gene duplications based on the presence of two different OXA variants in some clinical isolates (e.g., OXA-51 and OXA-40 or OXA-51 and OXA-58)^38^. It must be noted that the variants OXA-23, -40, -51 and -58 are currently spreading on plasmids^24^, and therefore transmission to other species can be reasonably assumed. Thus, contrary to the often-made assertion that these variants are species-specific, the OXA variants of this cluster are of rather insufficient discriminatory power. However, OXA clade 213 seems to be primarily associated with *A. calcoaceticus* (human intestinal flora), OXA clade 229 with *A. bereziniae,* and OXA clade 211 with *A. johnsonii*, whereas OXA clade 214 was found in different *Acinetobacter* species such as *A. haemolyticus, A. venetianus, A. baumannii* and *A. gyllenbergii*. To the best of our knowledge, plasmid-born variants have not been reported for these clades^24^.

As indicated by the SSN analysis, OXA-BLs related to clades 1, 12, 22, 42, 114, 45, 9, 18 and 29 form a distant and distinct cluster and were merged to form the D2 class (Fig. 6). The divergent evolutionary origin of the D2 class is supported by its structural protein homology to the extra-cellular sensor domain of BlaR^39^ and YbxI. BlaR is the transmembrane regulator of a two-component system that induces transcription of the *blaZ* gene (a class A BL) in the presence of β-lactam antibiotics in Gram-positive species (e.g., *S. aureus*)^40^. YbxI is an enzyme with latent BL activity that lies between the lowest activity of the narrow-spectrum BLs and the PBPs^41^. In contrast to the Acinetobacter clade, the predominant resistance phenotype of the D2 class is ESBL (groups 1, 18 and 45) or narrow-spectrum activity; thus, it is unlikely that this class contributes to a potential carbapenemase pool. Most OXA variants in the D2 class have been identified in α-*Proteobacteria* and β-*Proteobacteria*; only a few are related to ɣ-*Proteobacteria*. The clades are usually associated with specific genera (for details, see Supplementary Dataset File), indicating that those are naturally occurring intrinsic variants that are most likely all chromosomally located: e.g., OXA clade 114 is associated with *Achromobacter*, OXA clade 22 with *Ralstonia,* OXA clade 12 with *Aeromonas*, OXA clade 29 with *Legionella*, and OXA clade 45 with *Pseudomonas*. OXA clade 1 spreads in different *Enterobacteriaceae* and *P. aeruginosa,* supporting the observation that once BLs translocate on plasmids, they can be easily transmitted to other genera or even orders. The origin of OXA-1-like variants (*Enterobacteriaceae* and *Pseudomonas*) remains unpredictable.

In addition to these two main clusters, other less related clades associated with a multitude of classes, including clinical and environmental species, were identified, and except for a few clades, they were also associated with specific genera. The plasmid born OXA-48-like carbapenemases were found in various *Enterobacteriaceae. K. pneumoniae* carrying OXA-48 variants were responsible for several clinical outbreaks^42^,^43^. The marine bacterium *Shewanella* may represent the origin or a reservoir of OXA-48 variants because various species *of Shewanella* carry different chromosomally located variants with strong homology to OXA-48. The plasmid-localized OXA-2-like ESBLs were found in opportunists that colonize the lungs of patients with cystic fibrosis, such as *P. aeruginosa, Burkholderia cepacia, K. pneumoniae* and *Stenotrophomonas maltophilia,* indicating that transmission between different non-related genera is strongly enhanced by shared habitat. OXA-2-like variants were also identified in environmental samples without species characterization, and subsequent relatives were associated with different genera. Thus, it remains difficult to estimate the origin or route of transmission in pathogens. The plasmid-localized OXA-10-like variants were mainly identified in *P. aeruginosa,* but they were also detected in some different *Enterobacteriaceae*. The origin of these variants might be *Pseudomonas spp.,* but similarly to OXA-2, subsequent relatives were found in environmental samples, supporting an environmental origin.

## Conclusions

Plasmid-encoded and therefore highly transferable BLs related to Gram-negative bacteria that hydrolyze various classes of β-lactam antibiotics currently represent the main challenge to therapy and diagnostics. The number of identified BLs is increasing annually^44^, and emerging new variants often display more distant relationships with clinically known variants, indicating a different ancestry, e.g., the carbapenemase KPC-1 that was first described in 2001 showed no relevant sequence homology to other known class A BLs^45^.

This work clearly shows that BLs are not derived from a single ancestor but independently evolved in different species. This may be explained by the fact that the β-lactams that are in current clinical use, such as penicillins, cephalosporins and carbapenems, are derived from microbial products that have been shaping the bacterial kingdom for millions of years and have obviously provoked the evolution of counter-strategies, such as the generation of BLs that do not necessarily cause fitness loss^46^.

The emergence of new BLs initiated discussion about the role of lateral exchange between soil and clinical resistomes^8^. Recent work supports the idea that horizontal gene transfer of the soil resistome is limited^47^. In contrast, antibiotic-driven selective pressure enhances the horizontal gene transfer of multi-drug resistance plasmids that often bear one or more BLs in pathogenic species^48^.

Although transmission between environmental microorganisms and pathogens rarely occurs, the huge number of unknown and uncharacterized BLs present in a multitude of environmental and pathogenic species represents an unforeseeable reservoir of potentially transferable resistance genes, thus increasing the probability of this phenomenon. Once they find their way into pathogenic species, resistance genes undergo faster evolution and diversification^49^, as observed in the KPC variants, which increased to 20 variants within 15 years due to mutations. A possible route underlying the transformation of environmental BLs into pathogens was hypothesized to occur through *Proteobacteria*, which harbor many opportunistic species^47^. In the present work, known and putative BLs were predominantly found in *Proteobacteria*, supporting this idea. Moreover, class C, which is considered an intrinsic BL class, has spread almost solely in *Proteobacteria*. Currently, some class C variants that successfully translocated to plasmids are now expressed under the control of strong IS-element promoters^50^. Class C BLs have become clinically meaningful because they contribute to the overall β-lactam-resistance phenotype and their presence often complicates microbiological diagnostics by uncommon resistograms and masking effects, which cannot be clearly interpreted by automated resistance testing systems^51^. Substantially more relevant for healthcare are the new carbapenemases that have been increasingly observed in recent years^52^,^53^. The empiric first-line treatments for moderate-to-severe community-acquired infections suggested by most guidelines are penicillin/BL inhibitor combinations or cephalosporins; thus, many ESBLs and all carbapenemase producers are not covered^54^,^55^. Failure in initial treatment of bloodstream infections caused by ESBL-producing *Enterobacteriaceae* correlates with increased mortality^56^, whereas high mortality in carbapenemase-associated infections is caused by ineffective antibiotic therapy^57^. Therefore, early identification of the resistance profiles of culprit pathogens, particularly in blood stream infections, is required to minimize the delay of appropriate treatment and subsequently reduce mortality. However, the general challenge regarding BL detection is that these enzymes are phylogenetically highly heterogeneous with hundreds of clinically relevant variants that cannot be determined by a ‘one-fits-all’ assay. Moreover, molecular approaches provide insufficient coverage of new variants, and adaptations are time-consuming and costly. With more than 1,800 described BL variants and a vast quantity of environmental BLs and relatives representing a potential genetic pool with an elusive impact on the future emergence and diversification of BLs, *in silico* approaches that more adequately reflect the resistome and genetic evolution of BLs should support the development of appropriate molecular diagnostics and may even help to detect or design appropriate broad-spectrum non-β-lactam BL inhibitors, such as avibactam, similar to the successful development of non-nucleoside reverse transcriptase inhibitors. Indeed, the *in silico* design of these compounds in the 1990 s has contributed to impressive progress in HIV therapy^58^.

## Methods

### Data acquisition

All available BL amino acid sequences were retrieved from the NCBI database at http://www.ncbi.nlm.nih.gov/pathogens/beta-lactamase-data-resources/, which has migrated from the Lahey database (www.lahey.org/Studies). Additionally, OXY, OKP and LEN sequences were extracted from the database at the Pasteur Institute (http://bigsdb.web.pasteur.fr/klebsiella/klebsiella.html), and MBL sequences were obtained from LacED (http://www.laced.uni-stuttgart.de) (Supplementary Table 1–4). When an unambiguous destination for a variant was not available, the related accession number was used as the designation (e.g., FEZ ACB74399.1). Because BL nomenclature is often inaccurate or misleading^13^, the integrity of the collected data (e.g., group assignments) was verified by phylogenetic analysis and additional pairwise comparison (data not shown) for all BL variants to avoid any falsification.

### Sequence analysis

Sequence analyses were performed using CLC Mainworkbench 7.0.2 software (Qiagen) by applying a Clustal Omega^59^ algorithm for multiple sequence alignments (Supplementary Dataset File). Subsequently, maximum likelihood phylogeny tree construction was performed with 100 bootstrap replicates, and the WAG^60^ protein substitution model was used. Because the origins or connections between different BLs are unknown, an unrooted radial phylogenetic tree was applied. Detailed high-resolution trees drawn as a circular phylogram including all bootstraps values can be found in the Supplementary Figures S6–S9. Variants without a subgroup were clustered into new subgroups or merged with existing subgroups based on their identity and phylogenetic relationship (visible clustering).

### Amino acid-based sequence similarity network

The SSN of 1,494 amino acid sequences was performed based on BLAST e-values (rather than BLAST scores), which include several corrections^61^. The e-value threshold was defined by plotting each BLAST e-value against each BLAST score (Supplementary Figure 1), and an e-value of 1e-30 was set as the cutoff. The network was created with the ‘BLAST2SimilarityGraph’ plugin for Cytoscape 2.7.

### Motif evaluation

Motifs were generated using all 1,191 retrieved Ser-BL sequences. Groups with similar motifs and comparably low distances (phylogenetic tree construction) were pooled, and their motifs were extended downstream if conserved (Fig. 3). Motif figures were generated with the WebLogo generator^62^.

### Identification of the *in silico* reservoir of β-lactamase

The BL clan (CL0013), as defined by PFAM^25^, was used for sequence-based data extraction. The clan contains the following members: ‘Beta-lactamase’ (PF00144), ‘Beta-lactamase2’ (PF13354), ‘Glutaminase’ (PF04960), ‘Peptidase_S11’ (PF00768), ‘Peptidase_S13’ (PF02113), and ‘Transpeptidase’ (PF00905). The redundancy of some members was corrected by removing double entries based on their accession numbers (shortest sequence removed), resulting in 112,725 unique sequences. The retrieved sequences were annotated based on a BLAST e-value of e^−170^ or less by using the currently described of 1,886 BLs. Therefore, an e-value cutoff of e^−30^ was chosen to denote ‘BL-related proteins’, and an e-value cutoff of e^−170^ to 0 was chosen for annotation into BL groups (e.g., SHV-like, CTX-M-15-group-like, or OXA-10-group-like). In addition, data such as organism, isolation, and protein name and entry were extracted using Strawberry Perl (5.20.3.1) and the BioPerl package. The protein name (extracted using Strawberry Perl) served as annotation guidance for sequences with an e-value greater than e^−170^, and thus the present BL nomenclature was considered. The PERL script can be found in the Supplementary Dataset File. In total, 2,133 out of 9,681 sequences were excluded from sequence analysis because they are short sequences that are 100% identical to longer sequences (subsequences). All sequences used for phylogenetic tree construction are listed in the Supplementary Dataset File, including their accession numbers, organism and corresponding group/subgroup or cluster.

### Adjustment for extracted class D β-lactamase sequence data

BlaR-like receptor units and highly similar proteins, such as YbxI and OXA-BL-variants, are sometimes difficult to define based on sequence data alone because the membrane domain of BlaR is not always correctly included/annotated. Moreover, 3d protein-structure alignment further illustrates the high similarity between the receptor and OXAs (data not shown). Nevertheless, to avoid overestimation or false representation of the extracted class D BL variants, the 2,381 class D BLs were additionally compared against described or known BlaR sequences that were labeled accordingly, and their clade was collapsed if greater than 80% sequence similarity with the extracellular domain was observed.

## Additional Information

**How to cite this article**: Brandt, C. *et al*. *In silico* serine β-lactamases analysis reveals a huge potential resistome in environmental and pathogenic species. *Sci. Rep.* **7**, 43232; doi: 10.1038/srep43232 (2017).

**Publisher's note:** Springer Nature remains neutral with regard to jurisdictional claims in published maps and institutional affiliations.

## Supplementary Material

Supplementary Material

Supplementary Dataset
