# Supplementary material for: In silico serine β-lactamases analysis reveals a huge potential resistome in environmental and pathogenic species
Source: Sci Rep. 2017 Feb 24;7:43232. doi: 10.1038/srep43232 (PMC5324141; doi:10.1038/srep43232)
Supplement: Supplementary Material [file srep43232-s1.pdf]

Supplementary Information

***In silico* serine β-lactamases analysis reveals a huge potential resistome in environmental and pathogenic species**

Christian Brandt [a, b], Sascha D. Braun [b, c], Claudia Stein [a, b], Peter Slickers [b, c], Ralf Ehricht [b, c], Mathias W. Pletz [a, b], Oliwia Makarewicz [a, b,]*

Center for Infectious Diseases and Infection Control, Jena University Hospital, Jena, Germany [a]; InfectoGnostics Research Campus, Jena, Germany [b]; Alere Technologies GmbH, Jena, Germany [c]

MWP and OM contributed equally to this work as Senior Authors.

* Corresponding author. Tel.: +49 3641 932 4227; Fax: +49 3641 932 4652

E-mail address: oliwia.makarewicz@med.uni-jena.de

Data for the supplement was updated at NCBI[1] on the 10th June 2016.

Sequence Similarity Network (SSN)

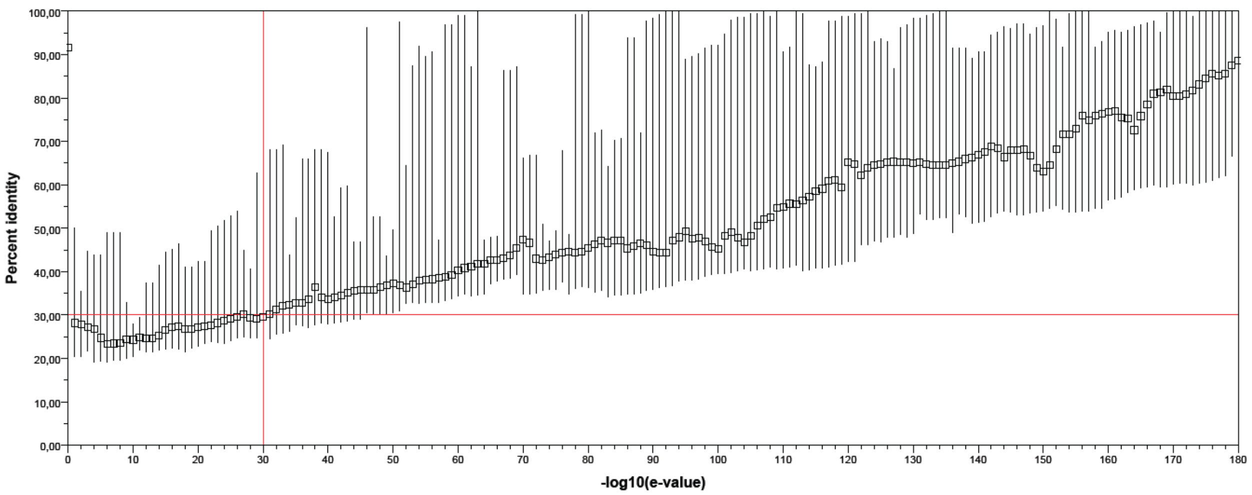

Supplementary Figure S1: Plot of the percent identity against the negative $\log_{10}$(e-value). Circles represent the mean percent identity. Black lines represent quartiles of the percent identity. Red lines show the chosen cut-off of the e-value that was used to calculate the SSN (Figure 1).

Ambler class A

Supplementary Table S1: Overview of the group representatives for the Ambler class A. Members of each group share at least 80 % sequence similarity. For phylogenetic tree see Figure S2.

group	name[accession number_phenotype]
**single**	CTX-M-151[BAP34782.1_ESBL]
**BEL**	BEL-1[AAZ04368.1_ESBL], BEL-2[ACV69996.1_ESBL], BEL-3[ACT09140.1_ESBL]
**CARB-1**	CARB-1[AAK96394.1_carbenicillin], CARB-11[AAG23870.1_carbenicillin], CARB-12[BAA02497.1_carbenicillin], CARB-2[AAA25740.1_carbenicillin], CARB-3[AAB19430.2_carbenicillin], CARB-4[AAC09012.1_carbenicillin], CARB-6[AAD19217.1_carbenicillin], CARB-7[AAM74565.1_carbenicillin], CARB-9[AAP22374.1_carbenicillin]
**CARB-17**	CARB-17[AIL92326.1_carbenicillin], CARB-18[AIL92327.1_carbenicillin], CARB-19[AIL92328.1_carbenicillin], CARB-20[KED43530.1_carbenicillin], CARB-22[BAC61820.1_carbenicillin], CARB-24[DAA64871.1_carbenicillin], CARB-25[DAA64852.1_carbenicillin], CARB-26[DAA64861.1_carbenicillin], CARB-27[DAA64860.1_carbenicillin], CARB-28[DAA64870.1_carbenicillin], CARB-29[DAA64869.1_carbenicillin], CARB-30[DAA64857.1_carbenicillin], CARB-31[DAA64859.1_carbenicillin], CARB-32[DAA64856.1_carbenicillin], CARB-33[DAA64867.1_carbenicillin], CARB-34[DAA64865.1_carbenicillin], CARB-35[DAA64864.1_carbenicillin], CARB-36[DAA64866.1_carbenicillin], CARB-38[DAA64862.1_carbenicillin], CARB-40[DAA64853.1_carbenicillin], CARB-41[DAA64854.1_carbenicillin], CARB-42[DAA64858.1_carbenicillin], CARB-43[DAA64855.1_carbenicillin], CARB-44[KHF12803.1_carbenicillin], CARB-45[KHF09587.1_carbenicillin], CARB-46[KED80204.1_carbenicillin], CARB-47[WP_031841337.1_carbenicillin], CARB-48[WP_025577435.1_carbenicillin], VHH-1[AAF23817.1_carbenicillin], VHW-1[AAF23816.1_carbenicillin]
**CARB-5**	CARB-10[ACJ61335.1_ESBL], CARB-14[AFI72872.1_carbenicillin], CARB-16[CCW43444.1_carbenicillin], CARB-5[AAF61417.1_carbenicillin], CARB-8[AAO59455.1_carbenicillin]
**CTX-M-14**	CTX-M-102[ADY02546.1_ESBL], CTX-M-104[ADY02555.1_ESBL], CTX-M-105[ADY02554.1_ESBL], CTX-M-110[AEM44648.1_ESBL], CTX-M-111[AEM44649.1_ESBL], CTX-M-112[AEM44652.1_ESBL], CTX-M-113[AEM44653.1_ESBL], CTX-M-121[AFA51699.1_ESBL], CTX-M-122[AFA51700.1_ESBL], CTX-M-125[AFO69261.1_ESBL], CTX-M-126[BAL72196.1_ESBL], CTX-M-129[AFJ59956.1_ESBL], CTX-M-13[AAF72531.1_ESBL], CTX-M-130[AFJ59957.1_ESBL], CTX-M-134[AFX60298.1_ESBL], CTX-M-137[BAO37256.1_ESBL], CTX-M-14[AAF72530.1_ESBL], CTX-M-147[AHA80961.1_ESBL], CTX-M-148[AHX39589.1_ESBL], CTX-M-159[BAP18874.1_ESBL], CTX-M-16[AAK32961.1_ESBL], CTX-M-161[AJU57235.1_ESBL], CTX-M-168[ALJ94049.1_ESBL], CTX-M-17[AAK71471.1_ESBL], CTX-M-174[ALM96713.1_ESBL], CTX-M-19[AAK55534.1_ESBL], CTX-M-21[CAD08929.1_ESBL], CTX-M-24[AAN38836.1_ESBL], CTX-M-27[AAO61597.1_ESBL], CTX-M-38[AAV70602.1_ESBL], CTX-M-46[AAV97956.1_ESBL], CTX-M-47[AAV97952.1_ESBL], CTX-M-48[AAV97953.1_ESBL], CTX-M-49[AAV97954.1_ESBL], CTX-M-50[AAV97955.1_ESBL], CTX-M-51[ABA62022.1_ESBL],

[1] http://www.ncbi.nlm.nih.gov/pathogens/submit_beta_lactamase/

	CTX-M-65[ABN69105.1_ESBL], CTX-M-67[ABS90365.1_ESBL], CTX-M-73[ABC69156.1_ESBL], CTX-M-81[ABV57756.3_ESBL], CTX-M-83[ACI29345.1_ESBL], CTX-M-84[ACI29346.1_ESBL], CTX-M-85[ACI29347.1_ESBL], CTX-M-86[ACI29348.1_ESBL], CTX-M-87[ACB41777.1_ESBL], CTX-M-9[AAF05311.2_ESBL], CTX-M-90[ACQ99521.1_ESBL], CTX-M-93[ADN26580.1_ESBL], CTX-M-98[ADO17948.1_ESBL], CTX-M-99[ADL27532.1_ESBL]
**CTX-M-15**	CTX-M-1[CAA63262.1_ESBL], CTX-M-10[AAF65843.1_ESBL], CTX-M-101[ADY02545.1_ESBL], CTX-M-103[CDG50843.1_ESBL], CTX-M-114[ACU00153.1_ESBL], CTX-M-116[AEI70324.1_ESBL], CTX-M-117[AET99223.1_ESBL], CTX-M-12[AAG34108.1_ESBL], CTX-M-123[AFA51701.1_ESBL], CTX-M-132[AFQ94051.1_ESBL], CTX-M-136[AGG08693.1_ESBL], CTX-M-139[AFY98865.1_ESBL], CTX-M-142[AGW25368.1_ESBL], CTX-M-144[AHX39588.1_ESBL], CTX-M-15[AAL02127.1_ESBL], CTX-M-150[AHF20911.1_ESBL], CTX-M-155[AIS67611.1_ESBL], CTX-M-156[AIS67612.1_ESBL], CTX-M-157[AIS67613.1_ESBL], CTX-M-158[AIT97310.1_ESBL], CTX-M-162[AKO63213.1_ESBL], CTX-M-163[AKO63214.1_ESBL], CTX-M-164[AKR53959.1_ESBL], CTX-M-166[CFW94147.1_ESBL], CTX-M-167[ALJ77593.1_ESBL], CTX-M-169[ALL29307.1_ESBL], CTX-M-170[ALK03059.1_ESBL], CTX-M-172[AKS25179.1_ESBL], CTX-M-173[ALM96711.1_ESBL], CTX-M-175[ALM96714.1_ESBL], CTX-M-176[ALM96715.1_ESBL], CTX-M-177[ALM96716.1_ESBL], CTX-M-179[AMJ17432.1_ESBL], CTX-M-180[AMY95406.1_unknown], CTX-M-181[AMY95407.1_unknown], CTX-M-182[AMY95408.1_unknown], CTX-M-22[AAL86924.1_ESBL], CTX-M-23[AAL99990.1_ESBL], CTX-M-28[CAD70280.1_ESBL], CTX-M-29[AAP22736.1_ESBL], CTX-M-3[BAD34467.1_ESBL], CTX-M-30[AAP43508.1_ESBL], CTX-M-32[CAD89606.1_ESBL], CTX-M-33[AAO88912.1_ESBL], CTX-M-34[AAR99493.1_ESBL], CTX-M-36[BAD16611.1_ESBL], CTX-M-37[AAT70415.1_ESBL], CTX-M-42[AAY84742.1_ESBL], CTX-M-52[ABB17185.1_ESBL], CTX-M-53[ABB72225.1_ESBL], CTX-M-54[ABC18328.3_ESBL], CTX-M-55[ABI34705.1_ESBL], CTX-M-58[ABM97538.1_ESBL], CTX-M-60[CAL80726.1_ESBL], CTX-M-61[ABN09669.1_ESBL], CTX-M-64[BAF63422.1_ESBL], CTX-M-66[ABQ45409.1_ESBL], CTX-M-68[ABV81082.1_ESBL], CTX-M-69[ABY91281.1_ESBL], CTX-M-70[ADA62555.1_ESBL], CTX-M-71[ACV92002.1_ESBL], CTX-M-72[AAV97957.1_ESBL], CTX-M-79[ABO09821.1_ESBL], CTX-M-80[ABW86620.2_ESBL], CTX-M-82[ABB59946.1_ESBL], CTX-M-88[ACP18863.1_ESBL], CTX-M-96[CAG28417.1_ESBL], KLUC-1[AAK08976.1_ESBL], KLUC-2[ABM73648.1_ESBL]
**CTX-M-2**<br>**CTX-M-8**	CTX-M-100[CBW46935.1_ESBL], CTX-M-115[AIL54055.1_ESBL], CTX-M-124[AFH88134.1_ESBL], CTX-M-131[AEW46676.3_ESBL], CTX-M-141[AGN11769.1_ESBL], CTX-M-152[AHY20039.1_ESBL], CTX-M-160[AJO16046.1_ESBL], CTX-M-165[AKR53960.1_ESBL], CTX-M-2[CAA63263.1_ESBL], CTX-M-20[CAC95175.1_ESBL], CTX-M-25[AAM70498.1_ESBL], CTX-M-26[AAN75444.1_ESBL], CTX-M-31[CAD99181.1_ESBL], CTX-M-35[BAD83773.1_ESBL], CTX-M-39[AAX54694.1_ESBL], CTX-M-4[CAA74573.1_ESBL], CTX-M-40[AAV28215.2_ESBL], CTX-M-41[AAY43008.1_ESBL], CTX-M-43[AAZ14955.1_ESBL], CTX-M-44[BAA07082.1_ESBL], CTX-M-5[AAC32890.1_ESBL], CTX-M-56[ABN48311.1_ESBL], CTX-M-59[ABD73290.1_ESBL], CTX-M-6[CAA06311.1_ESBL], CTX-M-63[BAD90119.1_ESBL], CTX-M-7[CAA06312.1_ESBL], CTX-M-74[ACS32293.1_ESBL], CTX-M-75[ACS32294.1_ESBL], CTX-M-76[CAQ42486.1_ESBL], CTX-M-77[CAQ42480.1_ESBL], CTX-M-78[CAQ42481.2_ESBL], CTX-M-8[AAF04388.1_ESBL], CTX-M-89[ACR56321.1_ESBL], CTX-M-91[ACX34099.1_ESBL], CTX-M-92[ACY74743.1_ESBL], CTX-M-94[ADK11041.1_ESBL], CTX-M-95[CBL80615.1_ESBL], CTX-M-97[ADM08178.1_ESBL]
**GES**	GES-1[AAF27723.1_ESBL], GES-10[ACS73598.1_unknown], GES-11[ACS44714.1_ESBL], GES-12[CBG22732.1_unknown], GES-13[ACZ54536.1_unknown], GES-14[ADC91899.1_carbapenemase], GES-15[ACZ98826.1_carbapenemase], GES-16[ADJ94120.1_carbapenemase], GES-17[ADZ48685.1_unknown], GES-18[AEX59144.1_carbapenemase], GES-19[AEZ05107.1_unknown], GES-2[AAK58421.1_ESBL], GES-20[AEZ05108.1_carbapenemase], GES-21[AFK80745.1_carbapenemase], GES-22[AFU25739.1_unknown], GES-23[AGT20529.1_ESBL], GES-24[BAP75641.1_carbapenemase], GES-25[ALE31310.1_unknown], GES-26[AJP67510.1_unknown], GES-27[ALN67755.1_unknown], GES-28[ALC74967.1_unknown], GES-29[ALM96712.1_unknown], GES-3[BAD06399.1_ESBL], GES-30[BAU51851.1_unknown], GES-31[AMY16492.1_unknown], GES-4[BAD08689.1_carbapenemase], GES-5[AAR97270.1_carbapenemase], GES-6[AAR97271.1_carbapenemase], GES-7[AAF28763.2_unknown], GES-8[AAK18183.1_ESBL], GES-9[AAY43207.1_unknown]
**IMI**	IMI-1[AAA93461.1_carbapenemase], IMI-2[ABA00479.1_carbapenemase], IMI-3[ACX71212.1_carbapenemase], IMI-4[AHE78014.1_carbapenemase], IMI-6[ALD82364.1_carbapenemase], IMI-7[AIS19858.1_carbapenemase], IMI-8[AIY69088.1_carbapenemase], IMI-9[KSX64667.1_carbapenemase], NMC-A[CAA79967.1_carbapenemase]
**KPC**	KPC-10[ACS35345.1_carbapenemase], KPC-11[ADH95186.1_carbapenemase], KPC-12[ADZ75467.1_carbapenemase], KPC-13[AEA73284.1_carbapenemase], KPC-14[AFV48348.1_carbapenemase], KPC-15[AGF70638.1_carbapenemase], KPC-16[AGJ01153.1_carbapenemase], KPC-17[AGJ01154.1_carbapenemase], KPC-18[AKO63215.1_carbapenemase], KPC-19[AIH07017.1_carbapenemase], KPC-2[AAK70220.1_carbapenemase], KPC-21[CEF57509.1_carbapenemase], KPC-22[AIX87991.1_carbapenemase], KPC-24[AKQ06274.1_carbapenemase], KPC-3[AAL05630.1_carbapenemase], KPC-4[ACA34343.1_carbapenemase], KPC-5[ABY91240.1_carbapenemase], KPC-6[ACB71165.1_carbapenemase], KPC-7[ACE62798.1_carbapenemase], KPC-8[ACI95258.1_carbapenemase]
**LEN**	LEN-1[X04515_narrow], LEN-10[AJ635419_narrow], LEN-11[AJ635417_narrow], LEN-12[AJ635406_narrow], LEN-13[AJ635403_narrow], LEN-14[AY265889_narrow], LEN-15[AF452105_narrow], LEN-16[AY743416_narrow], LEN-18[AM850908_narrow], LEN-19[AM850909_narrow], LEN-2[Pasteur DB_narrow], LEN-20[AM850910_narrow], LEN-21[AM850911_narrow], LEN-22[AM850912_narrow], LEN-23[AM850913_narrow], LEN-24[AM850914_narrow], LEN-25[AY037780_narrow], LEN-26[Pasteur DB_narrow], LEN-3[AY130286_narrow], LEN-4[AY130287_narrow], LEN-5[AY633109_narrow], LEN-6[AY265890_narrow], LEN-7[AJ635425_narrow], LEN-8[AJ635424_narrow], LEN-9[AJ635405_narrow]
**OKP**	OKP-A-1[Pasteur DB_narrow], OKP-A-10[Pasteur DB_narrow], OKP-A-11[Pasteur DB_narrow], OKP-A-12[Pasteur DB_narrow], OKP-A-13[Pasteur DB_narrow], OKP-A-14[Pasteur DB_narrow], OKP-A-15[Pasteur DB_narrow], OKP-A-16[Pasteur DB_narrow], OKP-A-2[Pasteur DB_narrow], OKP-A-3[Pasteur DB_narrow], OKP-A-4[Pasteur DB_narrow], OKP-A-5[Pasteur DB_narrow], OKP-A-6[Pasteur DB_narrow], OKP-A-7[Pasteur DB_narrow], OKP-A-8[Pasteur DB_narrow], OKP-A-9[Pasteur DB_narrow], OKP-B-1[Pasteur DB_narrow], OKP-B-10[Pasteur DB_narrow], OKP-B-11[Pasteur DB_narrow], OKP-B-12[Pasteur DB_narrow], OKP-B-13[Pasteur DB_narrow], OKP-B-14[Pasteur DB_narrow], OKP-B-17[Pasteur DB_narrow], OKP-B-18[Pasteur DB_narrow], OKP-B-19[Pasteur DB_narrow], OKP-B-2[Pasteur DB_narrow], OKP-B-20[Pasteur DB_narrow], OKP-B-3[Pasteur DB_narrow], OKP-B-4[Pasteur DB_narrow], OKP-B-5[Pasteur DB_narrow], OKP-B-6[Pasteur DB_narrow], OKP-B-7[Pasteur DB_narrow], OKP-B-8[Pasteur DB_narrow], OKP-B-9[Pasteur DB_narrow]
**OXY**	OXY_1-1[Pasteur DB_ESBL], OXY_1-2[Pasteur DB_ESBL], OXY_1-3[Pasteur DB_ESBL], OXY_1-4[Pasteur DB_ESBL], OXY_1-5[Pasteur DB_ESBL], OXY_1-6[Pasteur DB_ESBL], OXY_1-8[Pasteur DB_ESBL], OXY_1-9[Pasteur DB_ESBL], OXY_2-1[Pasteur DB_ESBL], OXY_2-10[Pasteur DB_ESBL], OXY_2-11[Pasteur DB_ESBL], OXY_2-12[Pasteur DB_ESBL], OXY_2-13[Pasteur DB_ESBL], OXY_2-14[Pasteur DB_ESBL], OXY_2-15[Pasteur DB_ESBL], OXY_2-16[Pasteur DB_ESBL], OXY_2-17[Pasteur DB_ESBL], OXY_2-18[Pasteur DB_ESBL], OXY_2-19[Pasteur DB_ESBL], OXY_2-2[Pasteur DB_ESBL], OXY_2-20[Pasteur DB_ESBL], OXY_2-3[Pasteur DB_ESBL], OXY_2-4[Pasteur DB_ESBL], OXY_2-6[Pasteur DB_ESBL], OXY_2-7[Pasteur DB_ESBL], OXY_2-8[Pasteur DB_ESBL], OXY_2-9[Pasteur DB_ESBL], OXY_3-1[Pasteur DB_ESBL], OXY_4-1[Pasteur DB_ESBL], OXY_5-1[Pasteur DB_ESBL], OXY_5-2[Pasteur DB_ESBL], OXY_5-3[Pasteur DB_ESBL], OXY_6-1[Pasteur DB_ESBL], OXY_6-2[Pasteur DB_ESBL], OXY_6-3[Pasteur DB_ESBL],

	OXY_6-4[Pasteur DB_ESBL], OXY_7-1[Pasteur DB_ESBL]
**PER**	PER-1[CAA79968.1_ESBL], PER-2[CAA63714.1_ESBL], PER-3[AAU89132.1_ESBL], PER-4[ACE77058.1_ESBL], PER-5[ACN22483.1_ESBL], PER-6[ADD80743.1_ESBL], PER-7[AEI54993.1_ESBL], PER-8[BAP39824.1_ESBL], PER-9[BAU25894.1_ESBL]
**SHV**	SHV-1[AAD37412.1_narrow], SHV-100[CAQ03505.1_unknown], SHV-101[ABV72593.1_unknown], SHV-102[ABS72342.1_ESBL], SHV-103[ABS72351.1_unknown], SHV-104[ABX71158.1_ESBL], SHV-105[ACI22621.1_ESBL], SHV-106[CAP58290.1_ESBL], SHV-107[CAP58291.1_narrow], SHV-108[CAP58292.1_unknown], SHV-109[ACM04459.1_narrow], SHV-11[CAA66729.1_narrow], SHV-110[AEK48094.1_unknown], SHV-111[BAF95849.1_unknown], SHV-119[AIG51284.1_unknown], SHV-120[AEG79634.1_unknown], SHV-121[ACV53157.1_unknown], SHV-128[ADE58494.1_ESBL], SHV-129[ADE08533.1_ESBL], SHV-13[AAD43815.1_ESBL], SHV-132[ADU15837.1_unknown], SHV-133[BAI94487.1_unknown], SHV-134[ADM25824.1_ESBL], SHV-135[ADR66517.1_unknown], SHV-137[AEI83430.1_unknown], SHV-14[AAG17550.1_narrow], SHV-141[AFC60795.1_narrow], SHV-142[AEX99752.1_unknown], SHV-143[AFQ32277.1_unknown], SHV-144[AFK93491.1_unknown], SHV-145[AFN88952.1_unknown], SHV-146[AKK23731.1_unknown], SHV-147[AFQ23953.1_unknown], SHV-148[AFQ23954.1_ESBL], SHV-149[AFQ23955.1_unknown], SHV-15[CAB37325.2_ESBL], SHV-150[AFQ23956.1_unknown], SHV-151[AFQ23957.1_unknown], SHV-152[AFQ23958.1_unknown], SHV-153[AFQ23959.1_unknown], SHV-154[AFQ23960.1_unknown], SHV-155[AFQ23961.1_unknown], SHV-156[AFQ23962.1_unknown], SHV-157[AFQ23963.1_unknown], SHV-158[AFQ23964.1_unknown], SHV-159[AFQ23965.1_unknown], SHV-16[AAC98092.2_ESBL], SHV-160[AFQ23966.1_unknown], SHV-161[AFQ23967.1_unknown], SHV-162[AFQ23968.1_unknown], SHV-163[AFQ23969.1_unknown], SHV-164[CCK86744.1_narrow], SHV-165[AFQ23970.1_unknown], SHV-168[AFW16978.1_unknown], SHV-172[AHA80958.1_unknown], SHV-173[AHA80959.1_unknown], SHV-178[AHA80963.1_unknown], SHV-179[AHA80962.1_unknown], SHV-18[AAF64386.1_ESBL], SHV-180[AJO16041.1_unknown], SHV-182[AJO16042.1_unknown], SHV-183[CDN33427.1_ESBL], SHV-185[AIS67768.1_unknown], SHV-186[AIS67769.1_unknown], SHV-187[CEA29750.1_unknown], SHV-188[CEA29751.1_unknown], SHV-189[AJO16047.1_unknown], SHV-190[AKK23732.1_unknown], SHV-191[AKK23733.1_unknown], SHV-193[ALI16958.1_unknown], SHV-1b-b[AAZ98828.1_unknown], SHV-2[AAD37413.1_ESBL], SHV-24[BAA84973.1_ESBL], SHV-25[AAF37209.2_narrow], SHV-26[AAF36719.1_narrow], SHV-27[AAG01039.1_ESBL], SHV-28[ACA09611.1_unknown], SHV-29[AAG49894.1_unknown], SHV-2A[CAA66730.1_ESBL], SHV-3[ANA06389.1_ESBL], SHV-30[AAT75225.1_ESBL], SHV-31[AAP33454.2_ESBL], SHV-32[AAK69828.1_narrow], SHV-33[AAK69829.1_narrow], SHV-34[AAK64187.1_ESBL], SHV-35[AAL68926.1_unknown], SHV-36[AAL82592.1_unknown], SHV-37[AAL82593.1_unknown], SHV-38[AAL79576.1_ESBL], SHV-40[AAN04882.1_ESBL], SHV-41[AAN04883.1_ESBL], SHV-42[AAN04884.1_ESBL], SHV-43[AAL40899.1_narrow], SHV-44[AAP82228.1_narrow], SHV-45[AAN39364.1_ESBL], SHV-46[AAO53445.1_ESBL], SHV-48[AAP03063.1_narrow], SHV-49[AAS98184.1_narrow], SHV-5[CAA39164.1_ESBL], SHV-50[AAP41108.1_unknown], SHV-51[AAP41944.1_unknown], SHV-52[AEJ08681.1_unknown], SHV-55[AAY51674.1_ESBL], SHV-56[ACB73258.1_narrow], SHV-57[AAO66446.1_ESBL], SHV-59[AAV66328.1_unknown], SHV-60[CAI30649.2_narrow], SHV-61[CAI30650.2_narrow], SHV-62[CAI30651.2_narrow], SHV-63[ABY56290.1_unknown], SHV-64[ABA06586.1_ESBL], SHV-65[ABA06587.1_unknown], SHV-66[ABA06588.1_ESBL], SHV-67[ABA06589.1_unknown], SHV-69[ABA06590.1_unknown], SHV-7[AAA87176.1_ESBL], SHV-70[AAY42633.1_ESBL], SHV-71[CAJ47126.2_narrow], SHV-72[CAJ47127.2_narrow], SHV-73[CAJ47128.2_narrow], SHV-74[CAJ47129.2_narrow], SHV-75[CAJ47130.2_narrow], SHV-76[CAJ47131.2_narrow], SHV-77[CAJ47132.2_narrow], SHV-78[CAJ47133.2_narrow], SHV-79[CAJ47134.2_narrow], SHV-8[AAB51384.1_ESBL], SHV-80[CAJ47135.2_narrow], SHV-81[CAJ47136.2_narrow], SHV-82[CAJ47137.2_narrow], SHV-85[ABC54571.1_narrow], SHV-86[ABC58727.1_ESBL], SHV-89[ABA60809.1_narrow], SHV-9[AAB37395.2_unknown], SHV-92[ABH04327.1_unknown], SHV-93[ABN49110.1_unknown], SHV-94[ABN49111.1_unknown], SHV-95[ABN49113.1_unknown], SHV-96[ABN49112.1_unknown], SHV-97[ABN49114.1_unknown], SHV-98[CAQ03503.1_ESBL], SHV-99[CAQ03504.1_ESBL]
**SME**	SME-1[CAA82281.1_carbapenemase], SME-2[AAG29813.1_carbapenemase], SME-3[AAS92558.1_carbapenemase], SME-4[AHA49908.1_carbapenemase], SME-5[AHV85514.1_carbapenemase]
**TEM**	TEM-1[AAB59737.1_narrow], TEM-10[AAC72362.1_ESBL], TEM-101[AAM18924.1_ESBL], TEM-102[AAK82652.1_ESBL], TEM-104[AAM61952.1_unknown], TEM-105[AAM61953.1_unknown], TEM-106[AAM52207.1_ESBL], TEM-107[AAM52215.1_ESBL], TEM-108[AAM28884.1_unknown], TEM-109[AAT46413.1_ESBL], TEM-11[AAW66604.1_ESBL], TEM-110[AAL68923.1_narrow], TEM-111[AAL77062.1_unknown], TEM-112[AAS89982.1_ESBL], TEM-113[AAS89983.1_ESBL], TEM-114[AAS89984.1_ESBL], TEM-115[AAN04881.1_ESBL], TEM-116[AAB39956.1_narrow], TEM-12[AAA25053.1_ESBL], TEM-120[AAO85882.1_ESBL], TEM-121[AAQ01671.1_ESBL], TEM-122[AAQ98890.1_narrow], TEM-123[AAQ93490.1_ESBL], TEM-124[AAQ93491.1_ESBL], TEM-125[AAT46414.1_ESBL], TEM-126[AAT45742.1_ESBL], TEM-127[AAR89358.1_narrow], TEM-128[AAR89359.1_narrow], TEM-129[CAG34105.1_ESBL], TEM-130[CAI29263.1_ESBL], TEM-131[AAR10958.1_ESBL], TEM-132[AAR84298.1_ESBL], TEM-133[AAS19171.1_ESBL], TEM-134[AAS79107.1_ESBL], TEM-135[CAG25427.1_narrow], TEM-136[AAV83795.1_ESBL], TEM-137[CAL08007.1_ESBL], TEM-138[AAW47922.1_ESBL], TEM-139[AAZ23494.1_ESBL], TEM-141[AAX56615.1_narrow], TEM-142[ABD60314.1_unknown], TEM-143[AAY85632.1_ESBL], TEM-144[CAJ17558.1_ESBL], TEM-145[AAZ14083.2_narrow], TEM-146[AAZ14084.2_unknown], TEM-147[ABB84515.1_ESBL], TEM-148[CAJ32372.1_unknown], TEM-149[ABC96711.1_ESBL], TEM-15[CAO98721.1_ESBL], TEM-150[CAJ66089.1_unknown], TEM-151[ABI74448.1_ESBL], TEM-152[ABI74447.1_ESBL], TEM-153[AGA83484.1_ESBL], TEM-154[ACO07310.1_ESBL], TEM-155[ABG77582.1_ESBL], TEM-156[CAQ00120.1_unknown], TEM-157[ABI81768.1_ESBL], TEM-158[ABQ00181.1_ESBL], TEM-159[ABM54869.1_narrow], TEM-16[CAA46346.1_ESBL], TEM-160[ABM54870.1_unknown], TEM-162[ABO64442.1_unknown], TEM-163[ACF32746.1_narrow], TEM-164[ABX71157.1_ESBL], TEM-166[ACI25375.1_unknown], TEM-167[ACJ04051.1_ESBL], TEM-168[ACR22829.1_ESBL], TEM-169[ACP18864.1_ESBL], TEM-17[CAA74912.2_ESBL], TEM-171[ADA79630.1_unknown], TEM-176[ADB90239.1_unknown], TEM-177[CBJ06718.1_ESBL], TEM-178[CAA65888.1_ESBL], TEM-181[AAA72077.1_unknown], TEM-182[ADP20705.1_narrow], TEM-183[ADR71220.1_narrow], TEM-184[CCA61905.1_ESBL], TEM-185[AEG64812.1_narrow], TEM-186[AET99222.1_narrow], TEM-187[ADM61585.1_ESBL], TEM-188[AEL17198.1_ESBL], TEM-189[AEL79515.1_unknown], TEM-19[AFN21551.1_ESBL], TEM-190[AEL88240.1_unknown], TEM-193[AFC75523.1_unknown], TEM-194[AFC75524.1_unknown], TEM-195[AFC75525.1_unknown], TEM-197[AEK48085.1_ESBL], TEM-198[BAL68178.1_unknown], TEM-2[CAA38429.1_narrow], TEM-20[CAA76793.1_ESBL], TEM-201[AFS44742.1_unknown], TEM-205[AGZ20205.1_unknown], TEM-206[AGK82336.1_narrow], TEM-207[AGK40892.1_ESBL], TEM-208[AGL39384.1_unknown], TEM-209[AGW25367.1_unknown], TEM-21[CAA76794.1_ESBL], TEM-210[AIF78090.1_unknown], TEM-211[AHA80960.1_ESBL], TEM-212[AHA49909.1_unknown], TEM-213[AHA58207.1_unknown], TEM-214[AJO16044.1_unknown], TEM-215[AJO16045.1_unknown], TEM-216[AHJ78622.1_unknown], TEM-217[CDN33426.1_unknown], TEM-219[AIS39742.1_unknown], TEM-22[CAA76795.1_ESBL], TEM-220[AIW68620.1_unknown], TEM-224[AMD11804.1_unknown], TEM-24[CAA46345.1_ESBL], TEM-26[KLG19745.1_ESBL], TEM-28[AAC32891.1_ESBL], TEM-29[CAA76796.1_ESBL], TEM-3[CAA45828.1_ESBL], TEM-30[CAD24670.1_narrow], TEM-32[CTD69961.1_narrow],

	TEM-33[ADL13944.1_narrow], TEM-34[AGE11905.1_narrow], TEM-35[AKA60778.1_narrow], TEM-4[CDR98216.1_ESBL], TEM-40[CBX53726.1_narrow], TEM-43[AAC32889.2_ESBL], TEM-45[CAA64682.1_narrow], TEM-47[CAA71322.1_ESBL], TEM-48[CAA71323.1_ESBL], TEM-49[CAA71324.1_ESBL], TEM-52[CAA73933.1_ESBL], TEM-53[AAD22538.1_ESBL], TEM-54[AAD22539.1_narrow], TEM-55[ABB97007.1_narrow], TEM-57[ACJ43254.1_narrow], TEM-6[CAA41038.1_ESBL], TEM-60[AAC05975.1_ESBL], TEM-63[AAK17194.1_ESBL], TEM-67[AAD33116.2_narrow], TEM-68[CAB92324.1_ESBL], TEM-70[AAF01046.1_unknown], TEM-71[AAL03985.1_ESBL], TEM-72[AAF19151.1_ESBL], TEM-76[AAF05613.1_narrow], TEM-77[AAF05614.1_narrow], TEM-78[AAF05612.1_narrow], TEM-79[AAF05611.1_narrow], TEM-8[CAA46344.1_ESBL], TEM-80[AAM15527.1_narrow], TEM-81[AAL29433.1_narrow], TEM-82[AAL29434.1_narrow], TEM-83[AAL29435.1_narrow], TEM-84[AAL29436.1_narrow], TEM-85[CAC43229.1_ESBL], TEM-86[CAC43230.1_ESBL], TEM-87[AAG44570.1_ESBL], TEM-88[AAK14792.1_ESBL], TEM-90[AAK30619.1_narrow], TEM-91[BAB16308.1_ESBL], TEM-92[AAF66653.1_ESBL], TEM-93[CAC85660.1_ESBL], TEM-94[CAC85661.1_ESBL], TEM-95[CAC67290.1_narrow], TEM-96[AAM22276.1_unknown], TEM-97[AAK85244.1_unknown], TEM-98[AAK85245.1_unknown], TEM-99[AAK85243.1_unknown]
**VEB**	VEB-1[AAD01435.1_ESBL], VEB-10[AJH76942.1_ESBL], VEB-11[AKO63208.1_ESBL], VEB-12[AKO63209.1_ESBL], VEB-13[ALB25884.1_ESBL], VEB-14[AMJ32273.1_ESBL], VEB-15[ALB25886.1_ESBL], VEB-16[ALB25887.1_ESBL], VEB-17[AMJ32272.1_ESBL], VEB-2[AAK29174.1_ESBL], VEB-3[AAS48620.1_ESBL], VEB-4[ABM54868.1_ESBL], VEB-5[ABN80430.1_ESBL], VEB-6[ACA34904.1_ESBL], VEB-7[ACO56763.1_ESBL], VEB-8[AGH33739.1_ESBL], VEB-9[AAK14293.1_ESBL]

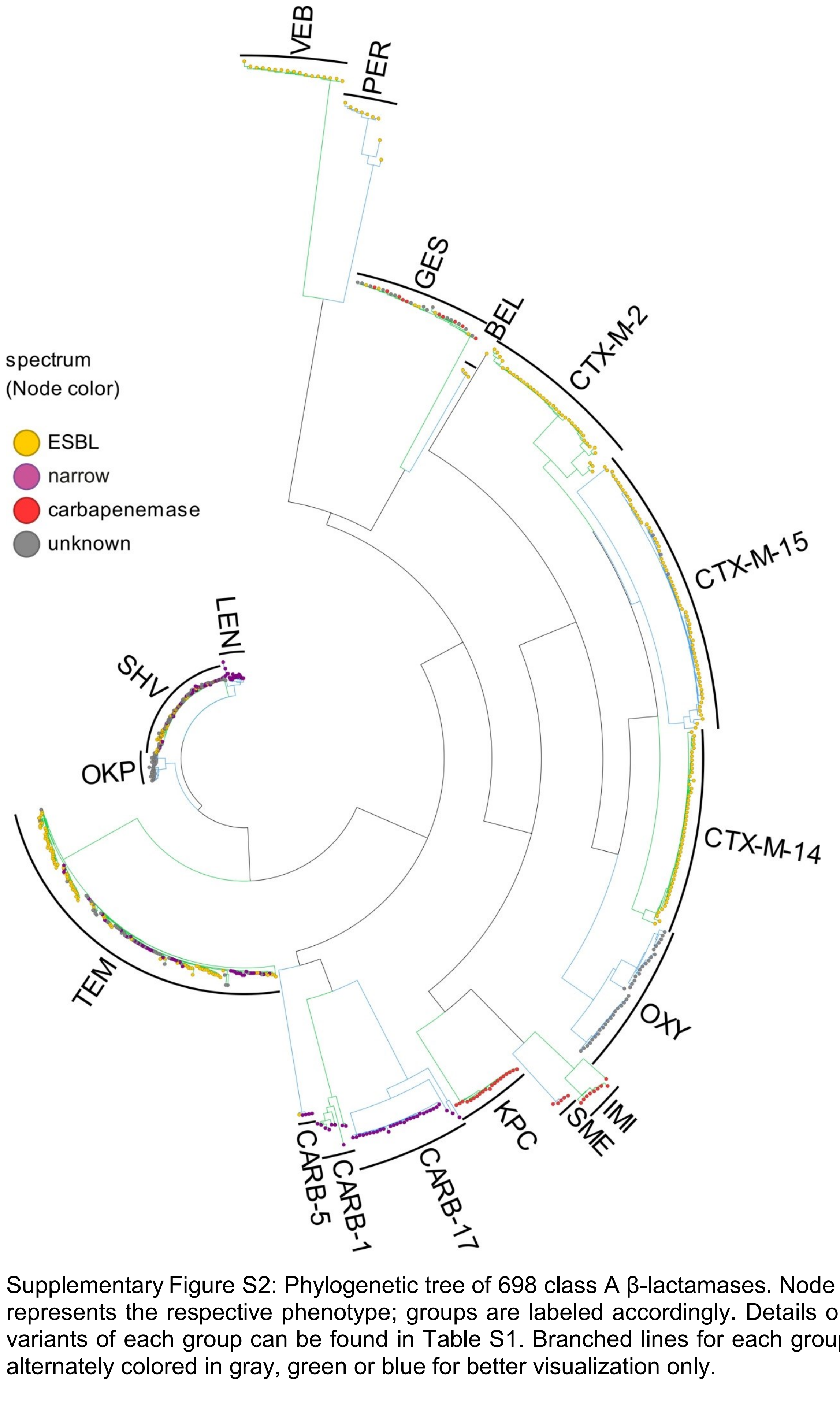

Supplementary Figure S2: Phylogenetic tree of 698 class A β-lactamases. Node color represents the respective phenotype; groups are labeled accordingly. Details on the variants of each group can be found in Table S1. Branched lines for each group are alternately colored in gray, green or blue for better visualization only.

Ambler class B

Supplementary Table S2: Overview of the group representatives for the Ambler class B groups. Members of each group share at least 70 % sequence similarity, except for the AIM cluster. All known class B β-lactamases are carbapenemases. For phylogenetic tree see Figure S3.

class	group	name[accession number]
B1	single	KHM-1[BAF91108.1]
	GIM	GIM-1[CAF05908.1], GIM-2[AIY26289.1]
	IMP	IMP-1[AAB30289.1], IMP-10[BAB72069.1], IMP-11[BAB72072.1], IMP-12[CAD12765.1], IMP-13[CAD80251.1], IMP-14[AAT49068.1], IMP-15[AAT49070.1], IMP-16[CAE48334.1], IMP-17[CAD55935.1], IMP-18[AAV49162.2], IMP-19[ABM67078.1], IMP-2[CAB94707.1], IMP-20[BAD81061.1], IMP-21[BAD89802.1], IMP-22[ABC88434.1], IMP-23[ABD85195.1], IMP-24[ABM68358.1], IMP-25[ACB41775.1], IMP-26[ACY01749.1], IMP-27[AEH41427.1], IMP-28[AFG73659.1], IMP-29[ADT63777.1], IMP-3[BAA77393.1], IMP-30[ABF70513.1], IMP-31[AGS82587.1], IMP-32[AFR33816.1], IMP-33[AEU17778.1], IMP-34[BAM37538.1], IMP-35[AFO59566.1], IMP-37[AFP97028.1], IMP-38[AEN75249.1], IMP-4[AAK13078.1], IMP-40[BAM62794.1], IMP-41[BAM62795.1], IMP-42[BAM62793.1], IMP-43[BAM98935.1], IMP-44[BAM98942.1], IMP-45[AIA58910.1], IMP-48[AIT76110.1], IMP-49[AKO63210.1], IMP-5[AAK27847.1], IMP-51[BAQ56016.1], IMP-52[BAR80870.1], IMP-53[ADI49230.1], IMP-54[ALN95135.1], IMP-55[ALT07696.1], IMP-56[ALU87096.1], IMP-58[AMJ16609.1], IMP-6[BAB15941.1], IMP-7[AAK12087.1], IMP-8[AJO16039.1], IMP-9[AGL46523.1]
	IND	IND-1[AAD20273.1], IND-10[ADA13241.1], IND-11[ADK25050.1], IND-12[ADK25051.1], IND-14[ADK38716.1], IND-15[BAJ14288.1], IND-2[AAG29757.1], IND-2a[ADK25052.1], IND-3[AAG29761.2], IND-4[AAG29765.2], IND-5[AAS78754.1], IND-6[CAJ32373.2], IND-7[BAJ05825.1], IND-8[ACZ65152.1], IND-9[ACZ65153.1]
	NDM	NDM-1[CAZ39946.1], NDM-10[AGT37351.1], NDM-11[AJE61443.1], NDM-12[BAO79439.1], NDM-13[BAQ02518.1], NDM-14[AJP18054.1], NDM-15[AKF43458.1], NDM-16[AKZ20823.1], NDM-2[AEA41876.1], NDM-3[AFK80349.1], NDM-4[AFB82585.1], NDM-5[AEN03071.1], NDM-6[AEX08599.1], NDM-7[AFQ31613.1], NDM-8[BAM84089.1], NDM-9[AGU91756.1]
	VIM	VIM-1[CAB46686.1], VIM-10[AAS13761.1], VIM-11[AAT36613.1], VIM-12[AAZ73123.1], VIM-13[ABC94518.1], VIM-14[AAT48653.1], VIM-15[ACB54702.1], VIM-16[ACB54703.1], VIM-17[ABW90721.1], VIM-18[CAO83029.1], VIM-19[ACY29468.1], VIM-2[AAF61483.1], VIM-20[ACV13198.1], VIM-23[ACT33323.1], VIM-24[ADL27533.1], VIM-25[ADO50679.1], VIM-26[CBY80143.1], VIM-27[ADX78234.1], VIM-28[AEI25539.1], VIM-29[AFP99885.1], VIM-3[AAG27703.1], VIM-30[AET05999.1], VIM-31[AFK24647.1], VIM-32[AEZ49857.1], VIM-33[AFP99175.1], VIM-34[AFN88953.1], VIM-35[AGC50805.1], VIM-36[AGC50806.1], VIM-37[AGC50807.1], VIM-38[AGE83081.2], VIM-39[AGS82586.1], VIM-4[AAN04257.1], VIM-40[CDN33428.1], VIM-41[AKQ98358.1], VIM-42[AJP08641.1], VIM-43[AJP67511.1], VIM-44[AKO63212.1], VIM-45[AKO63211.1], VIM-46[AKQ98357.1], VIM-47[ALK87206.1], VIM-49[AMD11802.1], VIM-5[AAN52134.1], VIM-50[AMD11803.1], VIM-51[AMK97465.1], VIM-6[AAN84550.1], VIM-7[CAD61201.1], VIM-8[AAS13759.1], VIM-9[AAS13760.1]
B2	single	CphA[AAQ60817.1], CphA[EEQ11779.1]
	CphA	CphA[1X8GA], CphA[1X8HA], CphA[1X8IA], CphA[AAB03413.1], CphA[AAM63403.1], CphA[AAP69912.1], CphA[AAP69913.1], CphA[AAP69914.1], CphA[AAP69915.1], CphA[AAP97129.1], CphA[AAP97130.1], CphA[AAP97131.1], CphA[AAP97132.1], CphA[AAP97133.1], CphA[ABE01851.1], CphA[ABE01852.1], CphA[ABK35804.1], CphA[ABO91573.1], CphA[AEB48767.1], CphA[CAA71441.1], CphA[CAD69003.1], CphA[P26918.1]
	SfH	SfH[3Q6VA], SfH[AAF09244.1]
B3	single variants	FEZ[1JT1A], FEZ[1L9YA], FEZ[ABF42880.1], FEZ[ABJ86085.1], FEZ[ABJ87788.1], FEZ[ABQ34648.1], FEZ[ABU77619.1], FEZ[ABV42357.1], FEZ[ACB74399.1], FEZ[ACG76759.1], FEZ[ACG79382.1], FEZ[ADI90897.1], FEZ[ADV84766.1], FEZ[CAB96921.1], FEZ[CAL76016.1], FEZ[CAL76084.1], FEZ[EED30665.1], FEZ[EEF62667.1], FEZ[EET10369.1], FEZ[EET83909.1], FEZ[EEX00045.1], FEZ[EFI53925.1], FEZ[EGF90052.1], FEZ[EGF92793.1], GOB[ACQ71975.1], GOB[ACT91827.1], GOB[ACU62014.1], GOB[EFQ76710.1], L1[EDX89744.1], LRA[ACH58987.1], LRA[ACH58989.1], LRA[ACH58990.1], LRA[ACH58994.1], LRA[ACH58998.1], LRA[ACH59005.1]
	AIM cluster	AIM[296285026], AIM[ABD26183.1], AIM[ABF53616.1], AIM[ACH58985.1], AIM[ACH58988.1], AIM[ACS83721.1], AIM[ACS83724.1], AIM[BAI98728.1], AIM[CAQ53840.1], AIM[EAQ30388.1], AIM[EDL50471.1], SMB[BAL14456.1], THIN-B[ABK64020.1], THIN-B[ABK64103.1], THIN-B[BAH40273.1], THIN-B[CAC33832.1]
	BJP	BJP[2GMNA], BJP[3LVZA]
	CAU	CAU[AAK24110.1], CAU[ADG09779.1], CAU[CAC48262.1], CAU[CAC87665.1], FEZ[ABZ72535.1]
	FEZ	FEZ[213584509], FEZ[213857890], FEZ[238910222], FEZ[289804819], FEZ[AAL22596.1], FEZ[AAO71263.1], FEZ[AAV79390.1], FEZ[AAX67566.1], FEZ[ABX23698.1], FEZ[ABX69950.1], FEZ[ACF63426.1], FEZ[ACF91587.1], FEZ[ACH50729.1], FEZ[ACH75138.1], FEZ[AD0970], FEZ[CAR35138.1], FEZ[EDX47393.1], FEZ[EDX50636.1], FEZ[EDY22774.1], FEZ[EDZ03684.1], FEZ[EDZ07086.1], FEZ[EDZ13659.1], FEZ[EDZ31246.1], FEZ[EDZ37046.1], FEZ[EFY09855.1], FEZ[EGE31827.1], FEZ[EGE36142.1]
	GOB	GOB[AAF04458.1], GOB[AAF89146.1], GOB[AAF89147.1], GOB[AAF89148.1], GOB[AAF89149.1], GOB[AAF89150.1], GOB[AAF89152.1], GOB[AAF89153.1], GOB[AAQ54756.1], GOB[AAT68576.1], GOB[AAT68577.1], GOB[AAT68578.1], GOB[AAT68579.1], GOB[AAT68580.1], GOB[AAT68582.1], GOB[AAV65595.1], GOB[AAW82618.1], GOB[AAW82619.1], GOB[AAY53478.1], GOB[ABO21417.1]
	L1_A	L1[1SMLA], L1[2QINA], L1[2QJSA], L1[AAC21590.1], L1[ABC02083.1], L1[ABO60984.1], L1[ABO60985.1], L1[ABO60986.1], L1[ABO60987.1], L1[ABO60988.1], L1[ABO60989.1], L1[ABO60991.1], L1[ABO60992.1], L1[ABO60993.1], L1[ABO60994.1], L1[ABO60996.1], L1[ABO60998.1], L1[ABO60999.1], L1[ABO61000.1], L1[ABO61003.1], L1[ABW87257.1], L1[ACF51850.1], L1[ADX95742.1], L1[BAE44413.1], L1[BAE44414.1], L1[BAE44415.1], L1[BAE44419.1], L1[BAE44420.1], L1[BAE44421.1], L1[BAF47404.1], L1[BAF47405.1], L1[BAF47406.1], L1[BAF47407.1], L1[BAF47408.1], L1[BAF47409.1], L1[CAB63488.1], L1[CAB63489.1], L1[CAB75346.1], L1[CAB94700.1], L1[CAB94701.1], L1[CAB94702.1], L1[CAB94703.1], L1[CAB94704.1], L1[CAC15063.1], L1[CAQ46142.1], L1[EED40420.1], L1[P52700.1]
	L1_B	L1[ABY56045.1], L1[ADC79554.1], L1[ADC79555.1], L1[ADC79556.1], L1[ADC79557.1], L1[ADC79558.1], L1[ADC79559.1], L1[ADC79560.1], L1[ADC79561.1], L1[ADC79563.1], L1[ADC79565.1], L1[ADC79567.1]

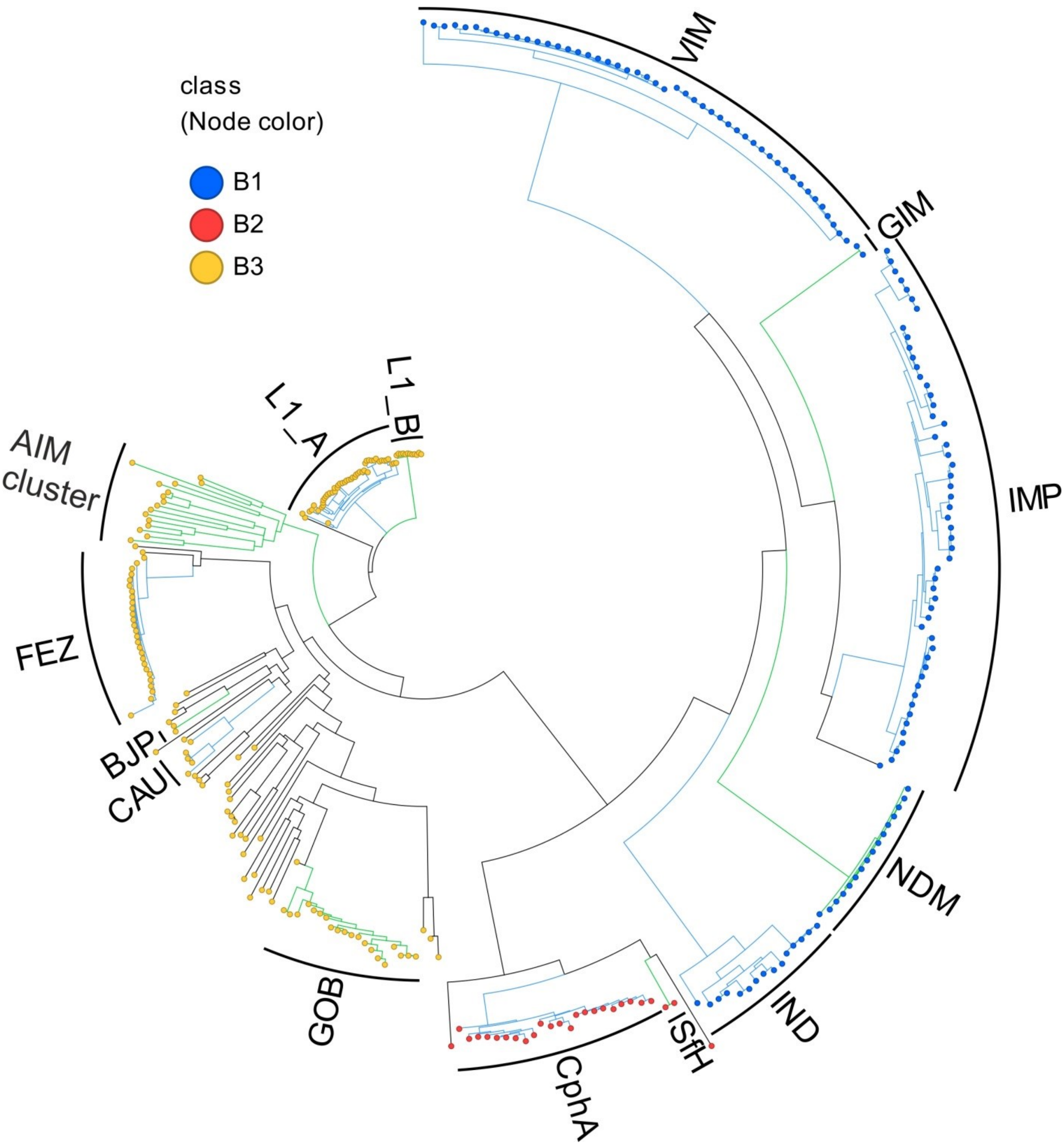

Figure S3: Phylogenetic tree of 324 class B metallo-β-lactamases. Node color represents the respective phenotype; groups are labeled accordingly. Details on the variants of each group can be found in Table S2. Branched lines for each group are alternately colored in gray, green or blue for better visualization only.

Ambler class C

Supplementary Table S3: Overview of the group representatives for the Ambler class C. Members of each group share at least 80 % sequence similarity. For phylogenetic tree see Figure S4.

group	name [accession number_phenotype]
ungrouped chromosomal AmpCs	AmpC_Acinetobacter_baylyi[CAL25116_unknown], AmpC_Aeromonas_hydrophila[YP_857635_unknown], AmpC_Aeromonas_jandaeia[AAA83416_unknown], AmpC_Aeromonas_veronii_bv._sobria[CAA56561_unknown], AmpC_Buttiauxella_agrestis[AAN17791_unknown], AmpC_Chromobacterium_violaceum[NP_900980_unknown], AmpC_Chromohalobacter[BAD16740_unknown], AmpC_Edwardsiella_tarda[ABO48510_unknown], AmpC_Enterobacter_aerogenes[AAO16528_unknown], AmpC_Erwinia_rhapontici[AAP40275_unknown], AmpC_Escherichia_albertii[EDS93081_unknown], AmpC_Escherichia_coli[NP_418574_unknown], AmpC_Escherichia_fergusonii[AAM11671_unknown], AmpC_Laribacter_hongkongensis[AAT46346_unknown], AmpC_Lysobacter_lactamgenus[CAA39987_unknown], AmpC_Mycobacterium_smegmatis[YP_888266_unknown], AmpC_Ochrobactrum_anthropi[CAC04522_unknown], AmpC_Pseudomonas_fluorescens[YP_349452_unknown], AmpC_Psychrobacter_immobilis[CAA58569_unknown], AmpC_Rhodobacter_sphaeroides[YP_355256_unknown], AmpC_Serratia_marcescens[AAK64454_unknown], AmpC_Shigella_boydii[YP_410551_unknown], AmpC_Shigella_dysenteriaec[YP_405772_unknown], AmpC_Shigella_flexneric[YP_691594_unknown], AmpC_Shigella_sonnei[YP_313059_unknown], AmpC_Yersinia_enterocolitica[YP_001006653_unknown], AmpC_Yersinia_mollaretii[ZP_00826692_unknown], AmpC_Yersinia_ruckeri[ABA70720_unknown]
ACC	ACC-1[ACD12694.1_cephalosporinase], ACC-1a[AAF86699.1_cephalosporinase], ACC-1b[AAF86694.1_cephalosporinase], ACC-1c[AAF86698.1_cephalosporinase], ACC-1d[AAF86700.1_cephalosporinase], ACC-2[AAF86691.1_cephalosporinase], ACC-4[AIT76084.1_cephalosporinase], ACC-5[CCK86740.1_cephalosporinase], AmpC_Hafnia_alvei[AAF86691_unknown]
ACT/MIR	ACT-1[AAC45086.2_cephalosporinase], ACT-10[AEV91214.1_cephalosporinase], ACT-12[AFU25650.1_cephalosporinase], ACT-13[CCK86741.1_cephalosporinase], ACT-14[AFU25647.1_cephalosporinase], ACT-15[AFU25653.1_cephalosporinase], ACT-17[AHM76771.1_cephalosporinase], ACT-18[AHM76777.1_cephalosporinase], ACT-19[AHM76779.1_cephalosporinase], ACT-2[CAJ28994.1_cephalosporinase], ACT-20[AHA80105.1_cephalosporinase], ACT-21[AHA80106.1_cephalosporinase], ACT-22[AHM76774.1_cephalosporinase], ACT-23[AGU38146.1_cephalosporinase], ACT-24[AHL39336.1_cephalosporinase], ACT-25[AHL39338.1_cephalosporinase], ACT-27[AHL39340.1_cephalosporinase], ACT-28[AHL39333.1_cephalosporinase], ACT-29[AIT76085.1_cephalosporinase], ACT-3[ABL67017.1_cephalosporinase], ACT-30[AIT76086.1_cephalosporinase], ACT-31[AIT76096.1_cephalosporinase], ACT-32[AIT76088.1_cephalosporinase], ACT-33[AIT76087.1_cephalosporinase], ACT-34[CDP41983.1_cephalosporinase], ACT-35[BAP68758.1_cephalosporinase], ACT-36[AJG06170.1_cephalosporinase], ACT-37[AJG06172.1_cephalosporinase], ACT-38[AKS43590.1_cephalosporinase], ACT-39[AMP42993.1_cephalosporinase], ACT-4[ABZ81086.1_cephalosporinase], ACT-5[ACJ05689.1_cephalosporinase], ACT-6[ACJ05686.1_cephalosporinase], ACT-7[ACJ05688.1_cephalosporinase], ACT-8[CBI75448.1_cephalosporinase], ACT-9[AEI70575.1_cephalosporinase], AmpC_Enterobacter_asburiae[CAC85157_unknown], AmpC_Enterobacter_cancerogenus[AAM11666_unknown], AmpC_Enterobacter_cloacae[P05364_unknown], AmpC_Enterobacter_dissolvens[CAC85359_unknown], AmpC_Enterobacter_hormaechei[CAC85357_unknown], AmpC_Enterobacter_intermediusb[CAC85358_unknown], MIR-1[AAD22636.1_cephalosporinase], MIR-10[AIT76111.1_cephalosporinase], MIR-11[AIT76112.1_cephalosporinase], MIR-12[AIT76116.1_cephalosporinase], MIR-13[AIT76115.1_cephalosporinase], MIR-14[AIT76117.1_cephalosporinase], MIR-15[AIT76104.1_cephalosporinase], MIR-16[AIT76114.1_cephalosporinase], MIR-17[CEA29752.1_cephalosporinase], MIR-18[CEF57506.1_cephalosporinase], MIR-2[AAO42602.1_cephalosporinase], MIR-3[AAU95779.1_cephalosporinase], MIR-4[ABN69112.2_cephalosporinase], MIR-5[ACJ05687.1_cephalosporinase], MIR-6[AFJ79785.1_cephalosporinase], MIR-7[AHL39317.1_cephalosporinase], MIR-9[AIT76113.1_cephalosporinase]
ADC	ADC-1[CAB77444.1_ESBL], ADC-10[ABI18382.1_ESBL], ADC-11[ADG46039.1_ESBL], ADC-12[CAK95249.1_ESBL], ADC-13[CAK95248.2_ESBL], ADC-14[CAK95247.1_ESBL], ADC-15[CAK95246.1_ESBL], ADC-16[CAK95245.1_ESBL], ADC-17[CAK95244.1_ESBL], ADC-18[ACN62075.1_ESBL], ADC-19[CAK95242.1_ESBL], ADC-2[AAO43172.1_unknown], ADC-20[CAK95241.1_ESBL], ADC-21[CAK95240.1_ESBL], ADC-22[CAK95239.1_ESBL], ADC-23[CAK95238.1_ESBL], ADC-25[ABK34773.1_unknown], ADC-26[ADG46043.1_ESBL], ADC-3[AAO59456.1_unknown], ADC-30[ADG46041.1_ESBL], ADC-38[ACC95873.1_unknown], ADC-39[ACC95874.1_unknown], ADC-4[AAO59457.1_unknown], ADC-41[ACN62070.1_ESBL], ADC-42[ACN62071.1_ESBL], ADC-43[ACN62072.1_ESBL], ADC-44[ACN62073.1_ESBL], ADC-5[CAE00827.1_unknown], ADC-50[ADG46038.1_ESBL], ADC-51[ADG46040.1_ESBL], ADC-52[ADG46042.1_ESBL], ADC-53[ADG46044.1_ESBL], ADC-54[ADK35761.1_unknown], ADC-56[AEL30570.1_ESBL], ADC-58[AFG25594.1_unknown], ADC-59[AFG25595.1_unknown], ADC-6[AAR13676.1_unknown], ADC-60[AFH53180.1_unknown], ADC-61[AFI56570.1_unknown], ADC-62[AFK24475.1_unknown], ADC-63[AFM80040.1_unknown], ADC-64[AFM80041.1_unknown], ADC-67[AFP89364.1_unknown], ADC-68[AGL39360.1_carbapenemase], ADC-7[AAT70411.1_unknown], ADC-73[ALA14808.1_ESBL], ADC-74[ALA14809.1_ESBL], ADC-75[ALA14810.1_ESBL], ADC-76[ALA14811.1_ESBL], ADC-77[ALA14812.1_ESBL], ADC-78[ALA14813.1_ESBL], ADC-79[ALA14814.1_ESBL], ADC-80[ALA14815.1_ESBL], ADC-81[ALA14816.1_ESBL], AmpC_Acinetobacter_baumannii[CAB77444_unknown]
CMY	AmpC_Citrobacter_braakii[AAM11668_unknown], AmpC_Citrobacter_freundii[AAM93471_unknown], AmpC_Citrobacter_murliniae[AAM11664_unknown], AmpC_Citrobacter_werkmanii[AAM11670_unknown], AmpC_Citrobacter_youngae[CAD32304_unknown], CFE-1[BAC76072.1_cephalosporinase], CMY-100[AHA80101.1_unknown], CMY-101[AHA80102.1_unknown], CMY-102[AHA80103.1_unknown], CMY-103[AHA80104.1_unknown], CMY-104[AGR82311.1_unknown], CMY-105[AHL39330.1_unknown], CMY-106[AJH76980.1_unknown], CMY-107[ALE33733.1_unknown], CMY-108[AGZ20169.1_unknown], CMY-109[CAG34070.1_unknown], CMY-110[BAO05497.1_unknown], CMY-111[AHW47897.1_unknown], CMY-112[AIT76090.1_unknown], CMY-113[AIT76089.1_unknown], CMY-114[AIT76099.1_unknown], CMY-115[AIT76092.1_unknown], CMY-116[AIT76093.1_unknown], CMY-117[AIT76097.1_unknown], CMY-118[AIT76091.1_unknown], CMY-119[AIT76098.1_unknown], CMY-12[CAA76382.1_unknown], CMY-121[AIZ48988.1_unknown], CMY-122[AKO62859.1_unknown], CMY-124[AKO62861.1_unknown], CMY-125[AKO62862.1_unknown], CMY-127[AKO62864.1_unknown], CMY-128[AKO62865.1_unknown], CMY-129[AKO62866.1_unknown], CMY-13[AAQ16660.2_unknown], CMY-130[AKI06385.1_unknown], CMY-131[AKG51729.1_unknown], CMY-132[AKZ20822.1_unknown], CMY-133[AKZ20821.1_unknown], CMY-134[AKA60779.1_unknown], CMY-135[AKP17985.1_unknown], CMY-138[ALM96710.1_unknown], CMY-139[AMK49571.1_unknown], CMY-14[CAD88479.1_unknown], CMY-15[CAD88477.1_unknown], CMY-16[CAH03679.1_unknown], CMY-17[AAS13399.1_unknown], CMY-18[AAU95778.1_unknown], CMY-2[CAA62957.1_unknown], CMY-20[AAX58682.2_unknown], CMY-21[AAZ66866.1_unknown],

	CMY-22[ABB72431.1_unknown], CMY-23[ABF06441.1_unknown], CMY-24[ABN69070.1_unknown], CMY-25[ACA30420.1_unknown], CMY-26[BAF56185.1_unknown], CMY-27[ACA30421.1_unknown], CMY-28[ABQ51091.1_unknown], CMY-29[ABS12248.1_unknown], CMY-30[ABS12249.1_ESBL], CMY-31[ABR18736.1_unknown], CMY-32[ACA97846.1_unknown], CMY-33[ACA97847.1_unknown], CMY-34[ABN51006.1_unknown], CMY-35[ABN51007.1_unknown], CMY-36[ABY58049.1_unknown], CMY-37[BAF36388.1_ESBL], CMY-38[CAP60699.1_unknown], CMY-39[BAF95726.1_unknown], CMY-4[CAA75402.1_unknown], CMY-40[ACA30422.1_unknown], CMY-41[BAG14343.1_unknown], CMY-42[ADM21467.1_ESBL], CMY-43[ACJ05361.1_unknown], CMY-44[ACJ65711.1_unknown], CMY-45[CBB16411.1_unknown], CMY-46[CBH19182.1_unknown], CMY-47[ADH82410.1_unknown], CMY-48[ADP02979.1_unknown], CMY-49[ACV32310.1_unknown], CMY-5[CAB50867.1_unknown], CMY-50[CBI75447.1_unknown], CMY-51[AFK73431.1_unknown], CMY-53[ADQ38362.1_unknown], CMY-54[ADK55604.1_unknown], CMY-55[ADK55605.1_unknown], CMY-56[ADT91162.1_unknown], CMY-57[ADP37961.1_unknown], CMY-58[ADY19254.1_unknown], CMY-6[CAB36902.1_unknown], CMY-60[AEM97672.1_unknown], CMY-61[AEM97673.1_unknown], CMY-62[AEM97674.1_unknown], CMY-63[AET07387.1_unknown], CMY-64[ADW84690.1_unknown], CMY-65[AEI52842.1_unknown], CMY-66[AEZ49849.1_unknown], CMY-67[AFK08541.1_unknown], CMY-68[AEZ49855.1_unknown], CMY-69[AGE45504.1_unknown], CMY-7[CAB36900.1_unknown], CMY-70[AFU25635.1_unknown], CMY-71[AFK08538.1_unknown], CMY-72[AFU25641.1_unknown], CMY-73[ACU00152.1_unknown], CMY-74[AFU25632.1_unknown], CMY-75[AFK73434.1_unknown], CMY-76[AFK73437.1_unknown], CMY-77[AFU25644.1_unknown], CMY-78[AFK73443.1_unknown], CMY-79[AFK73446.1_unknown], CMY-80[AFK73449.1_unknown], CMY-81[AFK73452.1_unknown], CMY-82[AHL39324.1_unknown], CMY-83[AFU25638.1_unknown], CMY-84[AFK73455.1_unknown], CMY-85[AHL39322.1_unknown], CMY-86[AHL39327.1_unknown], CMY-87[BAL63057.1_unknown], CMY-89[CCK86742.1_unknown], CMY-90[CCK86743.1_unknown], CMY-93[AHM76768.1_unknown], CMY-94[AGC54798.1_unknown], CMY-95[AGC54799.1_unknown], CMY-96[AFZ85212.1_unknown], CMY-97[AFZ85213.1_unknown], CMY-99[AGU59995.1_unknown], LAT-1[CAA55007.1_cephalosporinase], LAT-3[AAB80855.1_cephalosporinase]
**DHA**	AmpC_Morganella_morganii[AAC68582_unknown], AmpC_Providencia_stuartii[CAA76739_unknown], DHA-1[CAA76196.1_unknown], DHA-10[AJO16043.1_unknown], DHA-12[CDL68900.1_unknown], DHA-13[AIT76108.1_unknown], DHA-14[AIT76107.1_unknown], DHA-15[AIT76106.1_unknown], DHA-16[AIT76105.1_unknown], DHA-17[AIT76103.1_unknown], DHA-18[AIT76094.1_unknown], DHA-19[AIT76102.1_unknown], DHA-2[AAG36927.1_unknown], DHA-20[AIT76101.1_unknown], DHA-21[AIT76100.1_unknown], DHA-22[AIT76109.1_unknown], DHA-23[AMY61251.1_unknown], DHA-24[AMM39807.1_unknown], DHA-3[AAR87489.1_unknown], DHA-4[KGZ25601.1_unknown], DHA-5[AEL22919.1_unknown], DHA-6[ADT91161.1_unknown], DHA-7[ADQ00385.1_unknown], DHA-9[AHL39320.1_unknown]
**FOX**	AmpC_Aeromonas_caviae[AAM46773_unknown], AmpC_Aeromonas_salmonicida[ABO89301_unknown], FOX-1[CAA54602.1_cephalosporinase], FOX-10[AGE45503.1_cephalosporinase], FOX-12[CDP41984.1_cephalosporinase], FOX-13[AMN16527.1_cephalosporinase], FOX-2[CAA71325.1_cephalosporinase], FOX-3[CAA71947.1_cephalosporinase], FOX-4[CAB89086.1_cephalosporinase], FOX-5[AAG12974.1_cephalosporinase], FOX-7[CAG28256.1_cephalosporinase], FOX-8[ADK73994.1_cephalosporinase], FOX-9[AEK78851.1_cephalosporinase]
**MOX**	CMY-1[CAA63264.1_unknown], CMY-10[AAK31368.1_unknown], CMY-11[AAK31370.1_unknown], CMY-19[BAE48233.1_unknown], CMY-8[AAD50818.2_ESBL], CMY-8b[AAZ03413.1_unknown], CMY-9[BAB72158.1_ESBL], MOX-1[BAA02563.2_unknown], MOX-10[CEF57507.1_unknown], MOX-11[CEF57508.1_unknown], MOX-12[KGY70007.1_unknown], MOX-2[CAB82578.1_unknown], MOX-3[ACA30419.1_unknown], MOX-4[ACI89425.1_unknown], MOX-5[ACS44783.1_unknown], MOX-6[ACS44784.1_unknown], MOX-7[ACS44785.1_unknown], MOX-8[AGH56079.1_unknown], MOX-9[AIG22447.1_unknown]
**PDC**	AmpC_Pseudomonas_aeruginosa[NP_252799_unknown], PDC-1[ACQ82807.1_cephalosporinase], PDC-10[ACQ82815.1_unknown], PDC-11[ACX31161.1_unknown], PDC-12[AEM44534.1_unknown], PDC-13[ACX31163.1_unknown], PDC-14[ACX31164.1_unknown], PDC-15[ACX31165.1_unknown], PDC-16[ADT64395.1_unknown], PDC-17[ACX31167.1_unknown], PDC-18[ADC96714.1_unknown], PDC-19a[CBZ41769.1_unknown], PDC-19b[AEM44530.1_unknown], PDC-2[ACQ82806.1_ESBL], PDC-20[AEM44531.1_unknown], PDC-21a[CBZ41771.1_unknown], PDC-21b[AEM44532.1_unknown], PDC-22[CBZ41772.1_unknown], PDC-23[CBZ41773.1_unknown], PDC-24[AEM44535.1_unknown], PDC-25[AEM44536.1_unknown], PDC-26[AEM44537.1_unknown], PDC-28[AIG19968.1_unknown], PDC-3[CAW25588.1_ESBL], PDC-30[AIG19969.1_unknown], PDC-31[AIG19970.1_unknown], PDC-32[BAE46546.1_unknown], PDC-34[AIG19971.1_unknown], PDC-35[CBZ41770.1_unknown], PDC-36[AIG19973.1_unknown], PDC-37[AIG19974.1_unknown], PDC-38[AIG19975.1_unknown], PDC-39[AIG19976.1_unknown], PDC-4[ACQ82809.1_ESBL], PDC-40[AIG19977.1_unknown], PDC-43[AIG19980.1_unknown], PDC-44[AIG19981.1_unknown], PDC-45[AIG19982.1_unknown], PDC-46[AIG19983.1_unknown], PDC-47[AIG19984.1_unknown], PDC-48[AIG19985.1_unknown], PDC-49[AIG19986.1_unknown], PDC-5[ACQ82810.1_ESBL], PDC-50[AIG19987.1_unknown], PDC-51[AIG19988.1_unknown], PDC-52[AIG19989.1_unknown], PDC-53[AIG19990.1_unknown], PDC-54[AIG19991.1_unknown], PDC-55[AIG19992.1_unknown], PDC-56[AIG19993.1_unknown], PDC-57[AIG19994.1_unknown], PDC-58[AIG19995.1_unknown], PDC-59[AIG19996.1_unknown], PDC-6[ACQ82811.1_unknown], PDC-60[AIG19997.1_unknown], PDC-61[AIG19998.1_unknown], PDC-63[AIG20000.1_unknown], PDC-64[AIG20001.1_unknown], PDC-65[AIG20002.1_unknown], PDC-66[AIG20003.1_unknown], PDC-67[AIG20004.1_unknown], PDC-68[AIG20005.1_unknown], PDC-69[AIG20006.1_unknown], PDC-7[ACQ82812.1_unknown], PDC-70[AIG20007.1_unknown], PDC-71[AIG20008.1_unknown], PDC-72[AIG20009.1_unknown], PDC-8[ACQ82813.1_unknown], PDC-9[ACQ82814.1_unknown], PDC-94[AMR55736.1_unknown], PDC-95[AMR55738.1_unknown], PDC-96[AMR55739.1_unknown], PDC-97[AMR55740.1_unknown]

Chromosomal AmpCs retrieved from [1]

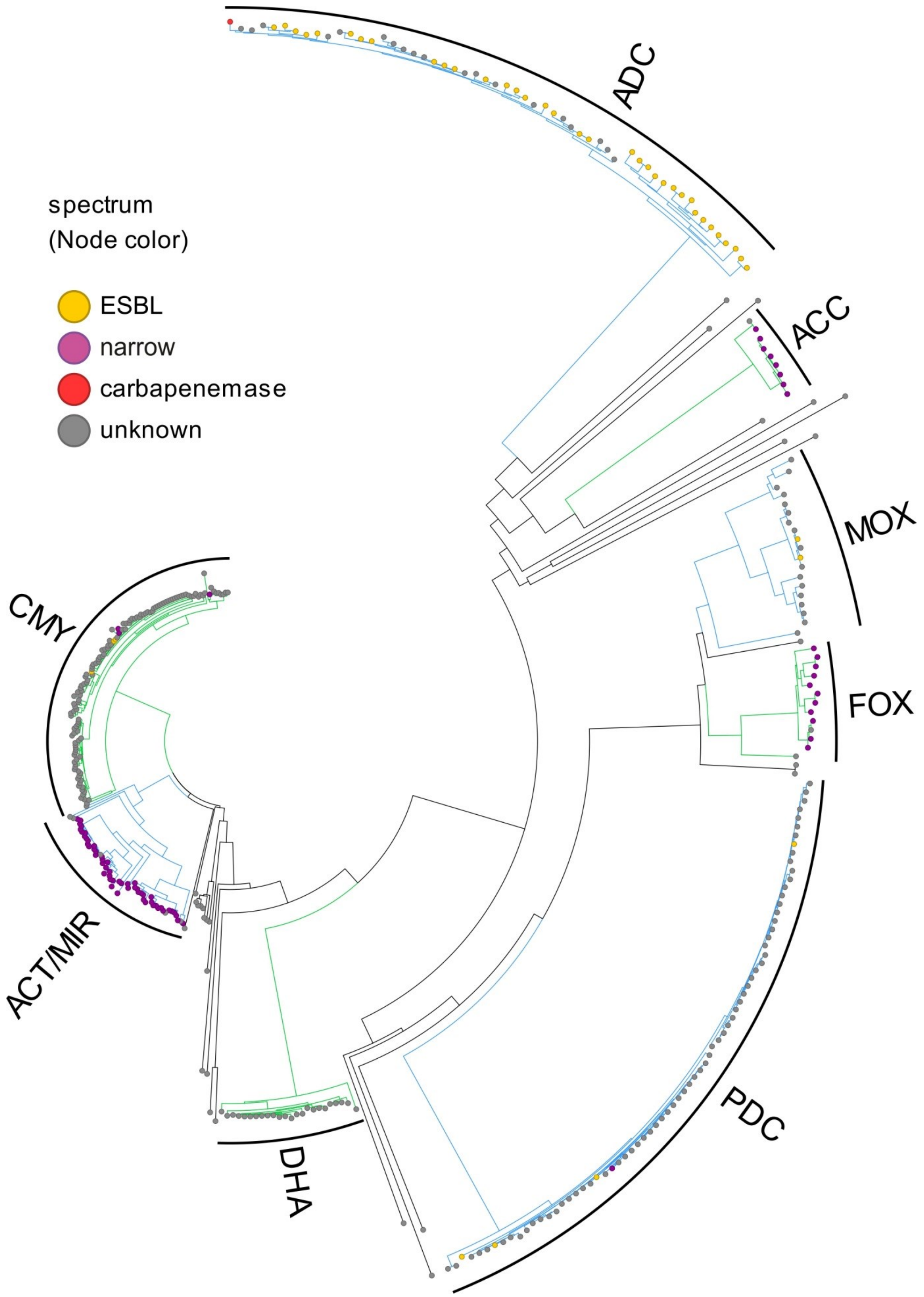

Supplementary Figure S4: Phylogenetic tree of 409 class C β-lactamases. Node color represents the respective phenotype; groups are labeled accordingly. Details on the variants of each group can be found in Table S3. Branched lines for each group are alternately colored in gray, green or blue for better visualization only.

Ambler class D

Supplementary Table S4: Overview of the group representatives for the Ambler class D. Members of each group share at least 80 % sequence similarity. For phylogenetic tree see Figure S5.

group	name[accession_resistance]
1	OXA-1[AAA91586.2_narrow], OXA-224[AEQ73521.1_unknown], OXA-31[AAK52604.1_ESBL], OXA-320[AGR55864.1_unknown], OXA-392[AAO84013.1_unknown], OXA-4[AAR12140.1_narrow], OXA-47[AAP69225.1_narrow]
2	OXA-141[ABQ15112.1_ESBL], OXA-15[AAB05874.1_ESBL], OXA-161[ACT09125.1_ESBL], OXA-2[CAA30246.1_narrow], OXA-21[CAA71699.2_narrow], OXA-210[AEE61368.1_unknown], OXA-226[ACM67635.1_ESBL], OXA-3[AAC41449.1_narrow], OXA-32[AAK58418.1_ESBL], OXA-415[AIG94927.1_unknown], OXA-53[AAP43641.1_ESBL]
10	OXA-10[AAB60534.1_narrow], OXA-101[CAL85435.1_unknown], OXA-11[CAA80304.1_ESBL], OXA-129[CAP69660.1_unknown], OXA-13[AAC46344.1_narrow], OXA-142[ABY79006.1_ESBL], OXA-145[ACN85419.1_ESBL], OXA-147[ACO72579.1_ESBL], OXA-17[ABI63579.1_ESBL], OXA-183[ADV41666.1_unknown], OXA-19[AAD02245.1_ESBL], OXA-233[AID67109.1_carbapenemase], OXA-240[AFN20670.1_unknown], OXA-246[AHC31001.1_unknown], OXA-251[AER57903.1_unknown], OXA-256[CCE94500.1_unknown], OXA-28[AAF72942.1_ESBL], OXA-35[AAK49460.1_ESBL], OXA-454[BAR45714.1_unknown], OXA-5[CAA41211.1_narrow], OXA-56[AAR32651.1_narrow], OXA-7[CAA53242.1_narrow], OXA-74[CAH69530.1_unknown]
20	OXA-20[AAC23554.1_narrow], OXA-37[AAG33665.1_narrow]
22	OXA-22[AAD12233.1_narrow], OXA-443[BAQ55593.2_unknown]
23	OXA-103[ENV88740.1_carbapenemase], OXA-133[ACE63186.1_carbapenemase], OXA-146[ACI28281.1_carbapenemase], OXA-165[ADK35869.1_carbapenemase], OXA-166[ADK35870.1_carbapenemase], OXA-167[ADK35871.1_carbapenemase], OXA-168[ADK35872.1_carbapenemase], OXA-169[ADK35873.1_carbapenemase], OXA-170[ADK35874.1_carbapenemase], OXA-171[ADK35875.1_carbapenemase], OXA-225[AEP43731.1_carbapenemase], OXA-23[CAB69042.1_carbapenemase], OXA-239[AFN65709.1_carbapenemase], OXA-27[AAG35609.2_carbapenemase], OXA-366[AJO16038.1_carbapenemase], OXA-398[AIT76095.1_carbapenemase], OXA-422[AIY30331.1_carbapenemase], OXA-423[AIY30332.1_carbapenemase], OXA-435[AJT57878.1_carbapenemase], OXA-440[AKR53962.1_carbapenemase], OXA-482[AKF41839.1_carbapenemase], OXA-483[AKF41840.1_carbapenemase], OXA-49[AAP40270.1_carbapenemase], OXA-73[AAW22056.1_carbapenemase]
40 or 24* (143)#	OXA-139[CAQ51348.1_carbapenemase], OXA-143[ACX70402.1_carbapenemase], OXA-160[ADB28891.1_carbapenemase], OXA-182[ADK92148.1_carbapenemase], OXA-207[AFK28473.1_carbapenemase], OXA-231[AFG29918.1_carbapenemase], OXA-24[AAM34291.1_carbapenemase], OXA-25[AAG35607.1_carbapenemase], OXA-253[AGK07368.1_carbapenemase], OXA-255[AGK07369.1_carbapenemase], OXA-26[AAG35608.1_carbapenemase], OXA-437[AKL59536.1_carbapenemase], OXA-499[ALM96709.1_carbapenemase], OXA-72[ABP87779.2_carbapenemase]
42	OXA-42[CAD32564.1_narrow], OXA-43[CAD32565.1_narrow], OXA-57[CAF74882.1_narrow], OXA-59[CAG15145.1_narrow]
46	OXA-118[AAK55330.1_narrow], OXA-119[AAN41427.1_narrow], OXA-205[AEO92079.1_unknown], OXA-46[AAN63499.1_narrow]
48	OXA-162[ADG27454.1_carbapenemase], OXA-163[ADY06444.1_ESBL], OXA-181[AEP16366.1_carbapenemase], OXA-199[AFC95894.1_carbapenemase], OXA-204[AFU91598.1_carbapenemase], OXA-232[AGD91915.1_carbapenemase], OXA-244[AGC60012.1_carbapenemase], OXA-245[AGC60013.1_carbapenemase], OXA-247[AGC70814.1_carbapenemase], OXA-252[WP_037428895.1_unknown], OXA-370[AHF71363.1_unknown], OXA-405[AJA30430.1_ESBL], OXA-416[AKH90740.1_unknown], OXA-436[ALN39165.1_unknown], OXA-438[AKL59521.1_unknown], OXA-439[AKR53961.1_unknown], OXA-48[AAP70012.1_carbapenemase], OXA-484[ALI16502.1_unknown], OXA-505[ALZ40809.1_unknown], OXA-54[AAR89917.1_carbapenemase]
50	OXA-395[AAQ76280.1_narrow], OXA-396[AAQ76282.1_narrow], OXA-485[KSG40284.1_narrow], OXA-486[KJJ14577.1_narrow], OXA-488[AAQ76279.1_narrow], OXA-494[KSC07307.1_narrow], OXA-50[AAQ76277.1_narrow]
51	OXA-100[CAJ77817.2_carbapenemase], OXA-104[ABQ52429.1_carbapenemase], OXA-106[ABV31686.1_carbapenemase], OXA-107[ABV31687.1_carbapenemase], OXA-108[ABV31688.1_carbapenemase], OXA-109[ABV31689.1_carbapenemase], OXA-110[ABV31690.1_carbapenemase], OXA-111[ABV31691.1_carbapenemase], OXA-112[ABV31692.1_carbapenemase], OXA-113[ABW70410.1_carbapenemase], OXA-115[ABS71123.1_carbapenemase], OXA-117[ACV49885.1_carbapenemase], OXA-120[CCJ32595.1_carbapenemase], OXA-121[ABX45060.1_carbapenemase], OXA-122[ABX45061.1_carbapenemase], OXA-123[ABX45062.1_carbapenemase], OXA-124[ABX45063.1_carbapenemase], OXA-125[ABX45064.1_carbapenemase], OXA-126[ABX45065.1_carbapenemase], OXA-127[ABX45066.1_carbapenemase], OXA-128[ABY77907.1_carbapenemase], OXA-130[ACD84988.1_carbapenemase], OXA-131[ACD84989.1_carbapenemase], OXA-132[ACD84990.1_carbapenemase], OXA-138[ACD45467.1_carbapenemase], OXA-144[ACY56711.1_carbapenemase], OXA-148[ACX31140.1_carbapenemase], OXA-149[ACX31141.1_carbapenemase], OXA-150[ACX31142.1_carbapenemase], OXA-172[ADI58616.1_carbapenemase], OXA-173[ADI58617.1_carbapenemase], OXA-174[ADI58618.1_carbapenemase], OXA-175[ADI58619.1_carbapenemase], OXA-176[ADI58620.1_carbapenemase], OXA-177[ADI58621.1_carbapenemase], OXA-178[ADI58622.1_carbapenemase], OXA-179[ADM26744.1_carbapenemase], OXA-180[ADM26745.1_carbapenemase], OXA-194[AEB98920.1_carbapenemase], OXA-195[AEB98921.1_carbapenemase], OXA-196[AEB98922.1_carbapenemase], OXA-197[AEB98923.1_carbapenemase], OXA-200[ADX07745.1_carbapenemase], OXA-201[ADX07746.1_carbapenemase], OXA-202[ADX07747.1_carbapenemase], OXA-203[ADX07748.1_carbapenemase], OXA-206[BAL15076.1_carbapenemase], OXA-208[CCA94644.1_carbapenemase], OXA-216[CCA94649.1_carbapenemase], OXA-217[AEO44980.1_carbapenemase], OXA-219[AEL17179.1_carbapenemase], OXA-223[AEL88491.1_carbapenemase], OXA-234[WP_038350216.1_carbapenemase], OXA-241[AFO55201.1_carbapenemase], OXA-242[AFO55202.1_carbapenemase], OXA-248[CCJ32596.1_carbapenemase], OXA-249[CCJ32597.1_carbapenemase], OXA-250[CCJ32598.1_carbapenemase], OXA-254[BAN10684.1_carbapenemase], OXA-259[ENW61294.1_carbapenemase], OXA-260[ENU54757.1_carbapenemase], OXA-261[ENW35072.1_carbapenemase], OXA-262[ENW50687.1_carbapenemase], OXA-263[ENW43915.1_carbapenemase], OXA-312[AGU69250.1_carbapenemase], OXA-313[AGU69251.1_carbapenemase], OXA-314[AGU69252.1_carbapenemase], OXA-315[AGU69253.1_carbapenemase], OXA-316[AGU69254.1_carbapenemase], OXA-317[AGU69255.1_carbapenemase], OXA-336[AHN07452.1_carbapenemase], OXA-337[EXR49497.1_carbapenemase], OXA-338[AHN07454.1_carbapenemase], OXA-339[AHN07456.1_carbapenemase], OXA-340[AHN07457.1_carbapenemase], OXA-341[AHN07458.1_carbapenemase], OXA-342[AHN07460.1_carbapenemase], OXA-343[AHN07461.1_carbapenemase], OXA-344[AHN07462.1_carbapenemase], OXA-345[AHN07463.1_carbapenemase], OXA-346[AHN07464.1_carbapenemase], OXA-365[AHI63011.1_carbapenemase], OXA-371[BAO09635.1_carbapenemase], OXA-374[AHL30274.1_carbapenemase], OXA-375[AHL30275.1_carbapenemase], OXA-376[AHL30276.1_carbapenemase], OXA-377[AHL30277.1_carbapenemase], OXA-378[AHL30278.1_carbapenemase], OXA-379[AHL30279.1_carbapenemase], OXA-380[AHL30280.1_carbapenemase], OXA-381[AHL30285.1_carbapenemase], OXA-382[AHL30286.1_carbapenemase], OXA-383[AHL30281.1_carbapenemase], OXA-384[AHL30282.1_carbapenemase], OXA-385[AHL30272.1_carbapenemase], OXA-386[AHL30273.1_carbapenemase], OXA-387[AHK51384.1_carbapenemase], OXA-388[AHL30284.1_carbapenemase], OXA-389[AHL30287.1_carbapenemase], OXA-390[AHL30283.1_carbapenemase], OXA-391[AHN53381.1_carbapenemase], OXA-400[AIS39036.1_carbapenemase], OXA-401[AIS39034.1_carbapenemase], OXA-402[AIS39035.1_carbapenemase], OXA-403[AIM47095.1_carbapenemase], OXA-404[AIM47096.1_carbapenemase], OXA-406[AJD07402.1_carbapenemase], OXA-407[KQF55411.1_carbapenemase], OXA-408[AJD07404.1_carbapenemase], OXA-409[AJD07405.1_carbapenemase], OXA-411[AJD07406.1_carbapenemase], OXA-412[AJD07407.1_carbapenemase], OXA-413[AJD07408.1_carbapenemase], OXA-414[AJD07401.1_carbapenemase],

	OXA-424[AJA32742.1_carbapenemase], OXA-425[AJA32743.1_carbapenemase], OXA-426[AJA32744.1_carbapenemase], OXA-429[AJG01375.1_carbapenemase], OXA-430[AJG01376.1_carbapenemase], OXA-431[AJG01377.1_carbapenemase], OXA-432[AJG01378.1_carbapenemase], OXA-433[AJG01379.1_carbapenemase], OXA-441[KCY13953.1_carbapenemase], OXA-442[AKU37610.1_carbapenemase], OXA-480[ALL53530.1_carbapenemase], OXA-497[KRW26153.1_carbapenemase], OXA-507[AMB17204.1_carbapenemase], OXA-508[AMB57506.1_carbapenemase], OXA-509[AMJ17435.1_carbapenemase], OXA-51[CAC83905.2_carbapenemase], OXA-516[AMO28427.1_carbapenemase], OXA-64[AAW81336.1_carbapenemase], OXA-65[AAW81337.1_carbapenemase], OXA-66[AAW81338.1_carbapenemase], OXA-67[ABF50983.1_carbapenemase], OXA-68[AAW81339.1_carbapenemase], OXA-69[AAW81340.1_carbapenemase], OXA-70[AAW81341.1_carbapenemase], OXA-71[AAW81342.1_carbapenemase], OXA-75[AAW51373.1_carbapenemase], OXA-76[AAX51234.1_carbapenemase], OXA-77[AAX51233.1_carbapenemase], OXA-78[AAW81358.1_carbapenemase], OXA-79[ABV71246.1_carbapenemase], OXA-80[ABV71247.1_carbapenemase], OXA-82[ABV71248.1_carbapenemase], OXA-83[ABC26007.1_carbapenemase], OXA-84[ABC26006.1_carbapenemase], OXA-86[AAZ78361.1_carbapenemase], OXA-87[ABC84263.1_carbapenemase], OXA-88[ABD48715.1_carbapenemase], OXA-89[ABE03012.1_carbapenemase], OXA-90[CAJ77809.1_carbapenemase], OXA-91[ABF47914.1_carbapenemase], OXA-92[ABC61637.1_carbapenemase], OXA-93[ABF47916.1_carbapenemase], OXA-94[ABF47917.1_carbapenemase], OXA-95[ABF47918.1_carbapenemase], OXA-98[CAK50801.1_carbapenemase], OXA-99[ABI53716.1_carbapenemase]
**58**	OXA-164[ADK34116.1_carbapenemase], OXA-397[AIT76118.1_carbapenemase], OXA-420[BAP28835.1_carbapenemase], OXA-512[AMK37641.1_carbapenemase], OXA-58[AAW57529.1_carbapenemase], OXA-96[ABF47919.1_carbapenemase], OXA-97[ABO33299.1_carbapenemase]
**60**	OXA-444[BAQ55594.1_carbapenemase], OXA-60[AAQ08905.1_carbapenemase]
**61**	OXA-193[CAL34450.1_unknown], OXA-450[AKI29914.1_unknown], OXA-451[AKI29916.1_unknown], OXA-452[AKI29917.1_unknown], OXA-453[AKI29919.1_unknown], OXA-460[AKI29920.1_unknown], OXA-461[AKI29921.1_unknown], OXA-489[EIA89747.1_unknown], OXA-61[AAT01092.1_unknown]
**62**	OXA-151[ALA99185.1_carbapenemase], OXA-152[ALA99186.1_carbapenemase], OXA-62[AAR32134.1_carbapenemase]
**63**	OXA-136[ABW76134.1_narrow], OXA-137[ABW76138.1_narrow], OXA-192[ADZ54048.1_narrow], OXA-470[ALC79285.1_narrow], OXA-471[ABW76137.1_narrow], OXA-472[ALC79287.1_narrow], OXA-473[ALC79288.1_narrow], OXA-474[ALC79289.1_narrow], OXA-475[ALC79290.1_narrow], OXA-476[ALC79291.1_narrow], OXA-477[ALC79292.1_narrow], OXA-478[ALC79293.1_narrow], OXA-479[ALC79294.1_narrow], OXA-63[AAU88145.1_narrow]
**114**	OXA-114a[ABX38721.1_narrow], OXA-114b[ADI23933.1_unknown], OXA-114c[ADI23934.1_unknown], OXA-114d[ADI25068.1_unknown], OXA-114e[ADI25069.1_unknown], OXA-114f[ADO14462.1_unknown], OXA-114g[ADO14463.1_unknown], OXA-243[AFQ90085.1_unknown], OXA-258[CCE73593.2_unknown], OXA-364[AFR53898.2_unknown], OXA-455[ALC76097.1_narrow], OXA-457[ALC76099.1_unknown], OXA-458[ALC76100.1_unknown], OXA-459[ALC76101.1_unknown], OXA-513[ALX18471.1_narrow]
**134 (235)#**	OXA-134[ADM47435.1_carbapenemase], OXA-235[AFH36330.1_carbapenemase], OXA-236[AFH36332.1_carbapenemase], OXA-237[AFH36331.1_carbapenemase], OXA-276[ENV13583.1_carbapenemase], OXA-277[ENV45890.1_carbapenemase], OXA-278[AGI65307.1_carbapenemase], OXA-282[ENU64030.1_carbapenemase], OXA-283[ENU15301.1_carbapenemase], OXA-284[ENX16434.1_carbapenemase], OXA-285[ENX26326.1_carbapenemase], OXA-335[AGW16417.1_carbapenemase], OXA-360[AGZ83156.1_carbapenemase], OXA-361[AHA11124.1_carbapenemase], OXA-362[AHA11125.1_carbapenemase], OXA-363[AHA11126.1_carbapenemase]
**154**	OXA-154[ALA99188.1_carbapenemase], OXA-155[ALA99189.1_carbapenemase]
**156**	OXA-156[ALA99190.1_carbapenemase], OXA-158[ALA99192.1_carbapenemase], OXA-159[ALA99193.1_carbapenemase]
**184**	OXA-184[AFO09968.1_unknown], OXA-185[AFO09969.1_unknown], OXA-446[AKI29907.1_unknown], OXA-447[AKI29908.1_unknown], OXA-448[AKI29909.1_unknown], OXA-449[AKI29910.1_unknown], OXA-465[AKI29912.1_unknown], OXA-466[AKI29913.1_unknown]
**211**	OXA-211[AEV91550.1_carbapenemase], OXA-212[AEV91551.1_carbapenemase], OXA-280[ENV74419.1_carbapenemase], OXA-281[ENU38533.1_carbapenemase], OXA-309[CCW28916.1_carbapenemase], OXA-333[AGW16415.1_carbapenemase], OXA-334[AGW16416.1_carbapenemase], OXA-373[CDM87362.1_carbapenemase]
**213**	OXA-213[AEV91552.1_carbapenemase], OXA-267[ENV93423.1_carbapenemase], OXA-268[ENU09033.1_carbapenemase], OXA-269[EOQ63861.1_carbapenemase], OXA-270[EOQ69512.1_carbapenemase], OXA-271[EOQ72432.1_carbapenemase], OXA-272[ENW16915.1_carbapenemase], OXA-273[ENW12288.1_carbapenemase], OXA-304[ENX45276.1_carbapenemase], OXA-305[ENV02983.1_carbapenemase], OXA-322[AGW16404.1_carbapenemase], OXA-323[AGW16405.1_carbapenemase], OXA-324[AGW16406.1_carbapenemase], OXA-325[AGW16407.1_carbapenemase], OXA-326[AGW16408.1_carbapenemase], OXA-327[AGW16409.1_carbapenemase], OXA-328[AGW16410.1_carbapenemase], OXA-329[AGW16411.1_carbapenemase], OXA-330[AGW16412.1_carbapenemase], OXA-331[AGW16413.1_carbapenemase], OXA-332[AGW16414.1_carbapenemase], OXA-348[AGW83446.1_carbapenemase], OXA-349[AGW83447.1_carbapenemase], OXA-350[AGW83448.1_carbapenemase], OXA-351[AGW83449.1_carbapenemase], OXA-352[AGW83450.1_carbapenemase], OXA-353[AGW83451.1_carbapenemase], OXA-354[AGW83452.1_carbapenemase], OXA-357[AGZ83153.1_carbapenemase], OXA-358[AGZ83154.1_carbapenemase], OXA-359[AGZ83155.1_carbapenemase], OXA-417[AIU44173.1_carbapenemase], OXA-421[AIZ00987.1_carbapenemase], OXA-500[ALP13523.1_carbapenemase], OXA-501[ALP13525.1_carbapenemase], OXA-502[ALP13526.1_carbapenemase], OXA-503[ALP13524.1_carbapenemase], OXA-506[AMB17205.1_carbapenemase]
**214**	OXA-214[AEV91553.1_carbapenemase], OXA-215[AEV91554.1_carbapenemase], OXA-264[ENW20318.1_carbapenemase], OXA-265[ENW22086.1_carbapenemase]
**229**	OXA-228[AFM55001.1_carbapenemase], OXA-229[AFM55000.1_carbapenemase], OXA-230[AFM55002.1_carbapenemase], OXA-257[AGK07370.1_carbapenemase], OXA-274[ENU56528.1_unknown], OXA-275[ENV18923.1_unknown], OXA-300[ENV20093.1_carbapenemase], OXA-301[ENV90372.1_carbapenemase], OXA-355[AGW83453.1_carbapenemase], OXA-356[AGW83454.1_carbapenemase], OXA-418[AIN56719.1_carbapenemase]
**286**	OXA-286[ENU21708.1_unknown], OXA-287[WP_032865068.1_unknown], OXA-288[ENX60508.1_unknown], OXA-291[ENW94121.1_unknown], OXA-292[ENX53612.1_unknown], OXA-293[WP_032877719.1_unknown], OXA-302[ENX18112.1_unknown], OXA-303[ENX56371.1_unknown], OXA-306[ENX38336.1_unknown], OXA-307[WP_032879257.1_unknown]
**294**	OXA-294[ENU93232.1_unknown], OXA-295[ENX19403.1_unknown], OXA-297[ENX09209.1_unknown], OXA-298[ENX40034.1_unknown]
**464**	OXA-464[KLE13731.1_unknown], OXA-491[KLE07522.1_unknown], OXA-493[AJC94664.1_unknown], OXA-518[AMR98919.1_unknown]
**single variants**	OXA-12[AAA83417.1_narrow], OXA-153[ALA99187.1_carbapenemase], OXA-157[ALA99191.1_carbapenemase], OXA-18[AAB58555.1_ESBL], OXA-198[ADT70779.1_unknown], OXA-209[AEM66528.1_unknown], OXA-266[ENV38192.1_unknown], OXA-279[ENU35234.1_unknown], OXA-29[CAC35728.1_narrow], OXA-296[ENU21024.1_unknown], OXA-299[ENV82218.1_unknown], OXA-308[ENV32314.1_unknown], OXA-347[AET35493.1_unknown], OXA-372[AIG22448.1_carbapenemase], OXA-45[CAD58780.1_ESBL], OXA-504[ALG65432.1_unknown], OXA-55[AAR03105.1_carbapenemase], OXA-85[ALQ43092.1_narrow], OXA-9[AAA98406.2_narrow]

* = OXA-group 24 is labeled as group 40 in this Publication and in [2], the NCBI submitting portal currently lists only OXA-24. OXA-24 and -40 are identical.
# = groups are merged, because they share a sequence similarity of 80% or greater (see Figure S4).

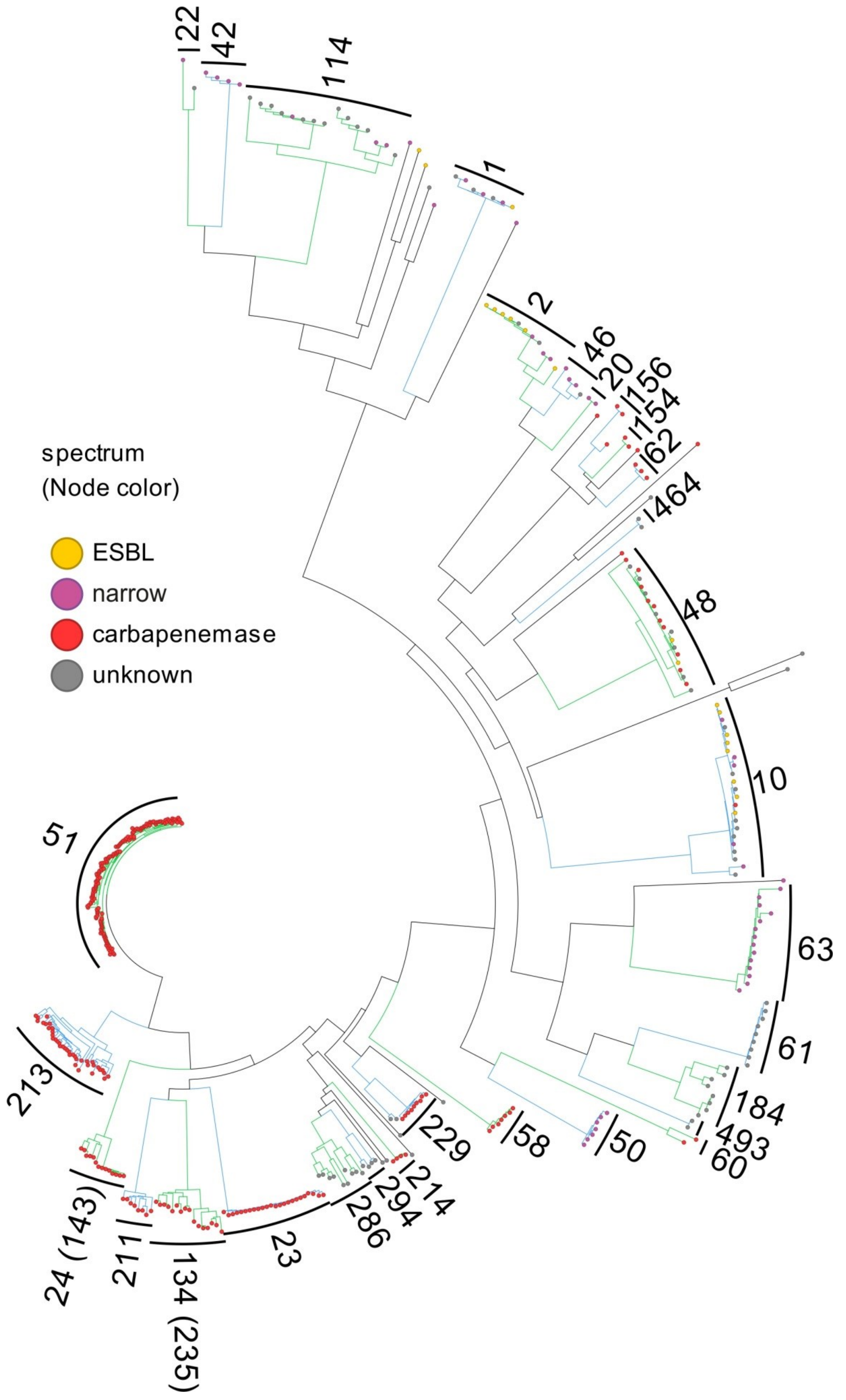

Supplementary Figure S5: Phylogenetic tree of 493 class D β-lactamases. Node color represents the respective phenotype; groups are labeled accordingly. Details on the variants of each group can be found in Table S4. Branched lines for each group are alternately colored in gray, green or blue for better visualization only.

Overview of the potential reservoir of Serine β-lactamases

Supplementary Table S5: Overview of class A clades. Data is based on GenBank entries and PubMed and refers to Figure 4. Selected publications related to the respective clade and brief descriptions of the context are included. All accession numbers and species are listed in Supplementary Dataset File SD1.

cluster	clade	reported phenotype	number of variants	identified nomenclatures	context
TEM/SHV	TEM	ESBL, narrow	436	TEM	Overview TEM[3]
	SHV/ LEN/ OKP	ESBL, narrow	458	SHV, LEN, OKP-A, OKP-B	SHV, LEN, OKP[4]
	other	ESBL, narrow	22	PLA, ORN, TER, LAP, GIL	β-lactamase characterization[5]
CARB	CARB I	carbenicillinase, narrow	134	CARB group 1, CARB group 17<br>VAK, VHW, VHH	soil resistome[6]; surface sea water[7]<br>VAK, VHW, VHH narrow [8,9]<br>CARB in clinical Pseudomonas[10]
	CARB II	ESBL, narrow	23	CKO, MAL, HER, HERA	CKO - ESBL[11]<br>HER – narrow [12]
	CARB III	ESBL, carbenicillinase	19	CARB group 5, RTG	genome report[13]; RTG [14]; CARB-5[15]
	other	carbenicillinase	50	AER, BRO, SCO, ROB	BRO[16] AER and CARB[17]
Carbapenemase/ESBL	penA/B	carbapenemase	35	penA, penB	β-lactamase in Burkholderia[18,19]; resistance mechanism[20]
	penI	ESBL	55	penI	tropical forest soil[21]; resistance mechanism[20,22]
	KPC/ IMI/ SME	carbapenemase	39	KPC, IMI, SME, SFC, BIC	BIC –Carbapenemase[23]<br>KPC Review [24]
	OXY	ESBL	60	OXY, K1	K1 – ESBL[25]; OXY – ESBL[26]
	FONA	ESBL	18	FONA, SFO	resistance spectrum [27]
	RAHN	ESBL	38	RAHN	resistance spectrum [28]
	CTX-M	ESBL	461	CTX-M subgroup 2, 8, 15 and 14<br>KLUC	CTX-M[29]<br>KLUC – ESBL[30]
	LUT	narrow	6	LUT	LUT[31]
	XIV		26	-	proteome analysis[32]
	other	ESBL	89	SED, PreB, LRG, HugA, ERP, BES	soil resistome[6]; genome report [33]; ESBL[34,35]; Alaska soil[36]<br>structure SED[37]; SED [38]
	blaA	ESBL	15	blaA	environmental samples or agricultural context[39]<br>blaA & blaB[40]<br>ESBL in *Erwinia persicina*[35]

**Gram+ clades**	BlaZ	narrow	146	-	blaZ in Staphylococcus aureus (16449305)
	VIII	narrow	386	OIH, penP	soil resistome[6]; livestock[41]; β-lactamase in bacillus[42] environmental samples or agricultural context [43]
	VII	narrow	187	-	β-lactamase in Streptomyces[44,45]; in Nocardia[46]
	VI	narrow	62	blaC (in Mycobacteria)	low level β-lactamase activity [47]
	V	narrow	14	AST-1(in Nocardia), FAR-1 (in Nocardia)	FAR-1[48]; AST-1[49]
	XII	n.s.	16	-	genome report[50]
	X	narrow	56	-	genome reports[51,52]
	IX	narrow	73	exo	β-lactamase in Streptomyces[45]
	other	n.s.	77	CAD, ACI	soil resistome[6], livestock[41]
**other**	I	n.s.	64	-	environmental samples or agricultural context[53,54]
	II	n.s.	51	-	environmental samples or agricultural context[55,56]
	III	narrow	141	Mainly L2 (in *Stenotrophomonas maltophilia*)	AmpR L2 module[57]; soil resistome[6] environmental samples or agricultural context [58,59]
	IV	n.s.	14	-	genome report[60]
	XI	n.s.	16	-	agricultural context[60]; genome report[61]
	XIII	n.s.	24	-	genome report[62]
	GES/ BEL	carbapenemase, ESBL	31	GES, BEL	GES – ESBL[63]; GES – Carbapenemase[64,65]; BEL – ESBL[66]
**A2 class**		ESBL	414	Amo, CblA, CepA, Cft, CfxA, CGA, CIA, CME, Crb, CSP, FRAMP, HGA, HGC, HGE, HGF, HGG, HOA, LRA, PER, TLA, VEB	Detailed description of class A2[67]

n.s. = not specified

Supplementary Table S6: Overview of AmpC clades. Data is based solely on GenBank entries and PubMed and refers to Figure 5. Selected publications related to the respective clade and brief descriptions of the context are included. All accession numbers and species are listed in Supplementary Dataset File SD2.

**cluster (plasmid)**	**ESBL reported**	**number of variants**	**identified names**	**context**
***Acinetobacter* I**	ESBL carbapenemase	229	ADC (up to 80)	clinical[68,69]; crystal structure [70]; carbapenemase[71]
***Acinetobacter* II**	n.s.	52	one tagged as ADC-8-like	-
***Aeromonas* (FOX, MOX)**	ESBL	68	AQU-1,2,3; CAV-1 MOX; FOX	AQU-1 [72]; CAV-1 [73]; FOX[74] clinical [75]
Agrobacterium	n.s.	37	-	plant pathogen [76]
Bradyrhizobium	n.s.	25	-	soybean symbiosis [55]
Burkholderia	n.s.	63	-	agricultural[77,78]; soil resistome[6]
Burkholderiaceae		14		
***Enterobacteriaceae* cluster (CMY, DHA, ACT/MIR)**	ESBL	672	AmpC-EC; AmpC-HG; CFE; CMY; ACT; MIR; DHA	AmpC Review[1]
***Enterobacteriaceae* II**	ESBL	72	SRT; SST; CSA; CMA	meropenem -resistant outbreak[79] CSA and CMA[80]; clinical[81] & resistance[82,83]
***Enterobacteriaceae* III (ACC)**	ESBL	12	ACC	clinical[84,85]; outbreak[86]
Laribacter	ESBL	8	-	ESBL[87]
Legionella	n.s.	16	-	-
Methylobacterium	n.s.	5	-	-
Mycobacterium	n.s.	8	-	-
Ochrobactrum	ESBL	17	OCH	resistance description [88,89]
***Pseudomonas* I**	ESBL	178	PDC (up to 40), Cft	soil [58,90]; cold adapted AmpC [91]
***Pseudomonas* II**	n.s.	39	-	genome report[92]
***Pseudomonas* III**	n.s.	61	-	genome report[93]
***Pseudomonas* IV**	n.s.	7	-	-
Rhizobiaceae	n.s.	34	-	soil resistome[6]
Rhizobium	n.s.	30	-	environment[53,54]
Shewanella	n.s.	15	-	-
Vibrionaceae & Moraxellaceae	ESBL	22	-	cold adapted AmpC[93]; genome report[94]
not assigned		162		

n.s. = not specified, - = nothing found

Supplementary Table S7: Overview of class D clades. Data is based solely on GenBank entries and PubMed and refers to Figure 6. Selected publications related to the respective clade and brief descriptions of the context are included. All accession numbers and species are listed in Supplementary Dataset File SD3.

cluster	OXA clade	number of variants found	reported phenotype	genus (species for Acinetobacter cluster)	context
**Acinetobacter**	**51**	167	carbapenemase	*A. baumannii*<br>*A. nosocomialis*	plasmid mediated OXA-51-like in *A. nosocomialis*[95]<br>clinical context[96]
	**213**	47	carbapenemase	*A. baumannii*<br>*A. calcoaceticus*<br>*A. oleivorans*<br>*A. pittii*	Genome characterization[97]
	**40*** (merged with clade 143)	30	carbapenemase	*A. baumannii*<br>*A. pittii*	OXA-143[98]<br>OXA-40 group[99]<br>OXA-72 in
	**23**	55	carbapenemase	*A. baumannii*<br>*A. radioresistens*<br>*A. calcoaceticus*<br>*K. pneumoniae*	carbapenem resistance in clinical isolates[100]
	**134** (merged with clade 235)	20	carbapenemase	*A. baumannii*<br>*A. lwoffii*<br>*A. schindleri*	OXA-235[101]<br>OXA-134-likes[102]
	**211**	8	carbapenemase	*A. johnsonii*	naturally occurring genes encoding carbapenem-hydrolysing oxacillinases[103]<br>resistance in ubiquitous bacteria[104]
	**214**	35	carbapenemase	*A. baumannii*<br>*A. bouvetii*<br>*A. gyllenbergii*<br>*A. haemolyticus*<br>*A. venetianus*	naturally occurring genes encoding carbapenem-hydrolysing oxacillinases[103]
	**229**	15	carbapenemase	*A. bohemicus*<br>*A. guillouiae*<br>*Caenorhabditis remanei*	characterization of OXA-229[103]
	**58**	15	carbapenemase	*A. baumannii*<br>*A. nosocomialis*	crystal structure[105]<br>clinical[106]
**D2 class**	**1**	29	ESBL	*Acinetobacter*<br>*Citrobacter*<br>*Escherichia*<br>*Klebsiella*<br>*Proteus*<br>*Pseudomonas*<br>*Salmonella*<br>*Shigella*	crystal structure [107]<br>characterization [108]
	**12**	38	narrow	*Aeromonas*	genome reports of *Aeromonas* [109,110]
	**22**	20	narrow	*Ralstonia*	OXA-22 [111]

	**42**	20	narrow	*Janthinobacterium*<br>*Variovorax*<br>*Burkholderia*	genomes [33]<br>resistance in *Burkholderia*[112]
	**114**	35	narrow	*Achromobacter*<br>*Pseudomonas*	*Achromobacter* in hospital, domestic and outdoor environmental samples [113]<br>Variants of OXA-114[114]
	**45**	3	ESBL	*Pseudomonas*	OXA-45 [115]
	**18**	6	ESBL	*Agrobacterium*	soil resistome [6]
	**9**	4	narrow	*Klebsiella*	Resistome analysis [116]
	**29**	20	narrow	*Fluoribacter*<br>*Legionella*	Metagenomic analysis of apple orchard soil [90]
**non clustered clades based on described OXA-variants**	**2**	32	ESBL	*Acinetobacter*<br>*Burkholderia*<br>*Klebsiella*<br>*Pseudomonas*<br>*Salmonella.*<br>*Stenotrophomonas*<br>*Thauera*	crystal structure [117]<br>βlactamases in aquatic environment [118]<br>ESBL[119]<br>clinical isolate of *Pseudomonas*[120]
	**20**	4	narrow	*Acinetobacter*	OXA-37 (20-like)[121]<br>β-lactamases in aquatic environment[118]
	**198**	11	carbapenemase	*Chlorobaculum*<br>*Chlorobium*<br>*Methylomonas*<br>*Pseudomonas*<br>*Thiocapsa*<br>*Thiocystis*<br>*Thiorhodococcus*<br>*Thiorhodovibrio*	OXA-198[122]
	**10**	49	ESBL<br>(one Carbapenemase report, not published)	*Acinetobacter*<br>*Citrobacter*<br>*Enterobacter*<br>*Escherichia*<br>*Klebsiella*<br>*Pseudomonas*<br>*Salmonella*<br>*Vibrio*	ESBL[123]<br>carbapenemase OXA-233 is unpublished<br>crystal structure[124]<br>Evolution to carbapenem-hydrolyzing activity by protein design[125]
	**48**	26	carbapenemase	*Enterobacter*<br>*Escherichia*<br>*Klebsiella*<br>*Shewanella*	spread of OXA-48[126]<br>characterization of carbapenemases[127,128]<br>crystal structure[129]
	**50**	47	narrow	*Pseudomonas*	clinical isolates[130]<br>OXA-50 in *P. aeruginosa*[131]<br>characterization[132]
	**62**	6	carbapenemase	*Pandoraea*	OXA-62[133]
	**60**	7	carbapenemase	*Ralstonia*	OXA-60[134]
	**184**	7	unknown	*Campylobacter*	-
	**61**	17	narrow	*Campylobacter*<br>*Escherichia*	resistance in *Campylobacter*[135]

	**63**	6	narrow	*Brachyspira*	resistance in *Brachyspira*[136,137]
	**85**	2	narrow	*Fusobacterium*	OXA-85[138]
	**209**	4	-	*Riemerella*	OXA-209 in *Riemerella* [139]
	**55**	5	-	Shewanella	OXA-55[140]

n.s. = not specified, * OXA-40 or labelled as OXA-24 (same sequence)

Literature of the Supplementary Information

1. Jacoby, G.A. AmpC beta-lactamases. *Clin Microbiol Rev* **22**, 161-82, Table of Contents (2009).
2. Evans, B.A. & Amyes, S.G. OXA beta-lactamases. *Clin Microbiol Rev* **27**, 241-63 (2014).
3. Salverda, M.L., De Visser, J.A. & Barlow, M. Natural evolution of TEM-1 beta-lactamase: experimental reconstruction and clinical relevance. *FEMS Microbiol Rev* **34**, 1015-36 (2010).
4. Haeggman, S., Lofdahl, S., Paauw, A., Verhoef, J. & Brisse, S. Diversity and evolution of the class A chromosomal beta-lactamase gene in Klebsiella pneumoniae. *Antimicrob Agents Chemother* **48**, 2400-8 (2004).
5. Walckenaer, E., Poirel, L., Leflon-Guibout, V., Nordmann, P. & Nicolas-Chanoine, M.H. Genetic and biochemical characterization of the chromosomal class A beta-lactamases of Raoultella (formerly Klebsiella) planticola and Raoultella ornithinolytica. *Antimicrob Agents Chemother* **48**, 305-12 (2004).
6. Forsberg, K.J. *et al.* Bacterial phylogeny structures soil resistomes across habitats. *Nature* **509**, 612-6 (2014).
7. Li, G., Lai, Q., Liu, X., Sun, F. & Shao, Z. Roseivivax atlanticus sp. nov., isolated from surface seawater of the Atlantic Ocean. *Antonie Van Leeuwenhoek* **105**, 863-9 (2014).
8. Jun, L.J., Kim, J.H., Jin, J.W. & Jeong, H.D. Characterization of a new beta-lactamase gene from isolates of Vibrio spp. in Korea. *J Microbiol Biotechnol* **22**, 555-62 (2012).
9. Teo, J.W., Suwanto, A. & Poh, C.L. Novel beta-lactamase genes from two environmental isolates of Vibrio harveyi. *Antimicrob Agents Chemother* **44**, 1309-14 (2000).
10. Wang, J., Bo, R., Xu, L., Mi, Z. & Wang, C. A CARB-like beta-lactamase gene from a multiple-drug-resistant Pseudomonas aeruginosa clinical isolate in China. *J Med Microbiol* **55**, 1609-10 (2006).
11. Petrella, S. *et al.* Characterization of the chromosomal class A beta-lactamase CKO from Citrobacter koseri. *FEMS Microbiol Lett* **254**, 285-92 (2006).
12. Beauchef-Havard, A. *et al.* Molecular and biochemical characterization of a novel class A beta-lactamase (HER-1) from Escherichia hermannii. *Antimicrob Agents Chemother* **47**, 2669-73 (2003).
13. Ivanova, E.P. *et al.* Draft Genome Sequences of Marinobacter similis A3d10T and Marinobacter salarius R9SW1T. *Genome Announc* **2**(2014).
14. Bonnin, R.A., Poirel, L. & Nordmann, P. A novel and hybrid composite transposon at the origin of acquisition of bla(RTG-5) in Acinetobacter baumannii. *Int J Antimicrob Agents* **40**, 257-9 (2012).
15. Paul, G., Joly-Guillou, M.L., Bergogne-Berezin, E., Nevot, P. & Philippon, A. Novel carbenicillin-hydrolyzing beta-lactamase (CARB-5) from Acinetobacter calcoaceticus var. anitratus. *FEMS Microbiol Lett* **50**, 45-50 (1989).
16. Bootsma, H.J., van Dijk, H., Verhoef, J., Fleer, A. & Mooi, F.R. Molecular characterization of the BRO beta-lactamase of Moraxella (Branhamella) catarrhalis. *Antimicrob Agents Chemother* **40**, 966-72 (1996).
17. Sanschagrin, F., Bejaoui, N. & Levesque, R.C. Structure of CARB-4 and AER-1 carbenicillin-hydrolyzing beta-lactamases. *Antimicrob Agents Chemother* **42**, 1966-72 (1998).
18. Poirel, L., Rodriguez-Martinez, J.M., Plesiat, P. & Nordmann, P. Naturally occurring Class A ss-lactamases from the Burkholderia cepacia complex. *Antimicrob Agents Chemother* **53**, 876-82 (2009).
19. Tribuddharat, C., Moore, R.A., Baker, P. & Woods, D.E. Burkholderia pseudomallei class a beta-lactamase mutations that confer selective resistance against ceftazidime or clavulanic acid inhibition. *Antimicrob Agents Chemother* **47**, 2082-7 (2003).
20. Papp-Wallace, K.M. *et al.* Insights into beta-lactamases from Burkholderia species, two phylogenetically related yet distinct resistance determinants. *J Biol Chem* **288**, 19090-102 (2013).
21. Woo, H.L. *et al.* Draft Genome Sequence of the Lignin-Degrading Burkholderia sp. Strain LIG30, Isolated from Wet Tropical Forest Soil. *Genome Announc* **2**(2014).

22. Sarovich, D.S. *et al.* Characterization of ceftazidime resistance mechanisms in clinical isolates of Burkholderia pseudomallei from Australia. *PLoS One* **7**, e30789 (2012).
23. Girlich, D., Poirel, L. & Nordmann, P. Novel ambler class A carbapenem-hydrolyzing beta-lactamase from a Pseudomonas fluorescens isolate from the Seine River, Paris, France. *Antimicrob Agents Chemother* **54**, 328-32 (2010).
24. De Rosa, F.G., Corcione, S., Cavallo, R., Di Perri, G. & Bassetti, M. Critical issues for Klebsiella pneumoniae KPC-carbapenemase producing K. pneumoniae infections: a critical agenda. *Future Microbiol* **10**, 283-94 (2015).
25. Granier, S.A., Leflon-Guibout, V., Nicolas-Chanoine, M.H., Bush, K. & Goldstein, F.W. The extended-spectrum K1 beta-lactamase from Klebsiella oxytoca SC 10,436 is a member of the bla(OXY-2) family of chromosomal Klebsiella enzymes. *Antimicrob Agents Chemother* **46**, 2056-7 (2002).
26. Liang, Y.H., Gao, R. & Su, X.D. Structural insights into the broadened substrate profile of the extended-spectrum beta-lactamase OXY-1-1 from Klebsiella oxytoca. *Acta Crystallogr D Biol Crystallogr* **68**, 1460-7 (2012).
27. Blaak, H. *et al.* Extended spectrum ss-lactamase- and constitutively AmpC-producing Enterobacteriaceae on fresh produce and in the agricultural environment. *Int J Food Microbiol* **168-169**, 8-16 (2014).
28. Tacao, M., Correia, A. & Henriques, I. Resistance to broad-spectrum antibiotics in aquatic systems: anthropogenic activities modulate the dissemination of bla(CTX-M)-like genes. *Appl Environ Microbiol* **78**, 4134-40 (2012).
29. Lahlaoui, H., Ben Haj Khalifa, A. & Ben Moussa, M. Epidemiology of Enterobacteriaceae producing CTX-M type extended spectrum beta-lactamase (ESBL). *Med Mal Infect* **44**, 400-4 (2014).
30. Xu, T. *et al.* Identification and characterization of two novel bla(KLUC) resistance genes through large-scale resistance plasmids sequencing. *PLoS One* **7**, e47197 (2012).
31. Doublet, B. *et al.* Molecular and biochemical characterization of the natural chromosome-encoded class A beta-lactamase from Pseudomonas luteola. *Antimicrob Agents Chemother* **54**, 45-51 (2010).
32. Davies, D.H. *et al.* Profiling the humoral immune response to infection by using proteome microarrays: high-throughput vaccine and diagnostic antigen discovery. *Proc Natl Acad Sci U S A* **102**, 547-52 (2005).
33. Gan, H.Y. *et al.* Whole-genome sequences of 13 endophytic bacteria isolated from shrub willow (salix) grown in geneva, new york. *Genome Announc* **2**(2014).
34. Lartigue, M.F. *et al.* Characterization of an extended-spectrum class A beta-lactamase from a novel enterobacterial species taxonomically related to Rahnella spp./Ewingella spp. *J Antimicrob Chemother* **68**, 1733-6 (2013).
35. Vimont, S., Poirel, L., Naas, T. & Nordmann, P. Identification of a chromosome-borne expanded-spectrum class a beta-lactamase from Erwinia persicina. *Antimicrob Agents Chemother* **46**, 3401-5 (2002).
36. Allen, H.K., Moe, L.A., Rodbumrer, J., Gaarder, A. & Handelsman, J. Functional metagenomics reveals diverse beta-lactamases in a remote Alaskan soil. *ISME J* **3**, 243-51 (2009).
37. Petrella, S., Pernot, L. & Sougakoff, W. Crystallization and preliminary X-ray diffraction study of the class A beta-lactamase SED-1 and its mutant SED-G238C from Citrobacter sedlakii. *Acta Crystallogr D Biol Crystallogr* **60**, 125-8 (2004).
38. Petrella, S., Clermont, D., Casin, I., Jarlier, V. & Sougakoff, W. Novel class A beta-lactamase Sed-1 from Citrobacter sedlakii: genetic diversity of beta-lactamases within the Citrobacter genus. *Antimicrob Agents Chemother* **45**, 2287-98 (2001).
39. Ghazal, S. *et al.* Draft Genome Sequence of Photorhabdus luminescens Strain BA1, an Entomopathogenic Bacterium Isolated from Nematodes Found in Egypt. *Genome Announc* **2**(2014).
40. Mittal, S., Mallik, S., Sharma, S. & Virdi, J.S. Characteristics of beta-lactamases and their genes (blaA and blaB) in Yersinia intermedia and Y. frederiksenii. *BMC Microbiol* **7**, 25 (2007).

41. Wichmann, F., Udikovic-Kolic, N., Andrew, S. & Handelsman, J. Diverse antibiotic resistance genes in dairy cow manure. *MBio* **5**, e01017 (2014).
42. Hussain, M., Pastor, F.I. & Lampen, J.O. Cloning and sequencing of the blaZ gene encoding beta-lactamase III, a lipoprotein of Bacillus cereus 569/H. *J Bacteriol* **169**, 579-86 (1987).
43. Wang, Y., Wang, X., Greenfield, P., Jin, D. & Bai, Z. Draft Genome Sequence of Bacillus amyloliquefaciens EBL11, a New Strain of Plant Growth-Promoting Bacterium Isolated from Rice Rhizosphere. *Genome Announc* **2**(2014).
44. Hoshino, Y., Nakamori, S. & Takagi, H. Cloning and analysis of the beta-lactamase gene from epsilon-poly-L-lysine-producing actinomycete Streptomyces albulus IFO14147. *J Biochem* **134**, 473-8 (2003).
45. Forsman, M., Haggstrom, B., Lindgren, L. & Jaurin, B. Molecular analysis of beta-lactamases from four species of Streptomyces: comparison of amino acid sequences with those of other beta-lactamases. *J Gen Microbiol* **136**, 589-98 (1990).
46. Coque, J.J., Liras, P. & Martin, J.F. Genes for a beta-lactamase, a penicillin-binding protein and a transmembrane protein are clustered with the cephamycin biosynthetic genes in Nocardia lactamdurans. *EMBO J* **12**, 631-9 (1993).
47. Perez-Llarena, F. *et al.* The bla gene of the cephamycin cluster of Streptomyces clavuligerus encodes a class A beta-lactamase of low enzymatic activity. *J Bacteriol* **179**, 6035-40 (1997).
48. Laurent, F. *et al.* Biochemical-genetic analysis and distribution of FAR-1, a class A beta-lactamase from Nocardia farcinica. *Antimicrob Agents Chemother* **43**, 1644-50 (1999).
49. Poirel, L. *et al.* Molecular and biochemical analysis of AST-1, a class A beta-lactamase from Nocardia asteroides sensu stricto. *Antimicrob Agents Chemother* **45**, 878-82 (2001).
50. Harjes, J. *et al.* Draft Genome Sequence of the Antitrypanosomally Active Sponge-Associated Bacterium Actinokineospora sp. Strain EG49. *Genome Announc* **2**(2014).
51. Kwak, Y., Park, G.S., Lee, S.E., Li, Q.X. & Shin, J.H. Genome sequence of Mycobacterium aromaticivorans JS19b1(T), a novel isolate from Hawaiian soil. *J Biotechnol* **186**, 137-8 (2014).
52. Croce, O., Robert, C., Raoult, D. & Drancourt, M. Draft Genome Sequence of Mycobacterium farcinogenes NCTC 10955. *Genome Announc* **2**(2014).
53. Fauvart, M., Sanchez-Rodriguez, A., Beullens, S., Marchal, K. & Michiels, J. Genome sequence of Rhizobium etli CNPAF512, a nitrogen-fixing symbiont isolated from bean root nodules in Brazil. *J Bacteriol* **193**, 3158-9 (2011).
54. Mora, Y. *et al.* Nitrogen-fixing rhizobial strains isolated from common bean seeds: phylogeny, physiology, and genome analysis. *Appl Environ Microbiol* **80**, 5644-54 (2014).
55. Siqueira, A.F. *et al.* Comparative genomics of Bradyrhizobium japonicum CPAC 15 and Bradyrhizobium diazoefficiens CPAC 7: elite model strains for understanding symbiotic performance with soybean. *BMC Genomics* **15**, 420 (2014).
56. Servin-Garciduenas, L.E. *et al.* Symbiont shift towards Rhizobium nodulation in a group of phylogenetically related Phaseolus species. *Mol Phylogenet Evol* **79**, 1-11 (2014).
57. Chang, Y.C., Huang, Y.W., Chiang, K.H., Yang, T.C. & Chung, T.C. Introduction of an AmpR-L2 intergenic segment attenuates the induced beta-lactamase activity of Stenotrophomonas maltophilia. *Eur J Clin Microbiol Infect Dis* **29**, 887-90 (2010).
58. Perron, G.G. *et al.* Functional characterization of bacteria isolated from ancient arctic soil exposes diverse resistance mechanisms to modern antibiotics. *PLoS One* **10**, e0069533 (2015).
59. Puopolo, G., Sonego, P., Engelen, K. & Pertot, I. Draft Genome Sequence of Lysobacter capsici AZ78, a Bacterium Antagonistic to Plant-Pathogenic Oomycetes. *Genome Announc* **2**(2014).
60. Register, K.B. *et al.* Draft Genome Sequences of 53 Genetically Distinct Isolates of Bordetella bronchiseptica Representing 11 Terrestrial and Aquatic Hosts. *Genome Announc* **3**(2015).
61. Okada, K. *et al.* Complete Genome Sequence of Bordetella bronchiseptica S798, an Isolate from a Pig with Atrophic Rhinitis. *Genome Announc* **2**(2014).
62. Adriaenssens, E.M., Guerrero, L.D., Makhalanyane, T.P., Aislabie, J.M. & Cowan, D.A. Draft Genome Sequence of the Aromatic Hydrocarbon-Degrading Bacterium Sphingobium sp. Strain Ant17, Isolated from Antarctic Soil. *Genome Announc* **2**(2014).

63. Delbruck, H. *et al.* Kinetic and crystallographic studies of extended-spectrum GES-11, GES-12, and GES-14 beta-lactamases. *Antimicrob Agents Chemother* **56**, 5618-25 (2012).
64. Vourli, S. *et al.* Novel GES/IBC extended-spectrum beta-lactamase variants with carbapenemase activity in clinical enterobacteria. *FEMS Microbiol Lett* **234**, 209-13 (2004).
65. Poirel, L. *et al.* GES-2, a class A beta-lactamase from Pseudomonas aeruginosa with increased hydrolysis of imipenem. *Antimicrob Agents Chemother* **45**, 2598-603 (2001).
66. Poirel, L. *et al.* BEL-2, an extended-spectrum beta-lactamase with increased activity toward expanded-spectrum cephalosporins in Pseudomonas aeruginosa. *Antimicrob Agents Chemother* **54**, 533-5 (2010).
67. Philippon, A., Slama, P., Deny, P. & Labia, R. A Structure-Based Classification of Class A beta-Lactamases, a Broadly Diverse Family of Enzymes. *Clin Microbiol Rev* **29**, 29-57 (2016).
68. Zhou, Y., Teng, S.J., Yang, L., Li, S.B. & Xu, Y. A novel variant of the beta-lactamase ADC-61 gene in multi-drug resistant Acinetobacter baumannii. *Genet Mol Res* **14**, 7092-100 (2015).
69. Dent, L.L., Marshall, D.R., Pratap, S. & Hulette, R.B. Multidrug resistant Acinetobacter baumannii: a descriptive study in a city hospital. *BMC Infect Dis* **10**, 196 (2010).
70. Bhattacharya, M., Toth, M., Antunes, N.T., Smith, C.A. & Vakulenko, S.B. Structure of the extended-spectrum class C beta-lactamase ADC-1 from Acinetobacter baumannii. *Acta Crystallogr D Biol Crystallogr* **70**, 760-71 (2014).
71. Jeon, J.H. *et al.* Structure of ADC-68, a novel carbapenem-hydrolyzing class C extended-spectrum beta-lactamase isolated from Acinetobacter baumannii. *Acta Crystallogr D Biol Crystallogr* **70**, 2924-36 (2014).
72. Wu, C.J. *et al.* AQU-1, a chromosomal class C beta-lactamase, among clinical Aeromonas dhakensis isolates: distribution and clinical significance. *Int J Antimicrob Agents* **42**, 456-61 (2013).
73. Fosse, T., Giraud-Morin, C., Madinier, I. & Labia, R. Sequence analysis and biochemical characterisation of chromosomal CAV-1 (Aeromonas caviae), the parental cephalosporinase of plasmid-mediated AmpC 'FOX' cluster. *FEMS Microbiol Lett* **222**, 93-8 (2003).
74. Bou, G., Oliver, A., Ojeda, M., Monzon, C. & Martinez-Beltran, J. Molecular characterization of FOX-4, a new AmpC-type plasmid-mediated beta-lactamase from an Escherichia coli strain isolated in Spain. *Antimicrob Agents Chemother* **44**, 2549-53 (2000).
75. Ye, Y., Xu, X.H. & Li, J.B. Emergence of CTX-M-3, TEM-1 and a new plasmid-mediated MOX-4 AmpC in a multiresistant Aeromonas caviae isolate from a patient with pneumonia. *J Med Microbiol* **59**, 843-7 (2010).
76. Henkel, C.V., den Dulk-Ras, A., Zhang, X. & Hooykaas, P.J. Genome Sequence of the Octopine-Type Agrobacterium tumefaciens Strain Ach5. *Genome Announc* **2**(2014).
77. Khan, A., Asif, H., Studholme, D.J., Khan, I.A. & Azim, M.K. Genome characterization of a novel Burkholderia cepacia complex genomovar isolated from dieback affected mango orchards. *World J Microbiol Biotechnol* **29**, 2033-44 (2013).
78. Takeshita, K. *et al.* Whole-Genome Sequence of Burkholderia sp. Strain RPE67, a Bacterial Gut Symbiont of the Bean Bug Riptortus pedestris. *Genome Announc* **2**(2014).
79. Suh, B. *et al.* Outbreak of meropenem-resistant Serratia marcescens comediated by chromosomal AmpC beta-lactamase overproduction and outer membrane protein loss. *Antimicrob Agents Chemother* **54**, 5057-61 (2010).
80. Muller, A., Hachler, H., Stephan, R. & Lehner, A. Presence of AmpC beta-lactamases, CSA-1, CSA-2, CMA-1, and CMA-2 conferring an unusual resistance phenotype in Cronobacter sakazakii and Cronobacter malonaticus. *Microb Drug Resist* **20**, 275-80 (2014).
81. Crivaro, V. *et al.* Risk factors for extended-spectrum beta-lactamase-producing Serratia marcescens and Klebsiella pneumoniae acquisition in a neonatal intensive care unit. *J Hosp Infect* **67**, 135-41 (2007).
82. Raimondi, A., Sisto, F. & Nikaido, H. Mutation in Serratia marcescens AmpC beta-lactamase producing high-level resistance to ceftazidime and cefpirome. *Antimicrob Agents Chemother* **45**, 2331-9 (2001).

83. Hidri, N. *et al.* Resistance to ceftazidime is associated with a S220Y substitution in the omega loop of the AmpC beta-lactamase of a Serratia marcescens clinical isolate. *J Antimicrob Chemother* **55**, 496-9 (2005).
84. Roche, C., Boo, T.W., Walsh, F. & Crowley, B. Detection and molecular characterisation of plasmidic AmpC beta-lactamases in Klebsiella pneumoniae isolates from a tertiary-care hospital in Dublin, Ireland. *Clin Microbiol Infect* **14**, 616-8 (2008).
85. Bauernfeind, A., Schneider, I., Jungwirth, R., Sahly, H. & Ullmann, U. A novel type of AmpC beta-lactamase, ACC-1, produced by a Klebsiella pneumoniae strain causing nosocomial pneumonia. *Antimicrob Agents Chemother* **43**, 1924-31 (1999).
86. Nadjar, D. *et al.* Outbreak of Klebsiella pneumoniae producing transferable AmpC-type beta-lactamase (ACC-1) originating from Hafnia alvei. *FEMS Microbiol Lett* **187**, 35-40 (2000).
87. Lau, S.K. *et al.* Cloning and characterization of a chromosomal class C beta-lactamase and its regulatory gene in Laribacter hongkongensis. *Antimicrob Agents Chemother* **49**, 1957-64 (2005).
88. Nadjar, D. *et al.* Molecular characterization of chromosomal class C beta-lactamase and its regulatory gene in Ochrobactrum anthropi. *Antimicrob Agents Chemother* **45**, 2324-30 (2001).
89. Higgins, C.S. *et al.* Characterization, cloning and sequence analysis of the inducible Ochrobactrum anthropi AmpC beta-lactamase. *J Antimicrob Chemother* **47**, 745-54 (2001).
90. Donato, J.J. *et al.* Metagenomic analysis of apple orchard soil reveals antibiotic resistance genes encoding predicted bifunctional proteins. *Appl Environ Microbiol* **76**, 4396-401 (2010).
91. Michaux, C. *et al.* Crystal structure of a cold-adapted class C beta-lactamase. *FEBS J* **275**, 1687-97 (2008).
92. Hu, X. *et al.* Complete Genome Sequence of the p-Nitrophenol-Degrading Bacterium Pseudomonas putida DLL-E4. *Genome Announc* **2**(2014).
93. Feller, G., Zekhnini, Z., Lamotte-Brasseur, J. & Gerday, C. Enzymes from cold-adapted microorganisms. The class C beta-lactamase from the antarctic psychrophile Psychrobacter immobilis A5. *Eur J Biochem* **244**, 186-91 (1997).
94. Machado, H., Mansson, M. & Gram, L. Draft Genome Sequence of Photobacterium halotolerans S2753, Producer of Bioactive Secondary Metabolites. *Genome Announc* **2**(2014).
95. Lee, Y.T. *et al.* Emergence of carbapenem-resistant non-baumannii species of Acinetobacter harboring a blaOXA-51-like gene that is intrinsic to A. baumannii. *Antimicrob Agents Chemother* **56**, 1124-7 (2012).
96. Evans, B.A., Brown, S., Hamouda, A., Findlay, J. & Amyes, S.G. Eleven novel OXA-51-like enzymes from clinical isolates of Acinetobacter baumannii. *Clin Microbiol Infect* **13**, 1137-8 (2007).
97. Ketter, P. *et al.* Genome Sequences of Four Acinetobacter baumannii-A. calcoaceticus Complex Isolates from Combat-Related Infections Sustained in the Middle East. *Genome Announc* **2**(2014).
98. Zander, E., Bonnin, R.A., Seifert, H. & Higgins, P.G. Characterization of blaOXA-143 variants in Acinetobacter baumannii and Acinetobacter pittii. *Antimicrob Agents Chemother* **58**, 2704-8 (2014).
99. Tian, G.B. *et al.* Identification of diverse OXA-40 group carbapenemases, including a novel variant, OXA-160, from Acinetobacter baumannii in Pennsylvania. *Antimicrob Agents Chemother* **55**, 429-32 (2011).
100. Afzal-Shah, M., Woodford, N. & Livermore, D.M. Characterization of OXA-25, OXA-26, and OXA-27, molecular class D beta-lactamases associated with carbapenem resistance in clinical isolates of Acinetobacter baumannii. *Antimicrob Agents Chemother* **45**, 583-8 (2001).
101. Higgins, P.G. *et al.* OXA-235, a novel class D beta-lactamase involved in resistance to carbapenems in Acinetobacter baumannii. *Antimicrob Agents Chemother* **57**, 2121-6 (2013).
102. Turton, J.F., Hyde, R., Martin, K. & Shah, J. Genes encoding OXA-134-like enzymes are found in Acinetobacter lwoffii and A. schindleri and can be used for identification. *J Clin Microbiol* **50**, 1019-22 (2012).

103. Figueiredo, S., Bonnin, R.A., Poirel, L., Duranteau, J. & Nordmann, P. Identification of the naturally occurring genes encoding carbapenem-hydrolysing oxacillinases from Acinetobacter haemolyticus, Acinetobacter johnsonii, and Acinetobacter calcoaceticus. *Clin Microbiol Infect* **18**, 907-13 (2012).
104. Manageiro, V., Ferreira, E., Canica, M. & Manaia, C.M. GES-5 among the beta-lactamases detected in ubiquitous bacteria isolated from aquatic environment samples. *FEMS Microbiol Lett* **351**, 64-69 (2014).
105. Smith, C.A., Antunes, N.T., Toth, M. & Vakulenko, S.B. Crystal structure of carbapenemase OXA-58 from Acinetobacter baumannii. *Antimicrob Agents Chemother* **58**, 2135-43 (2014).
106. Poirel, L., Mansour, W., Bouallegue, O. & Nordmann, P. Carbapenem-resistant Acinetobacter baumannii isolates from Tunisia producing the OXA-58-like carbapenem-hydrolyzing oxacillinase OXA-97. *Antimicrob Agents Chemother* **52**, 1613-7 (2008).
107. Sun, T. *et al.* Crystallization and preliminary X-ray study of OXA-1, a class D beta-lactamase. *Acta Crystallogr D Biol Crystallogr* **57**, 1912-4 (2001).
108. Lina, T.T. *et al.* Phenotypic and molecular characterization of extended-spectrum beta-lactamase-producing Escherichia coli in Bangladesh. *PLoS One* **9**, e108735 (2014).
109. Chua, P. *et al.* Genome sequencing and annotation of Aeromonas sp. HZM. *Genom Data* **5**, 38-9 (2015).
110. Lenneman, E.M. & Barney, B.M. Draft Genome Sequences of the Alga-Degrading Bacteria Aeromonas hydrophila Strain AD9 and Pseudomonas pseudoalcaligenes Strain AD6. *Genome Announc* **2**(2014).
111. Nordmann, P., Poirel, L., Kubina, M., Casetta, A. & Naas, T. Biochemical-genetic characterization and distribution of OXA-22, a chromosomal and inducible class D beta-lactamase from Ralstonia (Pseudomonas) pickettii. *Antimicrob Agents Chemother* **44**, 2201-4 (2000).
112. Niumsup, P. & Wuthiekanun, V. Cloning of the class D beta-lactamase gene from Burkholderia pseudomallei and studies on its expression in ceftazidime-susceptible and -resistant strains. *J Antimicrob Chemother* **50**, 445-55 (2002).
113. Amoureux, L. *et al.* Detection of Achromobacter xylosoxidans in hospital, domestic, and outdoor environmental samples and comparison with human clinical isolates. *Appl Environ Microbiol* **79**, 7142-9 (2013).
114. Traglia, G.M. *et al.* Distribution of allelic variants of the chromosomal gene bla OXA-114-like in Achromobacter xylosoxidans clinical isolates. *Curr Microbiol* **67**, 596-600 (2013).
115. Toleman, M.A., Rolston, K., Jones, R.N. & Walsh, T.R. Molecular and biochemical characterization of OXA-45, an extended-spectrum class 2d' beta-lactamase in Pseudomonas aeruginosa. *Antimicrob Agents Chemother* **47**, 2859-63 (2003).
116. Lee, Y. *et al.* Clonality and Resistome analysis of KPC-producing Klebsiella pneumoniae strain isolated in Korea using whole genome sequencing. *Biomed Res Int* **2014**, 352862 (2014).
117. Docquier, J.D. *et al.* Crystal structure of the narrow-spectrum OXA-46 class D beta-lactamase: relationship between active-site lysine carbamylation and inhibition by polycarboxylates. *Antimicrob Agents Chemother* **54**, 2167-74 (2010).
118. Henriques, I., Moura, A., Alves, A., Saavedra, M.J. & Correia, A. Analysing diversity among beta-lactamase encoding genes in aquatic environments. *FEMS Microbiol Ecol* **56**, 418-29 (2006).
119. Danel, F., Hall, L.M., Gur, D. & Livermore, D.M. OXA-15, an extended-spectrum variant of OXA-2 beta-lactamase, isolated from a Pseudomonas aeruginosa strain. *Antimicrob Agents Chemother* **41**, 785-90 (1997).
120. Juan, C. *et al.* Activity of a new antipseudomonal cephalosporin, CXA-101 (FR264205), against carbapenem-resistant and multidrug-resistant Pseudomonas aeruginosa clinical strains. *Antimicrob Agents Chemother* **54**, 846-51 (2010).
121. Navia, M.M., Ruiz, J. & Vila, J. Characterization of an integron carrying a new class D beta-lactamase (OXA-37) in Acinetobacter baumannii. *Microb Drug Resist* **8**, 261-5 (2002).

122. El Garch, F., Bogaerts, P., Bebrone, C., Galleni, M. & Glupczynski, Y. OXA-198, an acquired carbapenem-hydrolyzing class D beta-lactamase from Pseudomonas aeruginosa. *Antimicrob Agents Chemother* **55**, 4828-33 (2011).
123. Liu, W., Liu, X., Liao, J., Zhang, Y. & Liang, X. Identification of blaOXA-128 and blaOXA-129, two novel OXA-type extended-spectrum-beta-lactamases in Pseudomonas aeruginosa, in Hunan Province, China. *J Basic Microbiol* **50 Suppl 1**, S116-9 (2010).
124. Pernot, L. *et al.* Crystal structures of the class D beta-lactamase OXA-13 in the native form and in complex with meropenem. *J Mol Biol* **310**, 859-74 (2001).
125. De Luca, F. *et al.* Evolution to carbapenem-hydrolyzing activity in noncarbapenemase class D beta-lactamase OXA-10 by rational protein design. *Proc Natl Acad Sci U S A* **108**, 18424-9 (2011).
126. Fursova, N.K. *et al.* The spread of bla OXA-48 and bla OXA-244 carbapenemase genes among Klebsiella pneumoniae, Proteus mirabilis and Enterobacter spp. isolated in Moscow, Russia. *Ann Clin Microbiol Antimicrob* **14**, 46 (2015).
127. Potron, A., Nordmann, P. & Poirel, L. Characterization of OXA-204, a carbapenem-hydrolyzing class D beta-lactamase from Klebsiella pneumoniae. *Antimicrob Agents Chemother* **57**, 633-6 (2013).
128. Potron, A. *et al.* Genetic and biochemical characterisation of OXA-232, a carbapenem-hydrolysing class D beta-lactamase from Enterobacteriaceae. *Int J Antimicrob Agents* **41**, 325-9 (2013).
129. Docquier, J.D. *et al.* Crystal structure of the OXA-48 beta-lactamase reveals mechanistic diversity among class D carbapenemases. *Chem Biol* **16**, 540-7 (2009).
130. Martin, K. *et al.* Clusters of genetically similar isolates of Pseudomonas aeruginosa from multiple hospitals in the UK. *J Med Microbiol* **62**, 988-1000 (2013).
131. Empel, J. *et al.* Outbreak of Pseudomonas aeruginosa infections with PER-1 extended-spectrum beta-lactamase in Warsaw, Poland: further evidence for an international clonal complex. *J Clin Microbiol* **45**, 2829-34 (2007).
132. Kong, K.F. *et al.* Characterization of poxB, a chromosomal-encoded Pseudomonas aeruginosa oxacillinase. *Gene* **358**, 82-92 (2005).
133. Schneider, I., Queenan, A.M. & Bauernfeind, A. Novel carbapenem-hydrolyzing oxacillinase OXA-62 from Pandoraea pnomenusa. *Antimicrob Agents Chemother* **50**, 1330-5 (2006).
134. Girlich, D., Naas, T. & Nordmann, P. OXA-60, a chromosomal, inducible, and imipenem-hydrolyzing class D beta-lactamase from Ralstonia pickettii. *Antimicrob Agents Chemother* **48**, 4217-25 (2004).
135. Obeng, A.S. *et al.* Antimicrobial susceptibilities and resistance genes in Campylobacter strains isolated from poultry and pigs in Australia. *J Appl Microbiol* **113**, 294-307 (2012).
136. Jansson, D.S. & Pringle, M. Antimicrobial susceptibility of Brachyspira spp. isolated from commercial laying hens and free-living wild mallards (Anas platyrhynchos). *Avian Pathol* **40**, 387-93 (2011).
137. Mortimer-Jones, S.M., Phillips, N.D., La, T., Naresh, R. & Hampson, D.J. Penicillin resistance in the intestinal spirochaete Brachyspira pilosicoli associated with OXA-136 and OXA-137, two new variants of the class D beta-lactamase OXA-63. *J Med Microbiol* **57**, 1122-8 (2008).
138. Voha, C., Docquier, J.D., Rossolini, G.M. & Fosse, T. Genetic and biochemical characterization of FUS-1 (OXA-85), a narrow-spectrum class D beta-lactamase from Fusobacterium nucleatum subsp. polymorphum. *Antimicrob Agents Chemother* **50**, 2673-9 (2006).
139. Chen, Y.P., Lee, S.H., Chou, C.H. & Tsai, H.J. Detection of florfenicol resistance genes in Riemerella anatipestifer isolated from ducks and geese. *Vet Microbiol* **154**, 325-31 (2012).
140. Heritier, C., Poirel, L. & Nordmann, P. Genetic and biochemical characterization of a chromosome-encoded carbapenem-hydrolyzing ambler class D beta-lactamase from Shewanella algae. *Antimicrob Agents Chemother* **48**, 1670-5 (2004).

Supplementary Figure S6

High resolution tree of Manuskript Figure 2

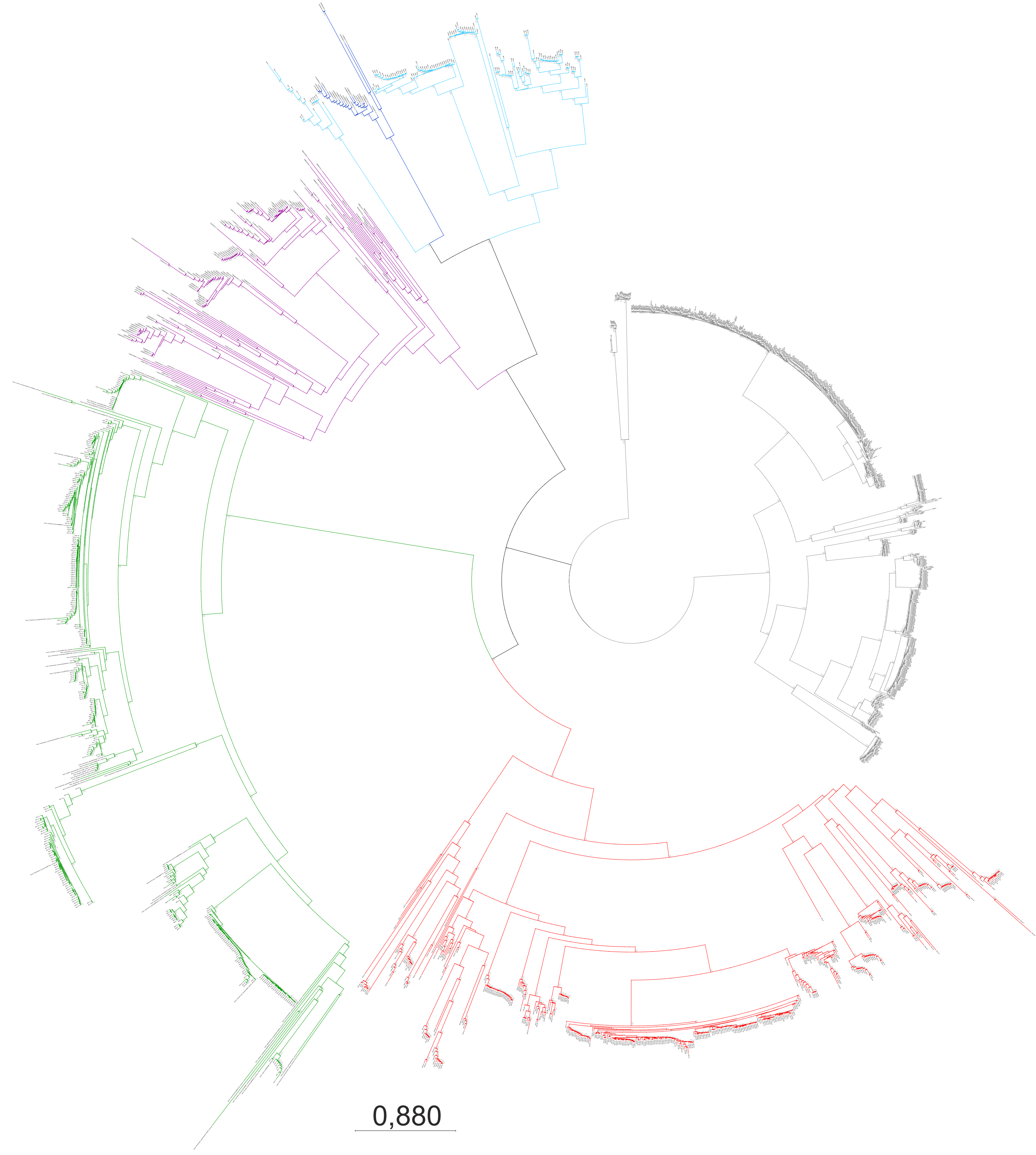
0,880

Supplementary Figure S7

High resolution tree of Manuskript Figure 4

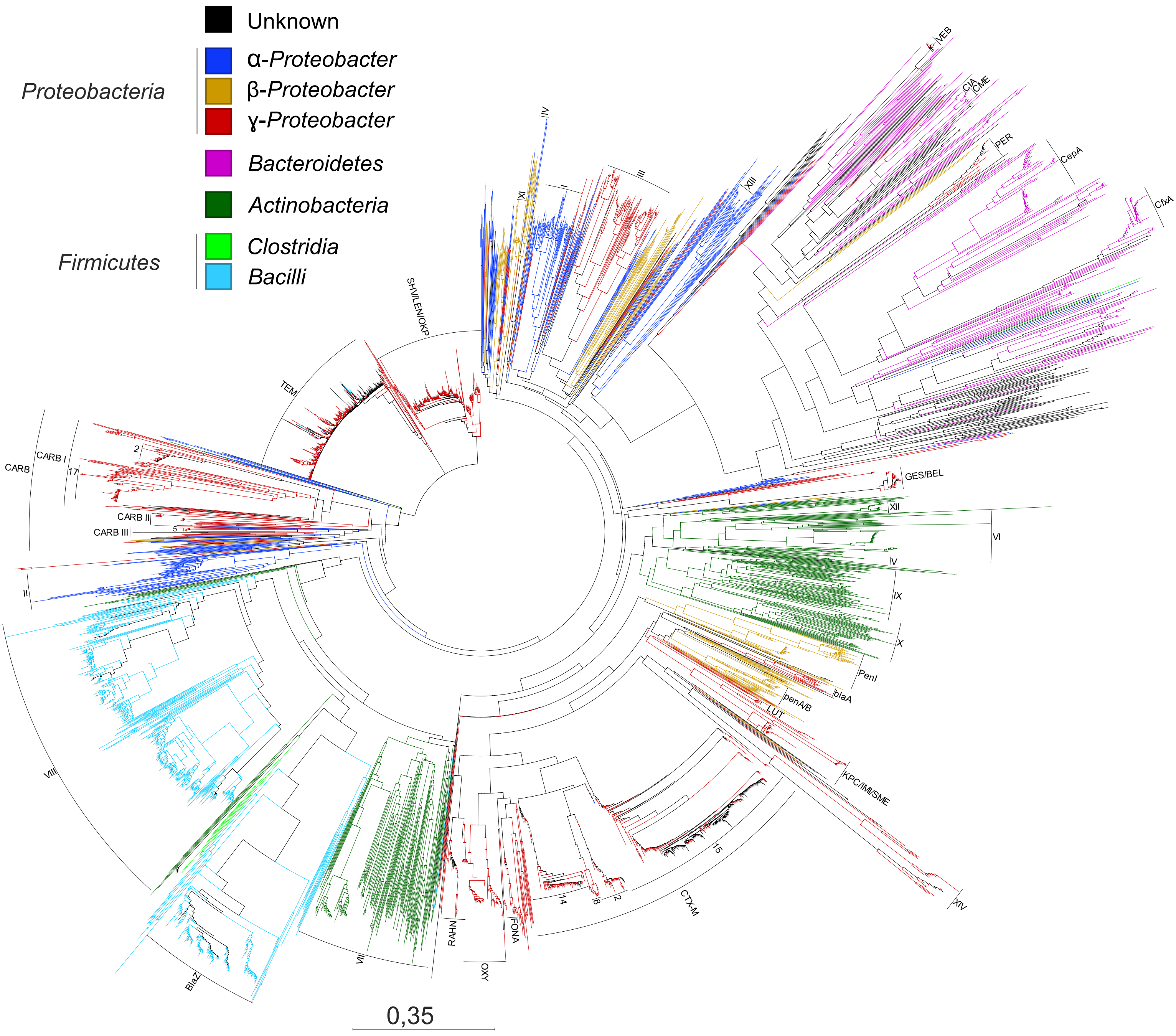
Unknown
Proteobacteria
α-Proteobacter
β-Proteobacter
ɣ-Proteobacter
Bacteroidetes
Actinobacteria
Firmicutes
Clostridia
Bacilli
VEB
CIA
CME
PER
CepA
CfxA
IV
III
XI
I
XIII
SHV/LEN/OKP
TEM
CARB
CARB I
2
17
CARB II
CARB III
5
II
GES/BEL
XII
VI
V
IX
X
PenI
blaA
penA/B
LUT
KPC/IMI/SME
XIV
VIII
15
CTX-M
14
8
2
FONA
RAHN
OXY
VII
BlaZ
0,35

Supplementary Figure S8

High resolution tree of Manuskript Figure 5

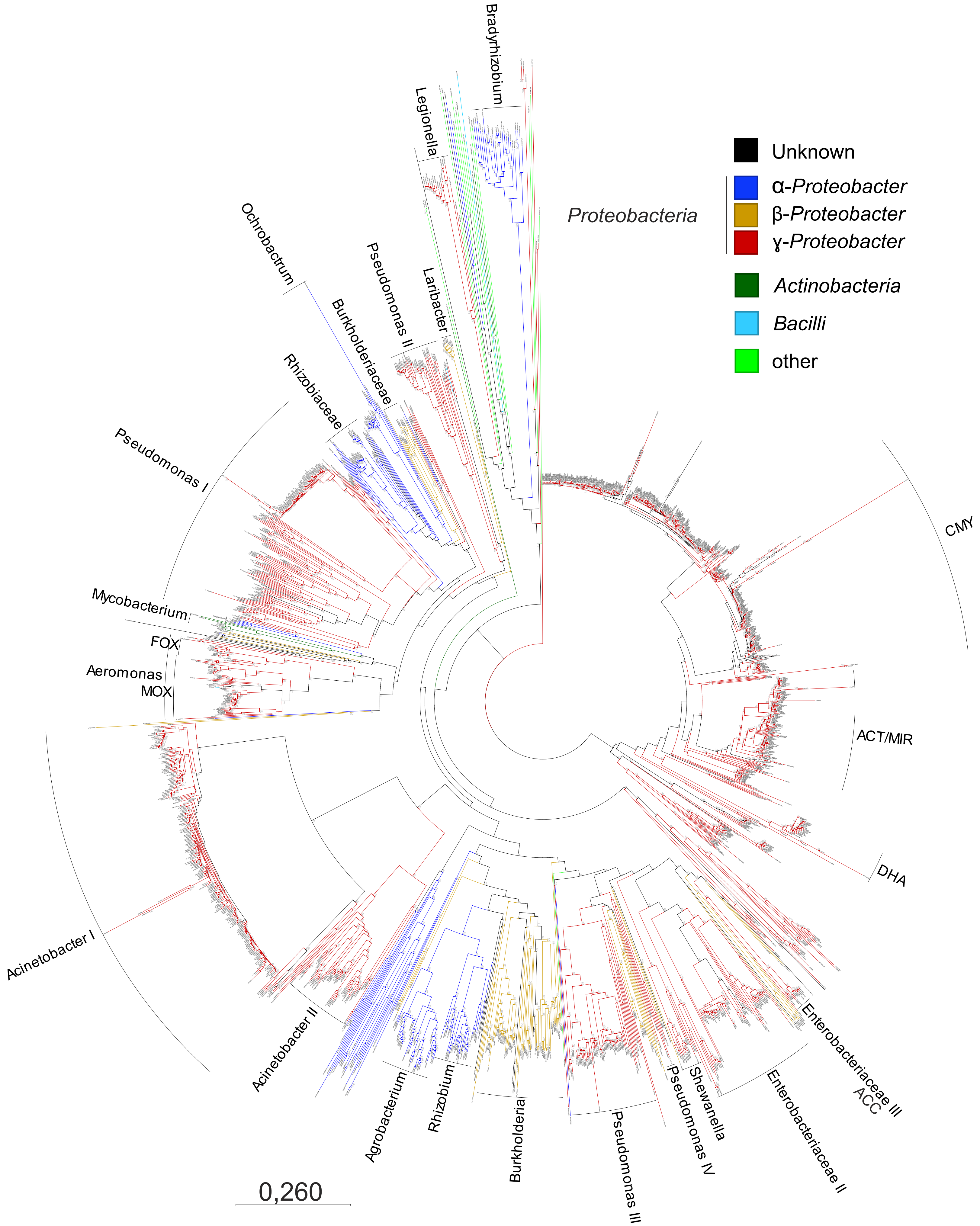

Unknown
α-Proteobacter
β-Proteobacter
γ-Proteobacter
Proteobacteria
Actinobacteria
Bacilli
other
Bradyrhizobium
Legionella
Ochrobactrum
Pseudomonas II
Laribacter
Burkholderiaceae
Rhizobiaceae
Pseudomonas I
Mycobacterium
FOX
Aeromonas
MOX
Acinetobacter I
Acinetobacter II
Agrobacterium
Rhizobium
Burkholderia
Pseudomonas III
Pseudomonas IV
Shewanella
Enterobacteriaceae II
Enterobacteriaceae III
ACC
DHA
ACT/MIR
CMY
0,260

Supplementary Figure S9

High resolution tree of Manuskript Figure 6

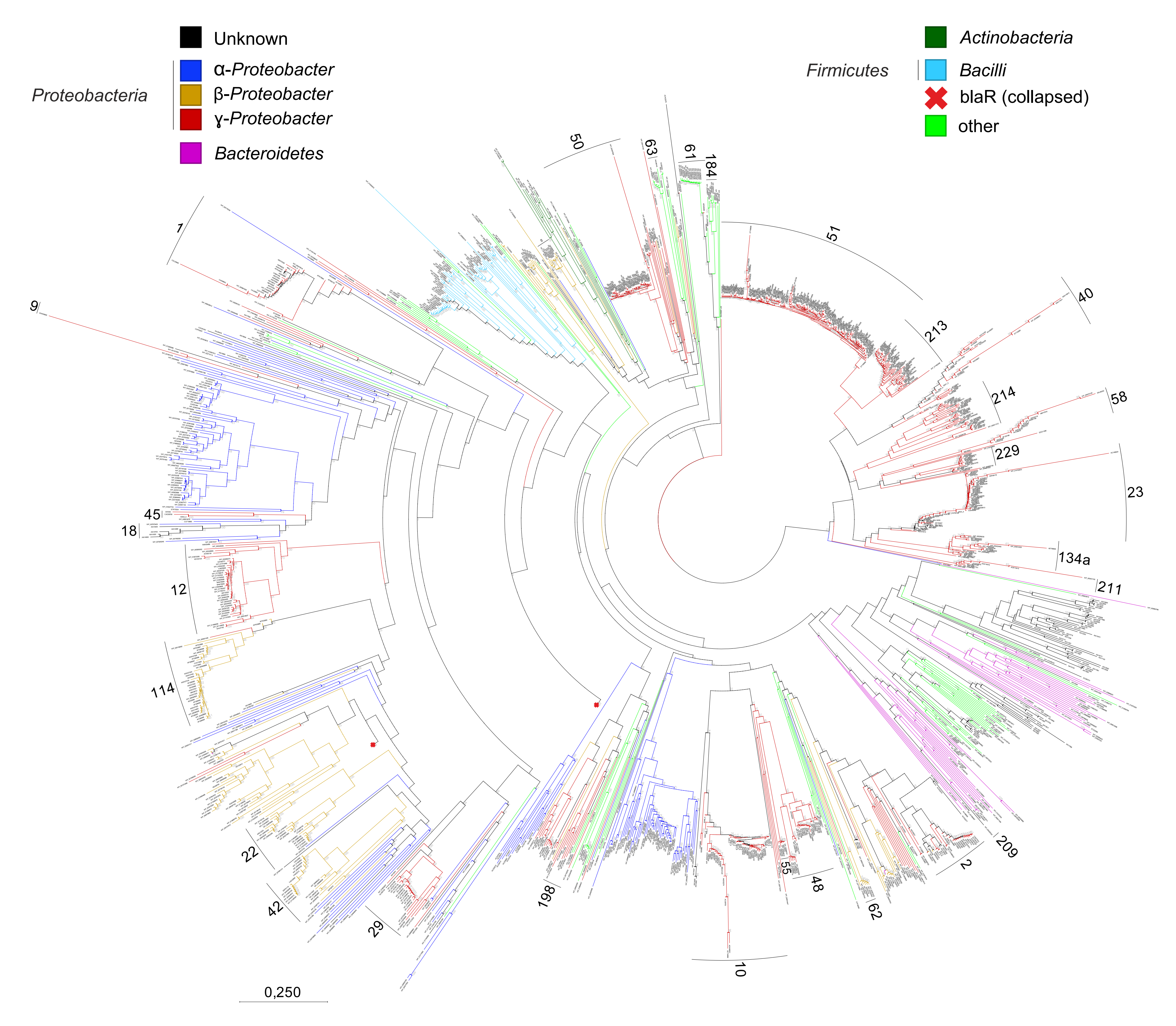
Unknown
Proteobacteria
α-Proteobacter
β-Proteobacter
ɣ-Proteobacter
Bacteroidetes
Actinobacteria
Firmicutes
Bacilli
blaR (collapsed)
other
1
9
50
63
61
184
51
40
213
214
58
229
23
134a
211
45
18
12
114
22
42
29
198
10
55
48
62
2
209
0,250
